# Optimization of B97-Type Density Functional Approximation, Global Hybrid, and Range-Separated Hybrid Energy Functionals with the D4 Dispersion Corrections in TAO-DFT

**DOI:** 10.1021/acs.jctc.5c01037

**Published:** 2025-09-29

**Authors:** Shaozhi Li, Jeng-Da Chai

**Affiliations:** † Department of Physics, 33561 National Taiwan University, Taipei 10617, Taiwan; ‡ Center for Theoretical Physics and Center for Quantum Science and Engineering, 33561 National Taiwan University, Taipei 10617, Taiwan; § Physics Division, National Center for Theoretical Sciences, Taipei 10617, Taiwan

## Abstract

For multireference systems, Kohn–Sham density functional theory (KS-DFT) with the conventional exchange–correlation functionals can yield qualitative failures. Recently, thermally-assisted-occupation density functional theory (TAO-DFT) [Chai, J.-D. *J. Chem. Phys.* **2012**, 136, 154104] has been developed to address this challenge. In this work, to greatly improve the performance of the conventional density functional approximation (DFA), global hybrid (GH), and range-separated hybrid (RSH) functionals in TAO-DFT, we propose the reoptimized B97-type DFA, GH, and RSH functionals with the D4 dispersion corrections in TAO-DFT, yielding TAO-B97-D4, TAO-B97X-D4, and TAO-ωB97X-D4, respectively, wherein a newly proposed analytical parametrization of the optimal system-independent fictitious temperature θ is adopted. Also, with the constraint θ = 0 in parameter optimization, we propose the reoptimized B97-type RSH functional with the D4 dispersion corrections in KS-DFT, denoted as KS-ωB97X-D4. Besides, within TAO-DFT, we propose an efficient method, denoted as pTAO/TDA, to obtain excitation energies, without the issues of spurious excitations. The performance of the resulting functionals has been examined on a wide variety of test sets, including both single-reference systems (e.g., the GMTKN55 database and equilibrium geometries) and multireference systems (e.g., the iso-C_40_ database and linear acenes). Moreover, we examine their performance on some challenging test sets, such as the dissociation of H_2_^+^ and He_2_^+^, dissociation of H_2_ and N_2_, and long-range charge-transfer excitations. Overall, KS-ωB97X-D4 yields high accuracy for the properties (e.g., thermochemistry, kinetics, and noncovalent interactions) of single-reference systems, while TAO-ωB97X-D4, which achieves reasonably good performance for both single-reference and multireference systems, is preferable for general applications.

## Graphical abstract

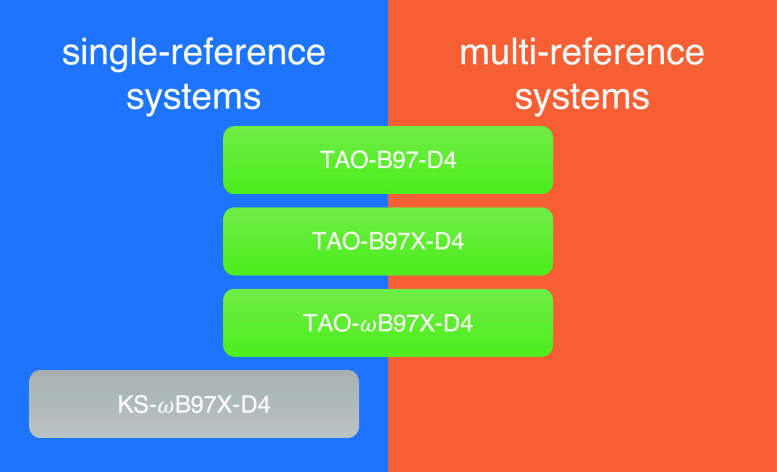

## 1 Introduction

Over the past 30 years, Kohn–Sham density functional theory (KS-DFT)^1,2^ has been a commonly used electronic structure method for studying the ground-state (GS) properties of large electronic systems because of its decent balance between accuracy and computational efficiency.^3–5^ Recently, time-dependent density functional theory (TDDFT)^6–9^ (i.e., a significant extension of KS-DFT) and a frequency-domain formulation of linear-response TDDFT (denoted as LR-TDDFT) have been actively developed for studying the time-dependent (TD) and excited-state (ES) properties, respectively, of large electronic systems.

However, as the exact exchange–correlation (xc) energy functional, which is the key ingredient of KS-DFT and adiabatic TDDFT/LR-TDDFT, remains unknown, an approximate xc energy functional has to be adopted for practical calculations using KS-DFT and adiabatic TDDFT/LR-TDDFT. Nonetheless, in several situations,^10–12^ calculations using an approximate xc energy functional can yield incorrect results. For example, the xc energy functionals based on the conventional density functional approximations (DFAs), such as the local density approximation (LDA)^13,14^ and generalized gradient approximations (GGAs),^15–19^ have been widely used for exploring the GS properties of large electronic systems due to their computational efficiency. Nevertheless, KS-DFT with the conventional DFA xc energy functional (denoted as KS-DFA) cannot appropriately capture the nonlocal xc effects associated with electronic systems, leading to three well-known qualitative errors: the self-interaction error (SIE),^10,11,20–28^ noncovalent interaction error (NCIE),^29–32^ and static correlation error (SCE).^10,11,33,34^

The SIE of KS-DFA is due to the lack of nonlocal exchange energy. As a result, the SIE can be reduced by the global hybrid (GH)^35–43^ and range-separated hybrid (RSH)^44–66^ xc energy functionals, incorporating the exact Hartree–Fock (HF) exchange energy into the parent DFA xc energy functionals. In addition, the NCIE of KS-DFA is due to the lack of medium- and long-range dynamical correlation energy. Accordingly, the NCIE can be reduced by the van der Waals (vdW) density functionals,^30,31^ incorporating a fully nonlocal density-based vdW correlation energy functional into the parent DFA xc energy functionals. Besides, the NCIE can also be reduced by the double-hybrid (DH) xc energy functionals,^67–82^ incorporating both the HF exchange energy and second-order Mo̷ller–Plesset (MP2) correlation energy^83^ into the parent DFA xc energy functionals. Alternatively, to reduce the NCIE, the semiempirical dispersion correction schemes (e.g., the popular -D2, -D3, and -D4 dispersion correction schemes of Grimme et al.)^84–89^ can be directly added to the parent DFA xc energy functionals without extra computational cost, offering a very efficient description of noncovalent interactions for large electronic systems. In other words, to reduce both the SIE and NCIE problems, one can resort to the KS-GH and KS-RSH (i.e., KS-DFT with the GH and RSH xc energy functionals, respectively) with the dispersion correction schemes or the KS-DH (i.e., KS-DFT with the DH xc energy functional), yielding a computational cost comparable with that of the HF or MP2 method. For finite-sized systems (e.g., atoms, molecules, and finite-sized nanosystems), where isolated boundary conditions and Gaussian basis functions are commonly adopted, it is feasible to perform calculations using the aforementioned KS-GH and KS-RSH with the dispersion correction schemes or KS-DH for systems containing several thousands of electrons.

By contrast, the SCE of KS-DFA is due to the lack of static correlation energy. Hence, the SCE problems can occur when KS-DFA is applied to study multireference (MR) systems (i.e., systems with MR character in the electronic ground states), wherein static correlation effects can be pronounced. Note that MR systems (also called strongly correlated electron systems) are systems where the electronic GS wave functions cannot be adequately written as single Slater determinants. For MR systems, KS-DFT with the commonly used xc energy functionals, such as KS-DFA, KS-GH, KS-RSH, and KS-DH, can lead to unreliable results because of the inappropriate description of strong static correlation effects.^10,11,33,34^ Within the framework of KS-DFT, the random phase approximation (RPA) xc energy functionals^4,25,34^ can be employed to describe strong static correlation effects. However, the computational cost of performing KS-RPA (i.e., KS-DFT with the RPA xc energy functional) calculations can be prohibitively expensive, especially for large electronic systems. Consequently, KS-RPA remains infeasible for exploring the properties of large MR systems. More importantly, however, the GS densities of MR systems may not be obtained from KS-DFT (even with the exact xc energy functional).^90–93^ In other words, the GS energies of MR systems may not be obtained with exact KS-DFT because of the issue of GS density representability.

On the other hand, ab initio MR electronic structure methods,^94–111^ such as the complete-active-space self-consistent-field (CASSCF) method,^94^ the complete-active-space second-order perturbation theory (CASPT2),^95^ and related MR electronic structure methods, with considerably large active spaces are typically needed to adequately predict the properties of MR systems. Nevertheless, it is prohibitively high in computational expense to perform reliably accurate MR electronic structure calculations for large electronic systems. Therefore, it remains very impractical to explore the properties of large MR systems using traditional electronic structure methods, such as KS-DFA, KS-GH, KS-RSH, KS-DH, and KS-RPA as well as reliably accurate MR electronic structure methods.

In view of the significance of GS density representability (which has a close relationship with MR systems),^90–93^ thermally-assisted-occupation density functional theory (TAO-DFT)^112^ has recently been developed. TAO-DFT is a density functional theory with fractional orbital occupation numbers produced by the Fermi–Dirac (FD) distribution function with some fictitious temperature θ (i.e., the temperature of the noninteracting reference system in TAO-DFT). Similar to KS-DFT, TAO-DFT is an electronic structure method for studying the GS properties of an electronic system at zero electronic temperature (i.e., θ_el_ = 0). Unlike KS-DFT, the GS density of an electronic system in TAO-DFT is, however, represented with the thermally-assisted-occupation (TAO) orbitals and TAO orbital occupation numbers (TOONs). In contrast to KS-DFT (i.e., TAO-DFT with θ = 0), in TAO-DFT, the fictitious temperature θ can be so chosen that the TAO orbitals and TOONs approximately represent the exact natural orbitals and natural orbital occupation numbers (NOONs),^113^ respectively, to greatly improve the GS density representability.^112^ Hence, for a single-reference system (i.e., a system where the electronic GS wave function can be approximately represented as a single Slater determinant), the fictitious temperature θ in TAO-DFT should be vanishingly small. By contrast, for an MR system, the fictitious temperature θ in TAO-DFT, which is closely related to the strength of MR character (via the distribution of NOONs) and static correlation energy associated with the electronic system, can be highly system-dependent.

Note, however, that the exact exchange–correlation−θ (xcθ) energy functional (i.e., a combined xc and θ-dependent energy functional),^112^ which is the major component of TAO-DFT, has not been known in terms of the GS density. Consequently, an approximate xcθ energy functional needs to be employed for practical TAO-DFT calculations. In the limiting case of θ = 0, since the θ-dependent energy functional is, by definition, absent, TAO-DFT with the xcθ energy functional reduces to KS-DFT with the xc energy functional. With simple approximations [e.g., the LDA and GEA (gradient expansion approximation)] in the θ-dependent energy functionals, the conventional DFA and GH xc energy functionals as well as the semiempirical dispersion correction schemes (e.g., the popular -D2, -D3, and -D4 dispersion correction schemes),^84–89^ recently developed for KS-DFT, can be seamlessly incorporated into TAO-DFT.^112,114,115^ Moreover, when the fictitious temperature θ is properly chosen, the static correlation energy of an electronic system can be approximately described by the entropy contribution in TAO-DFA (i.e., TAO-DFT with the conventional DFA xcθ energy functional)^112,114^ and TAO-GH (i.e., TAO-DFT with the GH xcθ energy functional).^115^ While TAO-RSH (i.e., TAO-DFT with the RSH xcθ energy functional) has been proposed,^115^ the corresponding fictitious temperature θ remains undefined.

It is worth mentioning that relevant generalizations of density functional theory have been developed for studying MR systems in recent years. In TAO-DFT,^112^ the entropy contribution (i.e., the approximate static correlation energy) is expressed in terms of the TOONs (i.e., the approximate NOONs) and fictitious temperature θ. Similar ideas have been recently explored in reduced density matrix functional theory (RDMFT)^116–120^ and natural orbital functional theory (NOFT),^121,122^ wherein the static correlation energy is expressed in terms of the NOONs.

In our recent study,^123^ the TOONs of TAO-DFA (with a properly chosen θ) have been shown to be qualitatively similar to the NOONs of the variational two-electron reduced-density-matrix-driven CASSCF (v2RDM-CASSCF) method (i.e., a reliably accurate CASSCF-related MR electronic structure method),^97,98^ yielding a qualitatively similar trend for the radical nature of 24 alternant polycyclic aromatic hydrocarbons (PAHs) studied.

As aforementioned, it is extremely impractical to study the properties of large MR systems using traditional electronic structure methods (e.g., KS-DFA, KS-GH, KS-RSH, KS-DH, KS-RPA, and reliably accurate MR electronic structure methods). By contrast, since TAO-DFT (with a fictitious temperature θ) is as computationally efficient as KS-DFT (i.e., TAO-DFT with θ = 0), TAO-DFT appears very promising for studying large electronic systems, especially for large MR systems. Owing to its computational efficiency and reasonable accuracy, TAO-DFT has been employed to study the GS properties of a wide variety of MR nanosystems over the past few years.^124–148^

For MR systems, KS-DFA, KS-GH, KS-RSH, and KS-DH can yield incorrect spin orbitals, spin densities, and related properties.^112,114,115^ For instance, for the dissociation of H_2_ and N_2_ (i.e., singlet GS systems with pronounced MR character), the spin-restricted and spin-unrestricted solutions obtained from the same xc energy functional in KS-DFT can be drastically different, leading to the unphysical spin-symmetry breaking effects in the spin-unrestricted solutions. Very recently, a response theory^149^ based on TAO-DFT has been proposed to demonstrate that for a singlet GS system, TAO-DFT with a sufficiently large fictitious temperature θ can always resolve the aforementioned spin-symmetry breaking problems (which are “challenging” for KS-DFT due to the issues of GS density representability). For a singlet GS system, this finding provides a nonempirical scheme defining the optimal system-dependent fictitious temperature θ in TAO-DFT. For example, the optimal θ of a singlet GS system can be defined as the minimum θ with which the spin-restricted and spin-unrestricted solutions obtained from the same xcθ energy functional in TAO-DFT are identical.

Within TAO-DFT, methods for the determination of fictitious temperature θ of an electronic system have also been actively developed in recent years. For example, for TAO-DFA, we have recently proposed a self-consistent approach^150^ to determine the optimal θ of an electronic system, yielding improved performance for a wide range of applications (e.g., for both single-reference and multireference systems). Besides, we have also developed a simple, nonempirical model^151^ to define the optimal system-independent θ of an xcθ energy functional in TAO-DFT. In particular, we have employed this simple model to determine the optimal system-independent θ of TAO-GH as a function of the fraction of exact exchange. Note that TAO-GH (with 0% exact exchange) reduces to TAO-DFA. Furthermore, we have also adopted TAO-DFT with 100% exact exchange to explore the electronic properties of cyclic carbon chains. Owing to the reduced SIE, TAO-DFT with 100% exact exchange can accurately predict the MR character and bond length alternation of cyclic carbon chains (with even number of carbon atoms), showing consistency with the results of reliably accurate electronic structure methods.

Several extensions of TAO-DFT have also been developed in recent years. For example, we have recently proposed a frequency-domain formulation of linear-response time-dependent TAO-DFT (TDTAO-DFT or more precisely, LR-TDTAO-DFT, for its linear-response nature),^152^ based on a pure-state formalism, to obtain the ES properties of MR systems. However, the pure-state formalism of TDTAO-DFT (or LR-TDTAO-DFT) is generally inconsistent with the ensemble formalism of TAO-DFT (i.e., the underlying GS theory), except only for the θ = 0 case.

To resolve the aforementioned inconsistency of TDTAO-DFT, very recently, we have also developed a real-time (RT) extension of TAO-DFT (RT-TAO-DFT),^153^ based on an ensemble formalism (i.e., not a pure-state formalism), which is consistent with TAO-DFT (i.e., the underlying GS theory) for all fictitious temperatures (i.e., θ ≥ 0). RT-TAO-DFT allows the study of TD properties of both single-reference and multireference systems. For the TD properties (e.g., the induced dipole moment and high-order harmonic generation spectrum) of H_2_ at a stretched geometry (i.e., an MR system), aligned along the polarization of an intense linearly polarized laser pulse, spin-unrestricted TDDFT has been shown to yield the unphysical spin-symmetry breaking effects in the TD properties, which can be resolved using spin-unrestricted RT-TAO-DFT.^153^

Moreover, to explore the dynamical information on large MR systems at finite nuclear temperatures, we have recently combined TAO-DFT with ab initio molecular dynamics (AIMD), leading to TAO-DFT-based AIMD (TAO-AIMD).^154^ TAO-AIMD simulations have been performed to predict the instantaneous/average radical nature and infrared spectra of *n*-acenes (i.e., linear acenes with *n* fused benzene rings) at 300 K. Furthermore, to study solvation effects on the GS properties of large MR systems, TAO-DFT has recently been combined with the polarizable continuum model (PCM), yielding TAO-DFT-based PCM (TAO-PCM).^155^ To show its usefulness, TAO-PCM has been employed to explore the electronic properties of linear acenes in various solvents.

In view of the success of B97-type DFA,^18,19,85^ GH,^40–43^ and RSH^55,56,62–66^ xc energy functionals in KS-DFT for many applications, in this work, we intend to optimize the performance of B97-type DFA, GH, and RSH xcθ energy functionals with the D4 dispersion corrections in TAO-DFT as well as the corresponding KS-RSH on a diverse training set, and subsequently apply the resulting functionals to a wide variety of test sets, including both single-reference and multireference systems, to assess their performance.

The rest of this paper is organized as follows. In Section 2, we briefly review TAO-DFT, TAO-DFA, TAO-GH, and TAO-RSH. In Section 3, we determine the optimal system-independent θ values of TAO-DFA, TAO-GH, and TAO-RSH. In Section 4, we define the reoptimized B97-type DFA, GH, and RSH xcθ energy functionals with the D4 dispersion corrections in TAO-DFT, and the reoptimized B97-type RSH xc energy functional with the D4 dispersion corrections in KS-DFT. In Section 5, we propose a TAO-DFT-based method to obtain excitation energies. In Section 6, we examine the performance of the resulting functionals on various test sets. Our conclusions are given in Section 7.

## 2 Overview of TAO-DFT

### 2.1 Spin-Unrestricted Formalism

Consider the GS of a physical system containing *N*_α_ α-spin (e.g., up-spin) and *N*_β_ β-spin (e.g., down-spin) interacting electrons in the presence of an external potential *v*_ext_(**r**) at zero electronic temperature (θ_el_ = 0). In spin-unrestricted (spin-polarized) TAO-DFT,^112^ the α-spin density ρ_α_(**r**) and β-spin density ρ_β_(**r**) of the GS density ρ­(**r**) of the physical system are represented by the thermal equilibrium density distributions ρ_s,α_(**r**) and ρ_s,β_(**r**), respectively, of two reference systems (i.e., one described by the spin function α and the other described by the spin function β) containing noninteracting electrons at the same fictitious temperature θ. The two noninteracting reference systems are often called the TAO reference systems.

To obtain the σ-spin density ρ_σ_(**r**) (with σ = α or β), the two sets (one for each spin function) of self-consistent equations [atomic units (a.u.) are used throughout this work] are given by (*i* runs for the orbital index)^112^

$$\left\{-\frac{1}{2}\nabla^{2}+v_{s,\sigma }^{\mathrm{TAO}}(r)\right\}\psi_{i\sigma }(r)=\epsilon_{i\sigma }\psi_{i\sigma }(r)$$ 1

with the σ-spin effective one-electron potential

$$v_{s,\sigma }^{\mathrm{TAO}}(r)=v_{\mathrm{ext}}(r)+\frac{\delta E_{H}[\rho ]}{\delta \rho (r)}+\frac{\delta E_{\mathrm{xc}\theta }[\rho_{\alpha },\rho_{\beta }]}{\delta \rho_{\sigma }(r)}$$ 2

Here, the first term is the external potential, the second term is the Hartree potential, with

$$E_{H}[\rho ]=\frac{1}{2}\iint \frac{\rho (r)\rho (r^{\prime })}{\left|r-r^{\prime }\right|}drdr^{\prime }$$ 3

being the Hartree energy functional, and the third term is the xcθ potential, with

$$E_{\mathrm{xc}\theta }[\rho_{\alpha },\rho_{\beta }]=E_{x}[\rho_{\alpha },\rho_{\beta }]+E_{c}[\rho_{\alpha },\rho_{\beta }]+E_{\theta }[\rho_{\alpha },\rho_{\beta }]$$ 4

being the xcθ energy functional, where the exchange energy functional *E*_x_[ρ_α_, ρ_β_] and the correlation energy functional *E*_c_[ρ_α_, ρ_β_] are the same as those defined in spin-unrestricted KS-DFT,^2,156,157^ and the θ-dependent energy functional *E*_θ_[ρ_α_, ρ_β_] is defined as

$$E_{\theta }[\rho_{\alpha },\rho_{\beta }]=A_{s}^{\theta =0}[\rho_{\alpha },\rho_{\beta }]-A_{s}^{\theta }[\rho_{\alpha },\rho_{\beta }]$$ 5

with *A*_s_^θ^[ρ_α_, ρ_β_] being the noninteracting kinetic free energy functional (in terms of the spin densities ρ_α_(**r**) and ρ_β_(**r**)) at the fictitious temperature θ. By construction, the σ-spin density ρ_σ_(**r**) is represented by ρ_s,σ_(**r**):

$$\rho_{\sigma }(r)=\rho_{s,\sigma }(r)=\sum_{i=1}^{\infty }f_{i\sigma }|\psi_{i\sigma }(r){|}^{2}$$ 6

Here, *f*_*i*σ_ is the occupation number of the *i*-th σ-spin TAO orbital ψ_*i*σ_(**r**), given by the FD distribution function

$$f_{i\sigma }=\{1+\exp[(\epsilon_{i\sigma }-\mu_{\sigma })/\theta ]{\}}^{-1}$$ 7

where ϵ_*i*σ_ is the energy of the *i*-th σ-spin TAO orbital ψ_*i*σ_(**r**), and μ_σ_ is the σ-spin chemical potential selected to conserve *N*_σ_ (i.e., the number of σ-spin electrons):

$$\sum_{i=1}^{\infty }\{1+\exp[(\epsilon_{i\sigma }-\mu_{\sigma })/\theta ]{\}}^{-1}=N_{\sigma }$$ 8

The GS density ρ­(**r**) is given by

$$\rho (r)=\rho_{\alpha }(r)+\rho_{\beta }(r)=\sum_{\sigma }^{\alpha ,\beta }\rho_{\sigma }(r)$$ 9

The self-consistent process associated with eqs 1, 2 and 6–9 can be adopted to determine the σ-spin TAO orbitals {ψ_*i*σ_(**r**)}, the σ-spin TOONs {*f*_*i*σ_}, the σ-spin density ρ_σ_(**r**), and the GS density ρ­(**r**).^112^

After the self-consistency is achieved, the GS electronic energy of the physical system is computed using

$$E_{\mathrm{TAO‐DFT}}[\rho_{\alpha },\rho_{\beta }]=\int \rho (r)v_{\mathrm{ext}}(r)dr+A_{s}^{\theta }[\{f_{i\alpha },\psi_{i\alpha }\},\{f_{i\beta },\psi_{i\beta }\}]+E_{H}[\rho ]+E_{\mathrm{xc}\theta }[\rho_{\alpha },\rho_{\beta }]$$ 10

where

$$A_{s}^{\theta }[\{f_{i\alpha },\psi_{i\alpha }\},\{f_{i\beta },\psi_{i\beta }\}]=T_{s}^{\theta }[\{f_{i\alpha },\psi_{i\alpha }\},\{f_{i\beta },\psi_{i\beta }\}]+E_{S}^{\theta }[\{f_{i\alpha }\},\{f_{i\beta }\}]$$ 11

is the noninteracting kinetic free energy (in terms of the TAO orbitals and TOONs) at the fictitious temperature θ, which can be exactly computed as the sum of the kinetic energy

$$T_{s}^{\theta }[\{f_{i\alpha },\psi_{i\alpha }\},\{f_{i\beta },\psi_{i\beta }\}]=-\frac{1}{2}\sum_{\sigma }^{\alpha ,\beta }\sum_{i=1}^{\infty }f_{i\sigma }\int \psi_{i\sigma }^{*}(r)\nabla^{2}\psi_{i\sigma }(r)dr$$ 12

and entropy contribution

$$E_{S}^{\theta }[\{f_{i\alpha }\},\{f_{i\beta }\}]=\theta \sum_{\sigma }^{\alpha ,\beta }\sum_{i=1}^{\infty }\{f_{i\sigma }\ln(f_{i\sigma })+(1-f_{i\sigma })\ln(1-f_{i\sigma })\}$$ 13

of noninteracting electrons at the fictitious temperature θ. Note that spin-restricted (spin-unpolarized) TAO-DFT can be formulated by imposing the constraints of ψ_*i*α_(**r**) = ψ_*i*β_(**r**) and *f*_*i*α_ = *f*_*i*β_ to spin-unrestricted TAO-DFT.

In the limiting case of θ = 0, *E*_θ=0_[ρ_α_, ρ_β_] = 0, and hence, spin-unrestricted TAO-DFT with the xcθ energy functional *E*_xcθ_[ρ_α_, ρ_β_] reduces to spin-unrestricted KS-DFT with the xc energy functional *E*_xc_[ρ_α_, ρ_β_]. To obtain the σ-spin density ρ_σ_(**r**) (with σ = α or β), the two sets (one for each spin function) of self-consistent equations (the so-called KS equations) are given by (*i* runs for the orbital index)

$$\left\{-\frac{1}{2}\nabla^{2}+v_{s,\sigma }^{\mathrm{KS}}(r)\right\}\phi_{i\sigma }(r)=\epsilon_{i\sigma }\phi_{i\sigma }(r)$$ 14

with the σ-spin effective one-electron potential

$$v_{s,\sigma }^{\mathrm{KS}}\left(r\right)=v_{\mathrm{ext}}\left(r\right)+\frac{\delta E_{H}\left[\rho \right]}{\delta \rho \left(r\right)}+\frac{\delta E_{\mathrm{xc}}\left[\rho_{\alpha },\rho_{\beta }\right]}{\delta \rho_{\sigma }\left(r\right)}$$ 15

The σ-spin density ρ_σ_(**r**) is computed using the occupied σ-spin KS orbitals {ϕ_*i*σ_(**r**)}

$$\rho_{\sigma }(r)=\sum_{i=1}^{N_{\sigma }}|\phi_{i\sigma }(r){|}^{2}$$ 16

and the GS density ρ­(**r**) is computed using ρ­(**r**) = ∑_σ_^α,β^ρ_σ_(**r**).

After the self-consistency is achieved, for KS-DFT, the GS electronic energy is given by

$$E_{\mathrm{KS‐DFT}}[\rho_{\alpha },\rho_{\beta }]=\int \rho (r)v_{\mathrm{ext}}(r)dr+T_{s}[\{\phi_{i\alpha }\},\{\phi_{i\beta }\}]+E_{H}[\rho ]+E_{\mathrm{xc}}[\rho_{\alpha },\rho_{\beta }]$$ 17

with

$$T_{s}[\{\phi_{i\alpha }\},\{\phi_{i\beta }\}]=-\frac{1}{2}\sum_{\sigma }^{\alpha ,\beta }\sum_{i=1}^{N_{\sigma }}\int \phi_{i\sigma }^{*}(r)\nabla^{2}\phi_{i\sigma }(r)dr$$ 18

being the KS noninteracting kinetic energy.

In short, both KS-DFT (i.e., TAO-DFT with θ = 0) and TAO-DFT (with some θ) are electronic structure methods for the GS properties of physical systems at zero electronic temperature (θ_el_ = 0). If the GS density ρ­(**r**) of a physical system is representable in both KS-DFT and TAO-DFT, the GS electronic energy of the physical system, given by *E*_KS‑DFT_ (see eq 17) in KS-DFT, is the same as *E*_TAO‑DFT_ (see eq 10) in TAO-DFT by construction.^112^ This is due to the fact that the Hohenberg–Kohn (HK) universal functional^1^ can be decomposed into the following sets of energy components in KS-DFT and TAO-DFT, respectively:

$$\begin{array}{ll} F_{\mathrm{HK}}[\rho_{\alpha },\rho_{\beta }] & =T_{s}[\{\phi_{i\alpha }\},\{\phi_{i\beta }\}]+E_{H}[\rho ]+E_{\mathrm{xc}}[\rho_{\alpha },\rho_{\beta }]\\ & =A_{s}^{\theta }[\{f_{i\alpha },\psi_{i\alpha }\},\{f_{i\beta },\psi_{i\beta }\}]+E_{H}[\rho ]+E_{\mathrm{xc}\theta }[\rho_{\alpha },\rho_{\beta }] \end{array}$$ 19

However, for the GS density ρ­(**r**) of an MR system, KS-DFT can have the issues of spin symmetry^112,114,115,149,153^ and GS density representability,^90–93^ while TAO-DFT has an additional degree of freedom in the choice of θ to resolve the issue of spin symmetry,^112,149^ and to greatly improve the GS density representability.^112,153^

On the other hand, while KS-DFT and TAO-DFT are both electronic structure methods for the GS properties of physical systems at zero electronic temperature (θ_el_ = 0), finite-temperature density functional theory (FT-DFT), also known as the Mermin–Kohn–Sham (MKS) method,^2,158^ is an electronic structure method for the thermal equilibrium properties of physical systems at finite electronic temperatures (θ_el_ ≥ 0).^153^ In both KS-DFT and FT-DFT, the fictitious temperature (i.e., the temperature of noninteracting reference systems) θ is assumed to equal the electronic temperature θ_el_ (i.e., θ ≡ θ_el_), while in TAO-DFT, the fictitious temperature θ can be different from the electronic temperature θ_el_. Consequently, for the GS properties of physical systems at zero electronic temperature (θ_el_ = 0), FT-DFT (with θ ≡ θ_el_ = 0) is identical to KS-DFT, while TAO-DFT (with θ > 0) can be drastically different from KS-DFT (especially for MR systems).

### 2.2 TAO-DFA

As the exact xcθ energy functional *E*_xcθ_[ρ_α_, ρ_β_], in terms of the spin densities ρ_α_(**r**) and ρ_β_(**r**), remains unknown, it is essential to develop DFAs for *E*_xcθ_[ρ_α_, ρ_β_] for practical TAO-DFT calculations.

In TAO-DFT, the conventional DFA xcθ energy functionals are semilocal density functionals (e.g., those based on the LDA and GGAs), given by^112,114^

$$E_{\mathrm{xc}\theta }^{\mathrm{DFA}}[\rho_{\alpha },\rho_{\beta }]=E_{x}^{\mathrm{DFA}}[\rho_{\alpha },\rho_{\beta }]+E_{c}^{\mathrm{DFA}}[\rho_{\alpha },\rho_{\beta }]+E_{\theta }^{\mathrm{DFA}}[\rho_{\alpha },\rho_{\beta }]$$ 20

Here, the DFA exchange energy functional *E*_x_^DFA^[ρ_α_, ρ_β_] and the DFA correlation energy functional *E*_c_^DFA^[ρ_α_, ρ_β_] are readily available in spin-unrestricted KS-DFA, and the DFA θ-dependent energy functional

$$E_{\theta }^{\mathrm{DFA}}[\rho_{\alpha },\rho_{\beta }]=A_{s}^{\mathrm{DFA},\theta =0}[\rho_{\alpha },\rho_{\beta }]-A_{s}^{\mathrm{DFA},\theta }[\rho_{\alpha },\rho_{\beta }]$$ 21

can be obtained, once *A*_s_^DFA,θ^[ρ_α_, ρ_β_] (i.e., the DFA noninteracting kinetic free energy functional at the fictitious temperature θ) is available. As of now, the LDA^112^ and GEA^114^ θ-dependent energy functionals (i.e., *E*_θ_^LDA^[ρ_α_, ρ_β_] and *E*_θ_^GEA^[ρ_α_, ρ_β_], respectively) have been proposed.

For TAO-DFA,^112,114^ the GS electronic energy is given by

$$E_{\mathrm{TAO‐DFA}}[\rho_{\alpha },\rho_{\beta }]=\int \rho (r)v_{\mathrm{ext}}(r)dr+A_{s}^{\theta }[\{f_{i\alpha },\psi_{i\alpha }\},\{f_{i\beta },\psi_{i\beta }\}]+E_{H}[\rho ]+E_{\mathrm{xc}\theta }^{\mathrm{DFA}}[\rho_{\alpha },\rho_{\beta }]$$ 22

For the limiting case where θ = 0, since *E*_θ=0_^DFA^[ρ_α_, ρ_β_] = 0, spin-unrestricted TAO-DFT with *E*_xcθ_^DFA^[ρ_α_, ρ_β_] (i.e., spin-unrestricted TAO-DFA) reduces to spin-unrestricted KS-DFT with *E*_xc_^DFA^[ρ_α_, ρ_β_] (i.e., spin-unrestricted KS-DFA).

Since the meta-GGA xc energy functionals^60–62,64,82^ developed in KS-DFT have not been extended to the framework of TAO-DFT, the meta-GGA xcθ energy functionals are not considered in this work.

### 2.3 TAO-GH

TAO-DFA^112,114^ is computationally efficient for large electronic systems, and can outperform KS-DFA (i.e., TAO-DFA with θ = 0) for a wide variety of MR systems.^123–126,129^ However, similar to KS-DFA, the SIE of TAO-DFA can remain large for both single-reference and multireference systems. To greatly reduce the SIE of TAO-DFA, the GH and RSH schemes in TAO-DFT have been recently developed.^115^

In TAO-DFT, the exact exchange functional *F*_x_^θ^[ρ_α_, ρ_β_] is defined as the HF exchange free energy functional of the Coulomb operator 1/*r*_12_ at the fictitious temperature θ^115^

$$F_{x}^{\mathrm{HF},\theta }[\{f_{i\alpha },\psi_{i\alpha }\},\{f_{i\beta },\psi_{i\beta }\}]=-\frac{1}{2}\sum_{\sigma }^{\alpha ,\beta }\sum_{i,j=1}^{\infty }f_{i\sigma }f_{j\sigma }\times \iint \frac{1}{r_{12}}\psi_{i\sigma }^{*}(r_{1})\psi_{j\sigma }^{*}(r_{2})\psi_{j\sigma }(r_{1})\psi_{i\sigma }(r_{2})dr_{1}dr_{2}$$ 23

where *r*_12_ = |**r**_1_ – **r**_2_| is the interelectronic distance. Therefore, the GH xcθ energy functional is defined as^115^

$$E_{\mathrm{xc}\theta }^{\mathrm{GH}}[\rho_{\alpha },\rho_{\beta }]=a_{x}F_{x}^{\mathrm{HF},\theta }[\{f_{i\alpha },\psi_{i\alpha }\},\{f_{i\beta },\psi_{i\beta }\}]+(1-a_{x})E_{x}^{\mathrm{DFA}}[\rho_{\alpha },\rho_{\beta }]+E_{c}^{\mathrm{DFA}}[\rho_{\alpha },\rho_{\beta }]+\{a_{x}E_{x,\theta }^{\mathrm{DFA}}[\rho_{\alpha },\rho_{\beta }]+E_{\theta }^{\mathrm{DFA}}[\rho_{\alpha },\rho_{\beta }]\}$$ 24

where *a*_x_ (i.e., a parameter between 0 and 1) is the fraction of exact exchange, and

$$E_{x,\theta }^{\mathrm{DFA}}[\rho_{\alpha },\rho_{\beta }]=F_{x}^{\mathrm{DFA},\theta =0}[\rho_{\alpha },\rho_{\beta }]-F_{x}^{\mathrm{DFA},\theta }[\rho_{\alpha },\rho_{\beta }]$$ 25

can be obtained, once *F*_x_^DFA, θ^[ρ_α_, ρ_β_] (i.e., the DFA exchange free energy functional of the Coulomb operator 1/*r*_12_ at the fictitious temperature θ) is available. As of now, the LDA version of *E*_x,θ_^DFA^[ρ_α_, ρ_β_] (i.e., *E*_x,θ_^LDA^[ρ_α_, ρ_β_]) has been proposed.^115^

For TAO-GH, the GS electronic energy is given by

$$E_{\mathrm{TAO‐GH}}[\rho_{\alpha },\rho_{\beta }]=\int \rho (r)v_{\mathrm{ext}}(r)dr+A_{s}^{\theta }[\{f_{i\alpha },\psi_{i\alpha }\},\{f_{i\beta },\psi_{i\beta }\}]+E_{H}[\rho ]+E_{\mathrm{xc}\theta }^{\mathrm{GH}}[\rho_{\alpha },\rho_{\beta }]$$ 26

For the special case where *a*_x_ = 0, since *E*_xcθ_^GH^[ρ_α_, ρ_β_] (see eq 24) reduces to *E*_xcθ_^DFA^[ρ_α_, ρ_β_] (see eq 20), spin-unrestricted TAO-GH reduces to spin-unrestricted TAO-DFA. Besides, at θ = 0, since *E*_x,θ=0_^DFA^[ρ_α_, ρ_β_] = 0 and *E*_θ=0_^DFA^[ρ_α_, ρ_β_] = 0, spin-unrestricted TAO-GH reduces to spin-unrestricted KS-GH.

### 2.4 TAO-RSH

In TAO-DFT, the RSH xcθ energy functional is defined as^115^

$$E_{\mathrm{xc}\theta }^{\mathrm{RSH}}[\rho_{\alpha },\rho_{\beta }]=F_{x}^{\mathrm{HF},\theta }(I)[\{f_{i\alpha },\psi_{i\alpha }\},\{f_{i\beta },\psi_{i\beta }\}]+E_{x}^{\mathrm{DFA}}(\mathrm{I̅})[\rho_{\alpha },\rho_{\beta }]+E_{c}^{\mathrm{DFA}}[\rho_{\alpha },\rho_{\beta }]+\{E_{x,\theta }^{\mathrm{DFA}}(I)[\rho_{\alpha },\rho_{\beta }]+E_{\theta }^{\mathrm{DFA}}[\rho_{\alpha },\rho_{\beta }]\}$$ 27

where

$$F_{x}^{\mathrm{HF},\theta }(I)[\{f_{i\alpha },\psi_{i\alpha }\},\{f_{i\beta },\psi_{i\beta }\}]=-\frac{1}{2}\sum_{\sigma }^{\alpha ,\beta }\sum_{i,j=1}^{\infty }f_{i\sigma }f_{j\sigma }\times \iint I(r_{12})\psi_{i\sigma }^{*}(r_{1})\psi_{j\sigma }^{*}(r_{2})\psi_{j\sigma }(r_{1})\psi_{i\sigma }(r_{2})dr_{1}dr_{2}$$ 28

is the HF exchange free energy functional of an interelectronic repulsion operator *I*(*r*_12_) at the fictitious temperature θ, *E*_x_^DFA^(*I̅*)­[ρ_α_, ρ_β_] is the DFA exchange energy functional of the complementary operator *I̅*(*r*_12_) = 1/*r*_12_ – *I*(*r*_12_), and

$$E_{x,\theta }^{\mathrm{DFA}}(I)[\rho_{\alpha },\rho_{\beta }]=F_{x}^{\mathrm{DFA},\theta =0}(I)[\rho_{\alpha },\rho_{\beta }]-F_{x}^{\mathrm{DFA},\theta }(I)[\rho_{\alpha },\rho_{\beta }]$$ 29

can be obtained, once *F*_x_^DFA,θ^(*I*)­[ρ_α_, ρ_β_] (i.e., the DFA exchange free energy functional of the operator *I*(*r*_12_) at the fictitious temperature θ) is available.

For TAO-RSH, the GS electronic energy is given by

$$E_{\mathrm{TAO‐RSH}}[\rho_{\alpha },\rho_{\beta }]=\int \rho (r)v_{\mathrm{ext}}(r)dr+A_{s}^{\theta }[\{f_{i\alpha },\psi_{i\alpha }\},\{f_{i\beta },\psi_{i\beta }\}]+E_{H}[\rho ]+E_{\mathrm{xc}\theta }^{\mathrm{RSH}}[\rho_{\alpha },\rho_{\beta }]$$ 30

For the special case where *I*(*r*_12_) = *a*_x_/*r*_12_, *E*_xcθ_^RSH^[ρ_α_, ρ_β_] reduces to *E*_xcθ_^GH^[ρ_α_, ρ_β_], and hence, spin-unrestricted TAO-RSH reduces to spin-unrestricted TAO-GH. Besides, at θ = 0, since *E*_x,θ=0_^DFA^(*I*)­[ρ_α_, ρ_β_] = 0 and *E*_θ=0_^DFA^[ρ_α_, ρ_β_] = 0, spin-unrestricted TAO-RSH reduces to spin-unrestricted KS-RSH.

## 3 Optimal System-Independent θ Values of TAO-DFA, TAO-GH, and TAO-RSH Functionals

### 3.1 TAO-ωDFAX

For the RSH scheme, an interelectronic repulsion operator *I*(*r*_12_) needs to be specified.^44–66^ Here, we employ a widely used *I*(*r*_12_),^55,56,58,61–64,66^ and define the corresponding RSH xcθ energy functionals with the optimal system-independent θ values in TAO-DFT.

From eq 27, we consider an interelectronic repulsion operator *I*(*r*_12_) (denoted as the erfx operator),^55,56,58,61–64,66^ defined as

$$I(r_{12})=\frac{\mathrm{erf}(\omega r_{12})}{r_{12}}+a_{x}\frac{\mathrm{erfc}(\omega r_{12})}{r_{12}}$$ 31

where erf­(ω*r*_12_)/*r*_12_ (denoted as the erf operator) is a long-range (LR) operator, erfc­(ω*r*_12_)/*r*_12_ (denoted as the erfc operator) is a short-range (SR) operator, ω (in units of inverse length) is a range-separation parameter (between 0 and infinity), and *a*_x_ is a dimensionless parameter (between 0 and 1). The complementary operator *I̅*(*r*_12_) is defined as

$$\overset{¯}{I}(r_{12})=\frac{1}{r_{12}}-I(r_{12})=(1-a_{x})\frac{\mathrm{erfc}(\omega r_{12})}{r_{12}}$$ 32

which is a scaled erfc operator.

After substituting eqs 31 and 32 into eq 27, the resulting RSH xcθ energy functional, denoted as the ωDFAX xcθ energy functional, is given by

$$E_{\mathrm{xc}\theta }^{\omega \mathrm{DFAX}}[\rho_{\alpha },\rho_{\beta }]=F_{x}^{\mathrm{LR‐HF},\theta }[\{f_{i\alpha },\psi_{i\alpha }\},\{f_{i\beta },\psi_{i\beta }\}]+a_{x}F_{x}^{\mathrm{SR‐HF},\theta }[\{f_{i\alpha },\psi_{i\alpha }\},\{f_{i\beta },\psi_{i\beta }\}]+(1-a_{x})E_{x}^{\mathrm{SR‐DFA}}[\rho_{\alpha },\rho_{\beta }]+E_{c}^{\mathrm{DFA}}[\rho_{\alpha },\rho_{\beta }]+\{E_{x,\theta }^{\mathrm{LR‐DFA}}[\rho_{\alpha },\rho_{\beta }]+a_{x}E_{x,\theta }^{\mathrm{SR‐DFA}}[\rho_{\alpha },\rho_{\beta }]+E_{\theta }^{\mathrm{DFA}}[\rho_{\alpha },\rho_{\beta }]\}$$ 33

where

$$F_{x}^{\mathrm{LR‐HF},\theta }[\{f_{i\alpha },\psi_{i\alpha }\},\{f_{i\beta },\psi_{i\beta }\}]=-\frac{1}{2}\overset{\alpha ,\beta }{\underset{\sigma }{\sum }}\overset{\infty }{\underset{i,j=1}{\sum }}f_{i\sigma }f_{j\sigma }\times \iint \frac{\mathrm{erf}(\omega r_{12})}{r_{12}}\psi_{i\sigma }^{*}(r_{1})\psi_{j\sigma }^{*}(r_{2})\psi_{j\sigma }(r_{1})\psi_{i\sigma }(r_{2})dr_{1}dr_{2}$$ 34

is the HF exchange free energy functional of the LR operator (i.e., the erf operator) at the fictitious temperature θ

$$F_{x}^{\mathrm{SR‐HF},\theta }[\{f_{i\alpha },\psi_{i\alpha }\},\{f_{i\beta },\psi_{i\beta }\}]=-\frac{1}{2}\sum_{\sigma }^{\alpha ,\beta }\sum_{i,j=1}^{\infty }f_{i\sigma }f_{j\sigma }\times \iint \frac{\mathrm{erfc}(\omega r_{12})}{r_{12}}\psi_{i\sigma }^{*}(r_{1})\psi_{j\sigma }^{*}(r_{2})\psi_{j\sigma }(r_{1})\psi_{i\sigma }(r_{2})dr_{1}dr_{2}$$ 35

is the HF exchange free energy functional of the SR operator (i.e., the erfc operator) at the fictitious temperature θ, *E*_x_^SR‑DFA^[ρ_α_, ρ_β_] is the DFA exchange energy functional of the SR operator

$$E_{x,\theta }^{\mathrm{LR‐DFA}}[\rho_{\alpha },\rho_{\beta }]=F_{x}^{\mathrm{LR‐DFA},\theta =0}[\rho_{\alpha },\rho_{\beta }]-F_{x}^{\mathrm{LR‐DFA},\theta }[\rho_{\alpha },\rho_{\beta }]$$ 36

is the difference between the DFA exchange free energy functional of the LR operator at zero fictitious temperature (*F*_x_^LR‑DFA,θ=0^[ρ_α_, ρ_β_]) and that at the fictitious temperature θ (*F*_x_^LR‑DFA,θ^[ρ_α_, ρ_β_]), and

$$E_{x,\theta }^{\mathrm{SR‐DFA}}[\rho_{\alpha },\rho_{\beta }]=F_{x}^{\mathrm{SR‐DFA},\theta =0}[\rho_{\alpha },\rho_{\beta }]-F_{x}^{\mathrm{SR‐DFA},\theta }[\rho_{\alpha },\rho_{\beta }]$$ 37

is the difference between the DFA exchange free energy functional of the SR operator at zero fictitious temperature (*F*_x_^SR‑DFA,θ=0^[ρ_α_, ρ_β_]) and that at the fictitious temperature θ (*F*_x_^SR‑DFA,θ^[ρ_α_, ρ_β_]). Since *F*_x_^DFA,θ^[ρ_α_, ρ_β_] = *F*_x_^LR‑DFA,θ^[ρ_α_, ρ_β_] + *F*_x_^SR‑DFA,θ^[ρ_α_, ρ_β_], from eq 36

$$\begin{array}{ll} E_{x,\theta }^{\mathrm{LR‐DFA}}[\rho_{\alpha },\rho_{\beta }] & =(F_{x}^{\mathrm{DFA},\theta =0}[\rho_{\alpha },\rho_{\beta }]-F_{x}^{\mathrm{SR‐DFA},\theta =0}[\rho_{\alpha },\rho_{\beta }])-(F_{x}^{\mathrm{DFA},\theta }[\rho_{\alpha },\rho_{\beta }]-F_{x}^{\mathrm{SR‐DFA},\theta }[\rho_{\alpha },\rho_{\beta }])\\ & =E_{x,\theta }^{\mathrm{DFA}}[\rho_{\alpha },\rho_{\beta }]-E_{x,\theta }^{\mathrm{SR‐DFA}}[\rho_{\alpha },\rho_{\beta }]\\ & =E_{x,\theta }^{\mathrm{SR‐DFA}}[\rho_{\alpha },\rho_{\beta }]\;(\mathrm{with}\;\omega =0)\\ & -E_{x,\theta }^{\mathrm{SR‐DFA}}[\rho_{\alpha },\rho_{\beta }] \end{array}$$ 38

Therefore, when *F*_x_^SR‑DFA,θ^[ρ_α_, ρ_β_] is available, one can first obtain *E*_x,θ_^SR‑DFA^[ρ_α_, ρ_β_] (by eq 37), which yields *E*_x,θ_^LR‑DFA^[ρ_α_, ρ_β_] (by eq 38). Since the LDA version of *F*_x_^SR‑DFA,θ^[ρ_α_, ρ_β_] (i.e., *F*_x_^SR‑LDA,θ^[ρ_α_, ρ_β_]) has been recently proposed by Xuan, Chai, and Su,^159^ the corresponding *E*_x,θ_^SR‑LDA^[ρ_α_, ρ_β_] (see eq 37) and *E*_x,θ_^LR‑LDA^[ρ_α_, ρ_β_] (see eq 38) are readily available.

Note that *F*_x_^LR‑HF,θ^[{*f*_*i*α_, ψ_*i*α_}, {*f*_*i*β_, ψ_*i*β_}] and *F*_x_^SR‑HF,θ^[{*f*_*i*α_, ψ_*i*α_}, {*f*_*i*β_, ψ_*i*β_}] in the ωDFAX xcθ energy functional (see eq 33) are explicit functionals of the TAO orbitals and TOONs. To efficiently obtain the σ-spin density ρ_σ_(**r**) (with σ = α or β), in this work, the electronic energy for TAO-DFT with the ωDFAX xcθ energy functional (denoted as TAO-ωDFAX) is minimized with respect to the σ-spin one-electron reduced density matrix (1-RDM)

$$\gamma_{\sigma }^{\mathrm{TAO}}(r,r^{\prime })=\sum_{i=1}^{\infty }f_{i\sigma }\psi_{i\sigma }^{*}(r)\psi_{i\sigma }(r^{\prime })$$ 39

in TAO-DFT (as is usual for TAO-GH and TAO-RSH).^115^ The resulting self-consistent equations for the σ-spin electrons are given by (*i* runs for the orbital index)

$$\left\{-\frac{1}{2}\nabla^{2}+v_{s,\sigma }^{\mathrm{TAO},\mathrm{local}}(r)\right\}\psi_{i\sigma }(r)-\sum_{j=1}^{\infty }f_{j\sigma }\int \frac{\left\{\mathrm{erf}(\omega |r-r^{\prime }|)+a_{x}\mathrm{erfc}(\omega |r-r^{\prime }|)\right\}}{\left|r-r^{\prime }\right|}\psi_{j\sigma }^{*}(r^{\prime })\psi_{i\sigma }(r^{\prime })\psi_{j\sigma }(r)dr^{\prime }=\epsilon_{i\sigma }\psi_{i\sigma }(r)$$ 40

where

$$v_{s,\sigma }^{\mathrm{TAO},\mathrm{local}}(r)=v_{\mathrm{ext}}(r)+\frac{\delta E_{H}[\rho ]}{\delta \rho (r)}+(1-a_{x})\frac{\delta E_{x}^{\mathrm{SR‐DFA}}[\rho_{\alpha },\rho_{\beta }]}{\delta \rho_{\sigma }(r)}+\frac{\delta E_{c}^{\mathrm{DFA}}[\rho_{\alpha },\rho_{\beta }]}{\delta \rho_{\sigma }(r)}+\left\{\frac{\delta E_{x,\theta }^{\mathrm{LR‐DFA}}[\rho_{\alpha },\rho_{\beta }]}{\delta \rho_{\sigma }(r)}+a_{x}\frac{\delta E_{x,\theta }^{\mathrm{SR‐DFA}}[\rho_{\alpha },\rho_{\beta }]}{\delta \rho_{\sigma }(r)}+\frac{\delta E_{\theta }^{\mathrm{DFA}}[\rho_{\alpha },\rho_{\beta }]}{\delta \rho_{\sigma }(r)}\right\}$$ 41

is the local part of the σ-spin effective one-electron potential.

Eqs 40 and 41 along with eqs 6–9 constitute the self-consistent equations for TAO-ωDFAX, yielding the σ-spin TAO orbitals {ψ_*i*σ_(**r**)}, the σ-spin TOONs {*f*_*i*σ_}, the σ-spin density ρ_σ_(**r**), and the GS density ρ­(**r**).^112,115^

After the self-consistency is achieved, the GS electronic energy is computed using

$$E_{\mathrm{TAO‐}\omega \mathrm{DFAX}}[\rho_{\alpha },\rho_{\beta }]=\int \rho (r)v_{\mathrm{ext}}(r)dr+A_{s}^{\theta }[\{f_{i\alpha },\psi_{i\alpha }\},\{f_{i\beta },\psi_{i\beta }\}]+E_{H}[\rho ]+E_{\mathrm{xc}\theta }^{\omega \mathrm{DFAX}}[\rho_{\alpha },\rho_{\beta }]$$ 42

It is worth mentioning that TAO-ωDFAX contains 100% LR-HF exchange free energy functional (see eq 33), also belonging to the long-range corrected (LC) hybrid scheme.^55,56,58,61–64^ Later, TAO-ωDFAX may be referred to as TAO-RSH (with 100% LR-HF exchange) or TAO-RSH, for brevity.

For the special case where ω = 0, the ωDFAX xcθ energy functional (see eq 33) reduces to the GH xcθ energy functional (denoted as the DFAX xcθ energy functional), given by eq 24. Accordingly, at ω = 0, spin-unrestricted TAO-ωDFAX reduces to spin-unrestricted TAO-DFAX. Later, TAO-DFAX may be referred to as TAO-GH,^115^ for brevity.

For the special case where ω = 0 and *a*_x_ = 0, the ωDFAX xcθ energy functional (see eq 33) reduces to the DFA xcθ energy functional (see eq 20). Therefore, at ω = 0 and *a*_x_ = 0, spin-unrestricted TAO-ωDFAX reduces to spin-unrestricted TAO-DFA.

For the θ = 0 case, since *E*_x,θ=0_^LR‑DFA^[ρ_α_, ρ_β_] = *E*_x,θ=0_^SR‑DFA^[ρ_α_, ρ_β_] = *E*_θ=0_^DFA^[ρ_α_, ρ_β_] = 0, spin-unrestricted TAO-ωDFAX reduces to spin-unrestricted KS-ωDFAX, i.e., spin-unrestricted KS-DFT with the ωDFAX

$$E_{\mathrm{xc}}^{\omega \mathrm{DFAX}}[\rho_{\alpha },\rho_{\beta }]=E_{x}^{\mathrm{LR‐HF}}[\{\phi_{i\alpha }\},\{\phi_{i\beta }\}]+a_{x}E_{x}^{\mathrm{SR‐HF}}[\{\phi_{i\alpha }\},\{\phi_{i\beta }\}]+(1-a_{x})E_{x}^{\mathrm{SR‐DFA}}[\rho_{\alpha },\rho_{\beta }]+E_{c}^{\mathrm{DFA}}[\rho_{\alpha },\rho_{\beta }]$$ 43

where

$$E_{x}^{\mathrm{LR‐HF}}[\{\phi_{i\alpha }\},\{\phi_{i\beta }\}]=-\frac{1}{2}\sum_{\sigma }^{\alpha ,\beta }\sum_{i,j=1}^{N_{\sigma }}\iint \frac{\mathrm{erf}(\omega r_{12})}{r_{12}}\phi_{i\sigma }^{*}(r_{1})\phi_{j\sigma }^{*}(r_{2})\phi_{j\sigma }(r_{1})\phi_{i\sigma }(r_{2})dr_{1}dr_{2}$$ 44

is the HF exchange energy functional of the LR operator,

$$E_{x}^{\mathrm{SR‐HF}}[\{\phi_{i\alpha }\},\{\phi_{i\beta }\}]=-\frac{1}{2}\sum_{\sigma }^{\alpha ,\beta }\sum_{i,j=1}^{N_{\sigma }}\iint \frac{\mathrm{erfc}(\omega r_{12})}{r_{12}}\phi_{i\sigma }^{*}(r_{1})\phi_{j\sigma }^{*}(r_{2})\phi_{j\sigma }(r_{1})\phi_{i\sigma }(r_{2})dr_{1}dr_{2}$$ 45

is the HF exchange energy functional of the SR operator, and ϕ_*i*σ_(**r**) is the *i*-th σ-spin KS orbital.

Similarly, to efficiently obtain the σ-spin density ρ_σ_(**r**) (with σ = α or β), the electronic energy for KS-ωDFAX is commonly minimized with respect to the σ-spin 1-RDM

$$\gamma_{\sigma }^{\mathrm{KS}}(r,r^{\prime })=\sum_{i=1}^{N_{\sigma }}\phi_{i\sigma }^{*}(r)\phi_{i\sigma }(r^{\prime })$$ 46

in KS-DFT (as is usual for KS-GH and KS-RSH).^115^ The resulting self-consistent equations are often called the generalized KS (GKS) equations,^4^ wherein the σ-spin density ρ_σ_(**r**) is computed using the occupied σ-spin KS orbitals {ϕ_*i*σ_(**r**)}

$$\rho_{\sigma }(r)=\sum_{i=1}^{N_{\sigma }}|\phi_{i\sigma }(r){|}^{2}$$ 47

and the GS density ρ­(**r**) is computed using ρ­(**r**) = ∑_σ_^α,β^ρ_σ_(**r**).

For KS-ωDFAX, the GS electronic energy is given by

$$E_{\mathrm{KS}‐\omega \mathrm{DFAX}}[\rho_{\alpha },\rho_{\beta }]=\int \rho (r)v_{\mathrm{ext}}(r)dr+T_{s}[\{\phi_{i\alpha }\},\{\phi_{i\beta }\}]+E_{H}[\rho ]+E_{\mathrm{xc}}^{\omega \mathrm{DFAX}}[\rho_{\alpha },\rho_{\beta }]$$ 48

with

$$T_{s}[\{\phi_{i\alpha }\},\{\phi_{i\beta }\}]=-\frac{1}{2}\sum_{\sigma }^{\alpha ,\beta }\sum_{i=1}^{N_{\sigma }}\int \phi_{i\sigma }^{*}(r)\nabla^{2}\phi_{i\sigma }(r)dr$$ 49

being the KS noninteracting kinetic energy. Note that KS-ωDFAX contains 100% LR-HF exchange energy functional (see eq 43), also belonging to the LC hybrid scheme.^55,56,58,61–64^

Similarly, at θ = 0, spin-unrestricted TAO-DFAX reduces to spin-unrestricted KS-DFAX [i.e., KS-DFT with the DFAX xc energy functional *E*_xc_^DFAX^[ρ_α_, ρ_β_] (defined as *E*_xc_^ωDFAX^[ρ_α_, ρ_β_] with ω = 0)], and spin-unrestricted TAO-DFA reduces to spin-unrestricted KS-DFA [i.e., KS-DFT with the DFA xc energy functional *E*_xc_^DFA^[ρ_α_, ρ_β_] (defined as *E*_xc_^ωDFAX^[ρ_α_, ρ_β_] with ω = 0 and *a*_x_ = 0, which is *E*_xc_^DFA^[ρ_α_, ρ_β_]).

### 3.2 TAO-ωLDAX

In particular, by choosing the LDA as the DFAs for all the density functionals in the ωDFAX xcθ energy functional (see eq 33), the ωLDAX xcθ energy functional is defined as

$$E_{\mathrm{xc}\theta }^{\omega \mathrm{LDAX}}[\rho_{\alpha },\rho_{\beta }]=F_{x}^{\mathrm{LR‐HF},\theta }[\{f_{i\alpha },\psi_{i\alpha }\},\{f_{i\beta },\psi_{i\beta }\}]+a_{x}F_{x}^{\mathrm{SR‐HF},\theta }[\{f_{i\alpha },\psi_{i\alpha }\},\{f_{i\beta },\psi_{i\beta }\}]+(1-a_{x})E_{x}^{\mathrm{SR‐LDA}}[\rho_{\alpha },\rho_{\beta }]+E_{c}^{\mathrm{LDA}}[\rho_{\alpha },\rho_{\beta }]+\{E_{x,\theta }^{\mathrm{LR‐LDA}}[\rho_{\alpha },\rho_{\beta }]+a_{x}E_{x,\theta }^{\mathrm{SR‐LDA}}[\rho_{\alpha },\rho_{\beta }]+E_{\theta }^{\mathrm{LDA}}[\rho_{\alpha },\rho_{\beta }]\}$$ 50

For TAO-DFT with the ωLDAX xcθ energy functional (denoted as TAO-ωLDAX), the GS electronic energy is given by

$$E_{\mathrm{TAO}‐\omega \mathrm{LDAX}}[\rho_{\alpha },\rho_{\beta }]=\int \rho (r)v_{\mathrm{ext}}(r)dr+A_{s}^{\theta }[\{f_{i\alpha },\psi_{i\alpha }\},\{f_{i\beta },\psi_{i\beta }\}]+E_{H}[\rho ]+E_{\mathrm{xc}\theta }^{\omega \mathrm{LDAX}}[\rho_{\alpha },\rho_{\beta }]$$ 51

For the θ = 0 case, since *E*_x,θ=0_^LR‑LDA^[ρ_α_, ρ_β_] = *E*_x,θ=0_^SR‑LDA^[ρ_α_, ρ_β_] = *E*_θ=0_^LDA^[ρ_α_, ρ_β_] = 0, spin-unrestricted TAO-ωLDAX reduces to spin-unrestricted KS-ωLDAX, i.e., KS-DFT with the ωLDAX xc energy functional

$$E_{\mathrm{xc}}^{\omega \mathrm{LDAX}}[\rho_{\alpha },\rho_{\beta }]=E_{x}^{\mathrm{LR‐HF}}[\{\phi_{i\alpha }\},\{\phi_{i\beta }\}]+a_{x}E_{x}^{\mathrm{SR‐HF}}[\{\phi_{i\alpha }\},\{\phi_{i\beta }\}]+(1-a_{x})E_{x}^{\mathrm{SR‐LDA}}[\rho_{\alpha },\rho_{\beta }]+E_{c}^{\mathrm{LDA}}[\rho_{\alpha },\rho_{\beta }]$$ 52

As the SR-LDA exchange free energy functional *F*_x_^SR‑LDA,θ^[ρ_α_, ρ_β_] is readily available,^159^ one can obtain *E*_x,θ_^SR‑LDA^[ρ_α_, ρ_β_] (see eq 37), *E*_x,θ_^LR‑LDA^[ρ_α_, ρ_β_] (see eq 38) and *E*_x_^SR‑LDA^[ρ_α_, ρ_β_] = *F*_x_^SR‑LDA,θ=0^[ρ_α_, ρ_β_]. Besides, *E*_c_^LDA^[ρ_α_, ρ_β_][Bibr ref14] and *E*_θ_^LDA^[ρ_α_, ρ_β_][Bibr ref112] are also available. Therefore, TAO-ωLDAX (see eq 50) and KS-ωLDAX (see eq 52) are well-defined.

### 3.3 Optimal System-Independent θ of TAO-ωDFAX

Recently, Chen and Chai (CC) have proposed a simple model^151^ to define the optimal system-independent θ value of an xcθ energy functional in TAO–DFT. In this work, based on CC’s model,^151^ we numerically obtain, and subsequently parametrize the optimal system-independent θ of TAO-ωDFAX (i.e., TAO-RSH) as a function of the range-separation parameter (ω) and the SR-HF exchange fraction (*a*_x_), leading to θ_TAO‑ωDFAX_(ω, *a*_x_).

Based on CC’s θ_*A*_-model,^151^ wherein *n*_HONO_ ≈ 1.8, obtained from the coupled-cluster valence-bond singles and doubles (CCVB-SD) method,^102^ is adopted as the occupation number of the highest occupied natural orbital (HONO) for the GS (i.e., lowest singlet state) of 5-acene, the optimal system-independent θ of TAO-ωDFAX is defined as

$$\begin{array}{ll} \theta_{\mathrm{TAO‐}\omega \mathrm{DFAX}} & ={\left\{2\ln\left(\frac{n_{\mathrm{HONO}}}{2-n_{\mathrm{HONO}}}\right)\right\}}^{-1}\Delta_{\mathrm{HL}}^{\mathrm{KS}‐\omega \mathrm{DFAX}}(5‐\mathrm{acene})\\ & \approx {\left\{2\ln\left(\frac{1.8}{2-1.8}\right)\right\}}^{-1}\Delta_{\mathrm{HL}}^{\mathrm{KS}‐\omega \mathrm{DFAX}}(5‐\mathrm{acene})\\ & \approx 0.227560\Delta_{\mathrm{HL}}^{\mathrm{KS}‐\omega \mathrm{DFAX}}(5‐\mathrm{acene}) \end{array}$$ 53

where Δ_HL_^KS‑ωDFAX^(5-acene) is the HOMO–LUMO (HL) gap for the GS of 5-acene, obtained with spin-restricted KS-ωDFAX (i.e., TAO-ωDFAX (with θ = 0)) on the respective geometry fully optimized at the same level of theory.

Since Δ_HL_^KS‑ωDFAX^(5-acene) naturally depends on the amount of nonlocal HF exchange (characterized by ω and *a*_x_, e.g., see eq 33), θ_TAO‑ωDFAX_ (see eq 53) is a function of ω and *a*_x_. Also, since the choice of DFA functionals has insignificant effects on the optimal θ values,^112,114,115^ in this work, Δ_HL_^KS‑ωDFAX^(5-acene) is approximately given by Δ_HL_^KS‑ωLDAX^(5-acene), which is the HL gap for the GS of 5-acene, obtained with spin-restricted KS-ωLDAX (i.e., TAO-ωLDAX (with θ = 0)) on the respective geometry fully optimized at the same level of theory. Accordingly, the optimal system-independent θ of TAO-ωDFAX is given by

$$\theta_{\mathrm{TAO‐}\omega \mathrm{DFAX}}\approx 0.227560\Delta_{\mathrm{HL}}^{\mathrm{KS‐}\omega \mathrm{LDAX}}(5‐\mathrm{acene})$$ 54

In this work, all calculations are performed with a development version of Q-Chem 6.2.^160^ First, we perform spin-restricted KS-ωLDAX calculations with a given value of ω (0.00, 0.05, 0.10, ..., 0.90, 0.95, and 1.00 bohr^–1^) and a given value of *a*_x_ (0.00, 0.05, 0.10, ..., 0.90, 0.95, and 1.00) to obtain the numerical values (in mhartree) of Δ_HL_^KS‑ωLDAX^(5-acene) and θ_TAO‑ωDFAX_ (given by eq 54), using the 6-31G­(d) basis set and a numerical grid, consisting of 75 Euler–Maclaurin radial grid points and 302 Lebedev angular grid points, denoted as the EML­(75,302) grid. For spin-restricted KS-ωLDAX (with *a*_x_ = 1.00), Δ_HL_^KS‑ωLDAX^(5-acene) = 258.357 mhartree, yielding the maximum value of Δ_HL_^KS‑ωLDAX^(5-acene).

The numerical values (in mhartree) of θ_TAO‑ωDFAX_, given by eq 54, can fit very well to the following function of ω (in bohr^–1^) and *a*_x_

$$\theta_{\mathrm{TAO‐}\omega \mathrm{DFAX}}(\omega ,a_{x})=0.227560\{258.357+A(\omega )(a_{x}-1)+B(\omega )(a_{x}^{2}-1)\}$$ 55

with

$$A(\omega )=A_{1}\frac{e^{-A_{2}\omega^{A_{3}}}(1+A_{7}\omega +A_{8}\omega^{2})}{A_{4}+A_{5}\omega^{A_{6}}}$$ 56

and

$$B(\omega )=B_{1}\frac{\left(1+B_{2}\omega )(1+e^{-B_{3}\omega^{B_{4}}}\right)}{1+e^{(\omega -B_{5})/B_{6}}}$$ 57

being functions of ω only, where the optimized parameters for *A*_*i*_ (*i* = 1, 2, ..., and 8) and *B*_*i*_ (*i* = 1, 2, ..., and 6) are provided in Table 1.

**Table 1:** Optimized Parameters for *A*_1_, *A*_2_, ..., and *A*_8_ (See Eq 56) and *B*_1_, *B*_2_, ..., and *B*_6_ (See Eq. 57)

*i*	1	2	3	4	5	6	7	8
*A*_*i*_	4.91208	3.79800	1.25822	0.0264826	1.26832	2.90908	–5.18081	19.2913
*B*_*i*_	15.8770	0.988866	37.3967	1.99933	0.230133	0.0682653		

Relative to the numerical data (see eq 54), our parametrization (see eq 55) is reliably accurate, as shown in Figure 1. The maximum error in the θ value (i.e., arising from the fitting) is very small (e.g., less than 0.08 mhartree) for all the values of ω and *a*_x_ examined.

**Figure 1:**
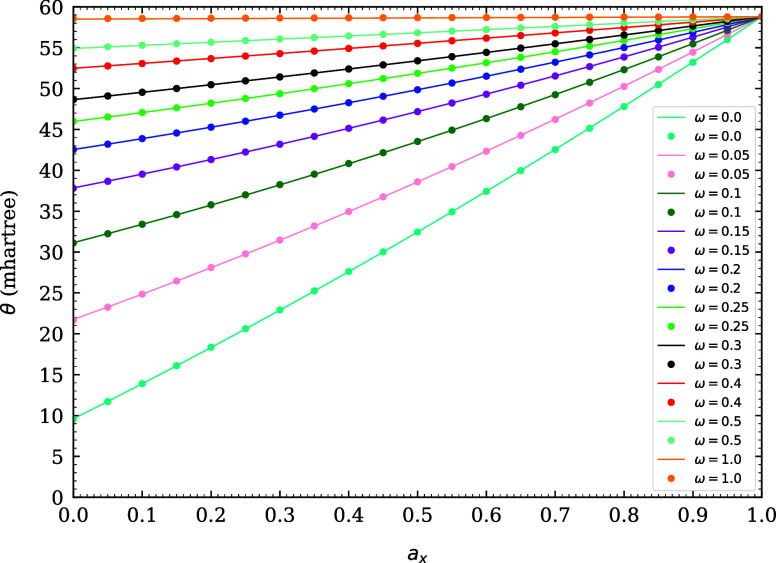
Optimal system-independent fictitious temperature θ (in mhartree) of TAO-ωDFAX (see eqs 33 and 42), as a function of the range-separation parameter ω (in bohr^–1^) and SR-HF exchange fraction *a*_x_. Dots: numerical data (given by eq 54). Lines: analytical parametrization (given by eq 55).

From the analytical parametrization of θ_TAO‑ωDFAX_(ω, *a*_x_) (see eq 55), the optimal system-independent θ (in mhartree) of TAO-DFAX (i.e., TAO-GH^115^), as a function of *a*_x_, is given by

$$\begin{array}{ll} \theta_{\mathrm{TAO‐DFAX}}(a_{x}) & =\theta_{\mathrm{TAO‐}\omega \mathrm{DFAX}}(\omega =0,a_{x})\\ & =0.227560\left\{258.357+\frac{A_{1}}{A_{4}}(a_{x}-1)+\frac{2B_{1}}{1+e^{-B_{5}/B_{6}}}\left(a_{x}^{2}-1\right)\right\} \end{array}$$ 58

As aforementioned, for each *a*_x_, θ_TAO‑DFAX_(*a*_x_) (given by eq 58) is very close to the corresponding numerical data (given by eq 54). Accordingly, θ_TAO‑DFAX_(*a*_x_) is also very close to the optimal system-independent θ of TAO-GH, proposed in our earlier works (e.g., see eq 43 of the TAO-GH paper^115^ and eq 20 of the CC-model paper^151^).

Besides, the optimal system-independent θ (in mhartree) of TAO-DFA is given by

$$\begin{array}{ll} \theta_{\mathrm{TAO‐DFA}} & =\theta_{\mathrm{TAO‐DFAX}}(a_{x}=0)\\ & =\theta_{\mathrm{TAO‐}\omega \mathrm{DFAX}}(\omega =0,a_{x}=0)\\ & =0.227560\left\{258.357-\frac{A_{1}}{A_{4}}-\frac{2B_{1}}{1+e^{-B_{5}/B_{6}}}\right\}\\ & \approx 9.59717 \end{array}$$ 59

yielding the minimum value of θ_TAO‑ωDFAX_(ω, *a*_x_).

In the limiting case of *a*_x_ = 1 or ω → *∞*, since *I*(*r*_12_) = 1/*r*_12_ (see eq 31), the optimal system-independent θ (in mhartree) of TAO-ωDFAX is given by

$$\begin{array}{ll} \theta_{\mathrm{TAO‐}\omega \mathrm{DFAX}}(\omega ,a_{x}=1) & =\theta_{\mathrm{TAO‐}\omega \mathrm{DFAX}}(\omega \to \infty ,a_{x})\\ & =0.227560\{258.357\}\\ & \approx 58.7917 \end{array}$$ 60

yielding the maximum value of θ_TAO‑ωDFAX_(ω, *a*_x_).

## 4 B97-Type DFA, GH, and RSH Energy Functionals with the D4 Dispersion Corrections in TAO-DFT

As shown in Figure 1, the optimal system-independent θ (≈50 mhartree) of TAO-ωDFAX (i.e., TAO-RSH), with ω typically ranging from 0.2 to 0.5 bohr^–1^, is much larger than the optimal system-independent θ (≈10 mhartree) of TAO-DFA and the optimal system-independent θ (≈18–32 mhartree) of TAO-GH (with *a*_x_ typically ranging from 0.2 to 0.5), and hence, it is less likely, for a TAO-RSH functional, to employ a common (system-independent) θ that is optimal for both single-reference and multireference systems. Therefore, in contrast to the TAO-DFA and TAO-GH counterparts,^112,114,115^ it is not suggested to directly transform an existing KS-RSH functional (that was optimized with θ = 0) into a TAO-RSH functional (with θ being given by eq 55), as the corresponding TAO-RSH functional can perform poorly on single-reference systems. Here, we address this issue by the reoptimization of a B97-type TAO-RSH functional with the optimal system-independent θ being given by eq 55.

### 4.1 TAO-B97-D4, TAO-B97X-D4, and TAO-ωB97X-D4

Owing to the flexible functional forms, B97-type DFA,^18,19,85^ GH,^40–43^ and RSH^55,56,62–66^ functionals have been shown to greatly outperform the conventional DFA, GH, and RSH functionals, respectively, in KS-DFT. In view of the success of B97-type DFA,^18,19,85^ GH,^40–43^ and RSH^55,56,62–66^ xc energy functionals in KS-DFT for various applications, here, we optimize the performance of B97-type DFA, GH, and RSH xcθ energy functionals with the D4 dispersion corrections in TAO-DFT on a very diverse training set, wherein the analytical parametrization for the optimal system-independent θ (see eq 55) is employed.

By choosing the LDAs as the DFAs for all the θ-dependent density functionals and choosing the B97-type DFAs (i.e., for the flexible functional forms) as the DFAs for the other density functionals (e.g., (1 – *a*_x_)*E*_x_^SR‑DFA^ is replaced with *E*_x_^SR‑B97^, and *E*_c_^DFA^ is replaced with *E*_c_^B97^)^40,55^ in the ωDFAX xcθ energy functional (see eq 33), the resulting ωB97X xcθ energy functional is defined as

$$E_{\mathrm{xc}\theta }^{\omega \mathrm{B97X}}[\rho_{\alpha },\rho_{\beta }]=F_{x}^{\mathrm{LR‐HF},\theta }\left[\left\{f_{i\alpha },\psi_{i\alpha }\},\{f_{i\beta },\psi_{i\beta }\right\}\right]+a_{x}F_{x}^{\mathrm{SR‐HF},\theta }\left[\left\{f_{i\alpha },\psi_{i\alpha }\},\{f_{i\beta },\psi_{i\beta }\right\}\right]+E_{x}^{\mathrm{SR‐B97}}[\rho_{\alpha },\rho_{\beta }]+E_{c}^{\mathrm{B97}}[\rho_{\alpha },\rho_{\beta }]+\left\{E_{x,\theta }^{\mathrm{LR‐LDA}}[\rho_{\alpha },\rho_{\beta }]+a_{x}E_{x,\theta }^{\mathrm{SR‐LDA}}[\rho_{\alpha },\rho_{\beta }]+E_{\theta }^{\mathrm{LDA}}[\rho_{\alpha },\rho_{\beta }]\right\}$$ 61

Here, the SR-B97 exchange energy functional (i.e., the B97-type DFA exchange energy functional of the SR operator) is given by^55^

$$E_{x}^{\mathrm{SR‐B97}}[\rho_{\alpha },\rho_{\beta }]=\sum_{\sigma }^{\alpha ,\beta }\int e_{x\sigma }^{\mathrm{SR‐LDA}}(\rho_{\sigma })\sum_{i=0}^{m}c_{x\sigma ,i}(\frac{\gamma_{x\sigma }s_{\sigma }^{2}}{1+\gamma_{x\sigma }s_{\sigma }^{2}}{)}^{i}dr$$ 62

where *e*_xσ_^SR‑LDA^(ρ_σ_) is the σ-spin SR-LDA exchange energy density (i.e., the σ-spin LDA exchange energy density of the SR operator),^161^ *s*_σ_ = |∇ρ_σ_|/ρ_σ_^4/3^ is the dimensionless reduced spin density gradient, and γ_xσ_ = 0.004. The B97 correlation energy functional is given by^40^

$$E_{c}^{\mathrm{B97}}[\rho_{\alpha },\rho_{\beta }]=\sum_{\sigma }^{\alpha ,\beta }E_{c\sigma \sigma }^{\mathrm{B97}}[\rho_{\sigma },\rho_{\sigma }]+E_{c\alpha \beta }^{\mathrm{B97}}[\rho_{\alpha },\rho_{\beta }]$$ 63

consisting of the same-spin *E*_cσσ_^B97^ and opposite-spin *E*_cαβ_^B97^ components:

$$E_{c\sigma \sigma }^{\mathrm{B97}}[\rho_{\sigma },\rho_{\sigma }]=\int e_{c\sigma \sigma }^{\mathrm{LDA}}(\rho_{\sigma })\sum_{i=0}^{m}c_{c\sigma \sigma ,i}(\frac{\gamma_{c\sigma \sigma }s_{\sigma }^{2}}{1+\gamma_{c\sigma \sigma }s_{\sigma }^{2}}{)}^{i}dr$$ 64

$$E_{c\alpha \beta }^{\mathrm{B97}}[\rho_{\alpha },\rho_{\beta }]=\int e_{c\alpha \beta }^{\mathrm{LDA}}(\rho_{\alpha },\rho_{\beta })\sum_{i=0}^{m}c_{c\alpha \beta ,i}(\frac{\gamma_{c\alpha \beta }s_{\mathrm{av}}^{2}}{1+\gamma_{c\alpha \beta }s_{\mathrm{av}}^{2}}{)}^{i}dr$$ 65

where γ_cσσ_ = 0.2, γ_cαβ_ = 0.006, and *s*_av_^2^ = (*s*_α_^2^ + *s*_β_^2^)/2. The correlation energy densities *e*_cσσ_^LDA^(ρ_σ_) = *e*_c_^LDA^(ρ_σ_, 0) and *e*_cαβ_^LDA^(ρ_α_, ρ_β_) = *e*_c_^LDA^(ρ_α_, ρ_β_) – *e*_c_^LDA^(ρ_α_, 0) – *e*_c_^LDA^(0, ρ_β_) are derived from the PW92 parametrization^14^ of the LDA correlation energy density *e*_c_^LDA^(ρ_α_, ρ_β_), using the approach of Stoll, Pavlidou, and Preuss.^162,163^ For TAO-DFT with the ωB97X xcθ energy functional (denoted as TAO-ωB97X), the GS electronic energy is given by

$$E_{\mathrm{TAO‐}\omega \mathrm{B97X}}[\rho_{\alpha },\rho_{\beta }]=\int \rho (r)v_{\mathrm{ext}}(r)dr+A_{s}^{\theta }[\{f_{i\alpha },\psi_{i\alpha }\},\{f_{i\beta },\psi_{i\beta }\}]+E_{H}[\rho ]+E_{\mathrm{xc}\theta }^{\omega \mathrm{B97X}}[\rho_{\alpha },\rho_{\beta }]$$ 66

For the special case where ω = 0, the ωB97X xcθ energy functional (see eq 61) reduces to the B97X xcθ energy functional, given by

$$E_{\mathrm{xc}\theta }^{\mathrm{B97X}}[\rho_{\alpha },\rho_{\beta }]=a_{x}F_{x}^{\mathrm{HF},\theta }\left[\left\{f_{i\alpha },\psi_{i\alpha }\},\{f_{i\beta },\psi_{i\beta }\right\}\right]+E_{x}^{\mathrm{B97}}[\rho_{\alpha },\rho_{\beta }]+E_{c}^{\mathrm{B97}}[\rho_{\alpha },\rho_{\beta }]+\left\{a_{x}E_{x,\theta }^{\mathrm{LDA}}[\rho_{\alpha },\rho_{\beta }]+E_{\theta }^{\mathrm{LDA}}[\rho_{\alpha },\rho_{\beta }]\right\}$$ 67

Here, the B97 exchange energy functional is given by^40^

$$E_{x}^{\mathrm{B97}}[\rho_{\alpha },\rho_{\beta }]=\sum_{\sigma }^{\alpha ,\beta }\int e_{x\sigma }^{\mathrm{LDA}}(\rho_{\sigma })\sum_{i=0}^{m}c_{x\sigma ,i}(\frac{\gamma_{x\sigma }s_{\sigma }^{2}}{1+\gamma_{x\sigma }s_{\sigma }^{2}}{)}^{i}dr$$ 68

where *e*_xσ_^LDA^(ρ_σ_) is the σ-spin LDA exchange energy density.^13^ Accordingly, at ω = 0, spin-unrestricted TAO-ωB97X reduces to spin-unrestricted TAO-B97X (i.e., TAO-DFT with the B97X xcθ energy functional).

For the special case where ω = 0 and *a*_*x*_ = 0, the ωB97X xcθ energy functional (see eq 61) reduces to the B97 xcθ energy functional, given by

$$E_{\mathrm{xc}\theta }^{\mathrm{B97}}[\rho_{\alpha },\rho_{\beta }]=E_{x}^{\mathrm{B97}}[\rho_{\alpha },\rho_{\beta }]+E_{c}^{\mathrm{B97}}[\rho_{\alpha },\rho_{\beta }]+E_{\theta }^{\mathrm{LDA}}[\rho_{\alpha },\rho_{\beta }]$$ 69

Therefore, at ω = 0 and *a*_x_ = 0, spin-unrestricted TAO-ωB97X reduces to spin-unrestricted TAO-B97 (i.e., TAO-DFT with the B97 xcθ energy functional).

Following the DFT-D4 scheme,^88,89^ our total energy is given by

$$E_{\mathrm{DFT‐D4}}=E_{\mathrm{total}}^{\mathrm{TAO‐DFT}}+E_{\mathrm{disp}}^{\mathrm{D4}}$$ 70

where *E*_total_^TAO‑DFT^ is the total energy (i.e., the sum of the GS electronic energy and the nuclear–nuclear repulsion energy) obtained with TAO-DFT, and

$$E_{\mathrm{disp}}^{\mathrm{D4}}=-\frac{1}{2}\sum_{n=6,8}s_{n}\sum_{\mathrm{AB}}\frac{C_{\mathrm{AB},n}}{R_{\mathrm{AB}}^{n}+(a_{1}R_{\mathrm{AB},0}+a_{2}{)}^{n}}+E_{\mathrm{disp}}^{\mathrm{ATM}}$$ 71

is the D4 dispersion correction. Here, the first term is an empirical atomic-pairwise dispersion energy (with the Becke–Johnson (BJ) damping function^164–167^ ), the second term *E*_disp_^ATM^ is the Axilrod–Teller–Muto (ATM) dispersion energy (i.e., for three-body dispersion contributions),^168,169^ and *R*_AB_ is the interatomic distance of atom pair AB, while the dispersion coefficients (e.g., *C*_AB,6_ and *C*_AB,8_) for atom pair AB and the cutoff radius *R*_AB,0_ = (*C*_AB,8_/*C*_AB,6_)^1/2^ are provided in the DFT-D4 scheme.^88,89^ Therefore, *s*_6_, *s*_8_, *a*_1_, and *a*_2_, which control the strength of dispersion correction, are the D4 parameters to be determined.

In this work, TAO-B97 (see eq 69), TAO-B97X (see eq 67), and TAO-ωB97X (see eq 61) with the D4 dispersion corrections (see eq 71) are denoted as TAO-B97-D4, TAO-B97X-D4, and TAO-ωB97X-D4, respectively. Note that TAO-B97-D4, TAO-B97X-D4, and TAO-ωB97X-D4 are the B97-type TAO-DFA, TAO-GH, and TAO-RSH functionals, respectively, with the D4 dispersion corrections. We determine the optimized parameters for TAO-B97-D4, TAO-B97X-D4, and TAO-ωB97X-D4 by a diverse training set and some physical constraints, which are described as follows.

Recently, extensive databases containing accurate benchmark values, such as GMTKN55 ^170^ and MGCDB84,^171^ have been compiled to comprehensively assess the performance of KS-DFT functionals (i.e., the xc energy functionals in KS-DFT). The GMTKN55 database^170^ (for general main-group thermochemistry, kinetics, and noncovalent interactions) comprises 55 subsets, which are grouped into the following five categories:

- basic properties and reaction energies for small systems (basic + small);
- reaction energies for large systems and isomerization reactions (iso. + large);
- reaction barrier heights (barriers);
- intermolecular noncovalent interactions (intermol. NCIs);
- intramolecular noncovalent interactions (intramol. NCIs).

To assess the overall performance of a density functional for GMTKN55, two weighted total mean absolute deviation (WTMAD) schemes were also proposed alongside this database. In particular, we focus on the second WTMAD scheme (denoted as WTMAD-2, e.g., see eq 2 of the GMTKN55 paper^170^) in this work. The weight for data in the *i*-th subset of the database is defined as

$$w_{i}=\frac{56.84}{|\Delta E{|}_{\mathrm{avg},i}}$$ 72

where |Δ*E*|_avg,*i*_ (e.g., see Table 1 of the GMTKN55 paper^170^) is the average relative absolute reference energy (in kcal/mol) of the *i*-th subset, and the constant 56.84 (in kcal/mol) is the average of |Δ*E*|_avg,*i*_ over all the subsets. The dominance of subsets with a relatively larger ARARE (average relative absolute reference energy) in the overall assessment can be effectively weakened by WTMAD-2 based on eq 72.

To achieve a balanced performance of the resulting functionals, we select several subsets (e.g., the W4–11, G21EA, G21IP, BSR36, BH76, BHPERI, S22, RG18, HEAVY28, and BUT14DIOL subsets)^86,170,172–182^ from each category of the GMTKN55 database^170^ to compose our training set, as summarized in Table 2. Note, however, that instead of adopting the original W4–11 subset,^183^ its updated version W4–17 (W4–17-nonMR + W4–17-MR)^184^ is incorporated into our training set. Reactions^183^ generated based on the atomization energies within W4–17 are also incorporated into our training set. For these reactions, the geometries and reaction details are taken from MGCDB84,^171^ constituting the BDE (BDE99nonMR + BDE99MR), HAT (HAT707nonMR + HAT707MR), ISO20 (ISOMERIZATION20), and SN13 subsets. Their updated reference values are derived using the revised atomization energies from W4–17. Besides, the ATOM17 subset, including 17 atomic energies with the reference values taken from MGCDB84,^171,185^ is also incorporated into our training set.

**Table 2:** Training Sets Adopted for the Parametrization of TAO-B97-D4, TAO-B97X-D4, TAO-ωB97X-D4, and KS-ωB97X-D4^a^

category	subset	description	weight
basic + small	W4–17^b^	total atomization energies	1.0000^c^ (1.0000)^c^
	BDE^d^	bond dissociation energies	0.5944 (0.5405)
	HAT^d^	heavy-atom transfer energies	0.9208 (0.9487)
	ISO20^d^	isomerization energies	1.7853 (1.7007)
	SN13^d^	nucleophilic substitution energies	2.4240
	G21EA	adiabatic electron affinities	1.6905
	G21IP	adiabatic ionization potentials	0.2206
iso. + large	BSR36	bond-separation energies (saturated hydrocarbons)	3.5093
barriers	BH76	hydrogen and non-hydrogen transfer barrier heights	3.0535
	BHPERI	barrier heights (pericyclic reactions)	2.7231
intermol. NCIs	S22^e^	binding energies (noncovalently bound dimers)	7.7837
	RG18	interaction energies (rare-gas complexes)	98.0000
	HEAVY28^f^	interaction energies (heavy element hydrides)	45.7870
intramol. NCIs	BUT14DIOL	relative energies (butane-1,4-diol conformers)	20.3022
	ATOM17^g^	atomic energies (H and Li–Ar)	0.1000^c^

a The reference values, geometries, and reaction informations are mainly taken from the GMTKN55 database,^170^ and the weight of a subset is determined by eq 72, unless noted otherwise. The weight of the subset excluding the MR data, which is adopted only for the parametrization of KS-ωB97X-D4, is given in parentheses

b The reference values, geometries, and reaction informations are taken from the W4–17 paper.^184^

c The weights of W4–17 and ATOM17 subsets are assigned in this work, not determined by eq 72.

d The geometries and reaction details are taken from the MGCDB84 paper,^171^ while the reference values are derived based on the atomization energies of the W4–17 paper.^184^

e The geometries and reaction details are taken from the S22 paper,^182^ while the reference values are taken from the GMTKN55 paper.^170^

f The HEAVY28 subset is incorporated into the training set only in stage three of the parametrization process.

g The reference values are taken from the AE18 subset of MGCDB84.^171^

In this work, the weighted least-squares method is applied to determine the optimized parameters for each functional. Accordingly, for training data in the parametrization, the weights of subsets (see Table 2) in our training set are determined by eq 72, except for the weights of W4–17 and ATOM17 subsets, which are assigned to 1.0 and 0.1, respectively.

The parameters of TAO-B97-D4, TAO-B97X-D4, and TAO-ωB97X-D4 involve

- the fictitious temperature (θ);
- the range-separation parameter (ω);
- the SR-HF exchange fraction (*a*_x_);
- the B97 coefficients (*c*_xσ,*i*_, *c*_cσσ,*i*_, and *c*_cαβ,*i*_, with *i* = 0, 1, ..., *m*);
- the D4 coefficients (*s*_6_, *s*_8_, *a*_1_, and *a*_2_).

The physical constraints imposed on these parameters are described as follows. First, the optimal system-independent fictitious temperature θ, which depends on the range-separation parameter ω and SR-HF exchange fraction *a*_x_, is given by the analytical parametrization of θ_TAO‑ωDFAX_(ω, *a*_x_) (see eq 55). Second, the exact uniform electron gas (UEG) limit is preserved with the following constraints:

$$a_{x}+c_{x\sigma ,0}=1$$ 73

$$c_{c\sigma \sigma ,0}=1$$ 74

and

$$c_{c\alpha \beta ,0}=1$$ 75

Besides, to preserve the correct vdW asymptote, we also enforce the following constraint:

$$s_{6}=1$$ 76

Moreover, for TAO-B97X-D4, the additional constraint of ω = 0 is applied, while for TAO-B97-D4, the additional constraints of ω = 0 and *a*_x_ = 0 are applied.

In principle, by minimizing the weighted total root-mean-square (WTRMS) error (i.e., a function of θ, ω, *a*_x_, B97 coefficients, *s*_6_, *s*_8_, *a*_1_, and *a*_2_) of the training set (see Table 2 for the weights of subsets), subject to the constraints of θ (given by eq 55), the UEG limit (given by eqs 73–75), and the correct vdW asymptote (given by eq 76), the optimal θ, ω, *a*_x_, B97 coefficients, *s*_6_, *s*_8_, *a*_1_, and *a*_2_ of TAO-ωB97X-D4 can be obtained. However, this approach is computationally demanding due to the need for optimization in high-dimensional parameter spaces. In this work, for efficiency, the parameter optimization is conducted in the following three stages of the parametrization process. Note that only in stage three, the HEAVY28 subset is incorporated into our training set for systems including heavy elements.

In stage one, the optimal *s*_6_ is given by the constraint *s*_6_^*^ = 1 (see Eq. 76), and *s*_8_ is approximately given by *s*_8_^†^ = 1. Motivated by the approach of Chai and Head-Gordon,^55^ for each combination of ω, *a*_*x*_, *a*_1_, and *a*_2_, the fictitious temperature θ (given by Eq. 55) is fixed, and the linear expansion coefficients (i.e., B97 coefficients) are optimized using a self-consistent process described as follows. In the first cycle of the self-consistent process, the TAO-ωLDAX orbitals are used as the initial guess orbitals for the weighted least-squares fitting. We then obtain, for each combination of ω, *a*_x_, *a*_1_, and *a*_2_, a new set of B97 coefficients. With this new set of B97 coefficients, the corresponding self-consistent orbitals can be obtained and then used for another weighted least-squares fitting. This procedure is repeated, for each combination of ω, *a*_x_, *a*_1_, and *a*_2_, until the energies and the B97 coefficients are sufficiently close to the previous ones. Note that for each cycle, the fictitious temperature θ, given by eq 55, remains the same. When converged, the final WTRMS error is a function of ω, *a*_x_, *a*_1_, and *a*_2_, i.e., WTRMS­(ω, *a*_x_, *a*_1_, *a*_2_).

Specifically, we focus on a range of possible values of ω (0.00, 0.05, 0.10, ..., 0.40, 0.45, and 0.50 bohr^–1^) and *a*_x_ (0.00, 0.05, 0.10, ..., 0.40, 0.45, and 0.50), and optimize the corresponding *a*_1_ and *a*_2_ values with suitable steps. By minimizing WTRMS­(ω, *a*_x_, *a*_1_, *a*_2_), the optimal ω, *a*_x_, *a*_1_, and *a*_2_ can be obtained. However, this scheme remains computationally expensive. To save time, the WTRMS error in the first cycle, denoted as the WTRMS^(1)^ error, is adopted to estimate the final WTRMS error

$$\mathrm{WTRMS}(\omega ,a_{x},a_{1},a_{2})\approx {\mathrm{WTRMS}}^{\left(1\right)}(\omega ,a_{x},a_{1},a_{2})$$ 77

Note that similar ideas have been proposed in the recent developments of B97-type RSH functionals in KS-DFT.^55,56,62^ The minimization of WTRMS^(1)^(ω, *a*_x_, *a*_1_, *a*_2_) error (see eq 77) yields the optimal ω, *a*_x_, *a*_1_, and *a*_2_, which are denoted as ω*, *a*_x_^†^, *a*_1_^†^, and *a*_2_^†^, respectively.

In stage two, we reoptimize the linear parameters *a*_x_^†^ and B97 coefficients for higher numerical precision. With *s*_6_^*^ = 1 and *s*_8_^†^ = 1, for the combination of ω*, *a*_1_^†^, and *a*_2_^†^ (i.e., the parameters optimized in stage one), the aforementioned self-consistent process is reinitialized with *a*_*x*_ = *a*_x_^†^ and θ = θ^†^ (given by eq 55), then converged to determine the optimal *a*_x_ and θ (denoted as *a*_x_^*^ and θ*, respectively) as well as the optimal B97 coefficients. Note that for each cycle in stage two, the fictitious temperature θ, given by eq 55, should also be updated with *a*_x_.

In stage three, for the combination of ω*, *a*_x_^*^, θ* (given by eq 55) and the optimal B97 coefficients (i.e., the parameter ω* was optimized in stage one, while the parameters *a*_x_^*^, θ*, and B97 coefficients were optimized in stage two), with *s*_6_^*^ = 1, we reoptimize the remaining D4 coefficients *s*_8_^†^, *a*_1_^†^, and *a*_2_^†^ (with suitable steps) for higher numerical precision, leading to *s*_8_^*^, *a*_1_^*^, and *a*_2_^*^, respectively.

Results for the training set are obtained with the 6-311++G­(3df,3pd) basis set and EML­(75,302) grid, unless noted otherwise. Spin-restricted theory is used for singlet states and spin-unrestricted theory for others. For the interaction energies of the weakly bound systems, the counterpoise correction^186^ is adopted to reduce the basis set superposition error (BSSE). However, for the noncovalent interactions in the BUT14DIOL, RG18, and HEAVY28 subsets, the def2-QZVP basis set^187,188^ is employed without counterpoise correction, due to the difficulty in separating fragments for certain reactions. Additionally, the def2-ECPs (effective core potentials)^189,190^ are employed for heavy elements in some systems of the HEAVY28 subset.

During the optimization procedure, we found that the statistical errors are not significantly improved for *m* > 3. Therefore, the B97-type functional expansions employed in TAO-B97-D4, TAO-B97X-D4, and TAO-ωB97X-D4 are truncated at *m* = 3. Besides, for TAO-B97-D4, *c*_cαβ,3_ = 0 is enforced during the parametrization process to prevent overfitting. The resulting functionals are TAO-B97-D4, TAO-B97X-D4, and TAO-ωB97X-D4, whose optimal parameters are provided in Table 3.

**Table 3:** Optimized Parameters for TAO-B97-D4, TAO-B97X-D4, TAO-ωB97X-D4, and KS-ωB97X-D4^a^

	TAO-B97-D4	TAO-B97X-D4	TAO-ωB97X-D4	KS-ωB97X-D4
θ (mhartree)	9.59717	24.0964	50.2796	0
ω (bohr^–1^)	0	0	0.30	0.30
*a*_x_	0	0.325932	0.179379	0.186861
*c*_xσ,0_	1	0.674068	0.820621	0.813139
*c*_xσ,1_	0.537594	0.112290	0.316873	0.511660
*c*_xσ,2_	1.031795	2.222343	2.642930	3.153429
*c*_xσ,3_	0.121473	–2.637081	–4.300222	–5.200367
*c*_cσσ,0_	1	1	1	1
*c*_cσσ,1_	5.784226	1.665341	1.703973	–4.622426
*c*_cσσ,2_	–12.27029	–4.567622	–4.114387	11.51487
*c*_cσσ,3_	6.980902	3.475585	2.947861	–8.260839
*c*_cαβ,0_	1	1	1	1
*c*_cαβ,1_	–0.163034	1.053471	–1.686072	–1.918557
*c*_cαβ,2_	–2.744025	–19.08454	–9.111376	2.303426
*c*_cαβ,3_	0	24.22133	19.01400	–0.067677
*s*_6_	1	1	1	1
*s*_8_	1.723715	1.708112	1.317163	1.394831
*a*_1_	0.50	0.50	0.65	0.35
*a*_2_	3.5	4.0	3.0	5.0

a For each TAO-DFT functional, the optimal system-independent fictitious temperature θ, which depends on the range-separation parameter ω and SR-HF exchange fraction *a*_x_, is given by eq 55.

### 4.2 KS-ωB97X-D4

To develop the B97-type KS-RSH functional with the D4 dispersion corrections, we also reoptimize the parameters of TAO-ωB97X-D4 with the constraint θ = 0 (i.e., not given by eq 55) in the aforementioned parametrization process, wherein we adopt the same training set (see Table 2), excluding only the reference data involving MR systems in the W4–17, BDE, HAT, and ISO20 subsets (as KS-RSHs are not expected to perform well for MR systems due to the lack of static correlation). Specifically, for the W4–17 (W4–17-nonMR + W4–17-MR), BDE (BDE99nonMR + BDE99MR), and HAT (HAT707nonMR + HAT707MR) subsets, we exclude the W4–17-MR, BDE99MR, and HAT707MR, respectively, and for the ISO20 subset, we exclude a reference data involving dioxygen chloride and chlorine dioxide (i.e., MR systems). Similarly, the weights of subsets are determined by eq 72, except for the weights of W4–17 and ATOM17 subsets, which are assigned to 1.0 and 0.1, respectively, in this work. The resulting B97-type KS-RSH functional with the D4 dispersion corrections is denoted as KS-ωB97X-D4, whose optimized parameters are also provided in Table 3.

It is worth noting that TAO-ωB97X-D4 (with θ = 0) is also the B97-type KS-RSH functional with the D4 dispersion corrections. However, KS-ωB97X-D4 is not the same as TAO-ωB97X-D4 (with θ = 0), since the parameters (i.e., the range-separation parameter, SR-HF fraction, B97 coefficients, and D4 coefficients) of KS-ωB97X-D4 that were optimized with the constraint θ = 0 are generally different from the parameters of TAO-ωB97X-D4 that were optimized with the constraint θ being given by eq 55.

Similarly, TAO-B97-D4 (with θ = 0) and TAO-B97X-D4 (with θ = 0) are also the B97-type KS-DFA and KS-GH functionals, respectively, with the D4 dispersion corrections, although we do not intend to reoptimize their parameters with the constraint θ = 0 in this work.

### 4.3 Training-Set Performance

Here, we report the training-set performance of TAO-B97-D4, TAO-B97X-D4, TAO-ωB97X-D4, and KS-ωB97X-D4. The error for each entry is defined as (error = theoretical value – reference value). The notation adopted for characterizing statistical errors is as follows: mean signed errors (MSEs), mean absolute errors (MAEs), root-mean-square (RMS) errors, and WTRMS errors.

As presented in Tables 4–7, since the training sets are composed of reference data involving mainly single-reference systems, the best training-set performance is achieved by KS-ωB97X-D4 (WTRMS = 4.15 kcal/mol), followed closely by TAO-B97X-D4 (WTRMS = 5.85 kcal/mol) and TAO-ωB97X-D4 (WTRMS = 5.91 kcal/mol). On the other hand, a noticeably larger WTRMS error is found for TAO-B97-D4 (WTRMS = 10.28 kcal/mol), indicating that the inclusion of nonlocal HF exchange in the DFA functional is necessary to greatly improve the training-set performance.

**Table 4:** Statistical Errors (in kcal/mol) of the Training Set for TAO-B97-D4^a^

	MSE	MAE	RMS
W4–17 (200)	–1.96	10.12	13.72
BDE (99)	–1.28	4.89	6.39
HAT (707)	0.60	6.66	8.42
ISO20 (20)	0.42	3.18	4.48
SN13 (13)	3.18	3.18	3.64
G21EA (25)	7.67	8.10	9.46
G21IP (36)	8.53	9.15	11.10
BSR36 (36)	–2.38	2.38	2.68
BH76 (76)	–5.90	6.16	7.03
BHPERI (26)	–4.22	4.22	4.65
S22 (22)	0.84	0.84	1.05
RG18 (18)	0.08	0.11	0.13
HEAVY28 (28)	0.06	0.24	0.31
BUT14DIOL (64)	–0.08	0.22	0.28
ATOM17 (17)	–100.29	100.29	106.96
WTRMS = 10.28			

a The number of data points for each subset is given in parentheses.

**5:** Statistical Errors (in kcal/mol) of the Training Set for TAO-B97X-D4^a^

	MSE	MAE	RMS
W4–17 (200)	–3.43	4.59	6.62
BDE (99)	0.87	3.49	4.52
HAT (707)	–1.19	4.25	5.38
ISO20 (20)	–0.36	1.75	2.22
SN13 (13)	1.11	1.41	1.69
G21EA (25)	0.67	2.58	3.49
G21IP (36)	3.88	5.75	6.81
BSR36 (36)	–2.92	2.92	3.11
BH76 (76)	–2.34	2.53	2.99
BHPERI (26)	–1.46	2.06	2.41
S22 (22)	–0.12	0.32	0.41
RG18 (18)	0.02	0.08	0.09
HEAVY28 (28)	0.08	0.24	0.29
BUT14DIOL (64)	0.14	0.15	0.17
ATOM17 (17)	–3.59	18.93	21.63
WTRMS = 5.85			

a The number of data points for each subset is given in parentheses.

**6:** Statistical Errors (in kcal/mol) of the Training Set for TAO-ωB97X-D4^a^

	MSE	MAE	RMS
W4–17 (200)	–2.61	4.70	6.50
BDE (99)	0.62	3.28	4.06
HAT (707)	–0.63	4.58	5.76
ISO20 (20)	–0.17	1.55	1.92
SN13 (13)	–0.15	0.91	1.14
G21EA (25)	–0.04	2.63	3.38
G21IP (36)	4.23	6.57	8.14
BSR36 (36)	–2.31	2.31	2.45
BH76 (76)	–1.80	2.57	2.99
BHPERI (26)	–0.31	1.86	2.50
S22 (22)	–0.27	0.39	0.56
RG18 (18)	0.01	0.09	0.11
HEAVY28 (28)	0.06	0.26	0.30
BUT14DIOL (64)	0.08	0.10	0.13
ATOM17 (17)	–21.97	21.97	24.71
WTRMS = 5.91			

a The number of data points for each subset is given in parentheses.

**Table 7:** Statistical Errors (in kcal/mol) of the Training Set for KS-ωB97X-D4^a^

	MSE	MAE	RMS
W4–17^b^ (183)	–1.15	2.71	3.83
BDE^b^ (83)	0.17	2.39	3.33
HAT^b^ (505)	–0.11	3.38	4.34
ISO20^b^ (19)	0.17	1.43	1.68
SN13 (13)	–0.84	1.07	1.19
G21EA (25)	–0.22	1.89	2.47
G21IP (36)	1.73	3.35	4.40
BSR36 (36)	–1.09	1.09	1.19
BH76 (76)	–1.26	1.79	2.15
BHPERI (26)	1.04	1.31	2.15
S22 (22)	0.03	0.17	0.23
RG18 (18)	0.02	0.05	0.06
HEAVY28 (28)	0.04	0.14	0.17
BUT14DIOL (64)	0.05	0.05	0.06
ATOM17 (17)	–15.74	16.03	18.23
WTRMS = 4.15			

a The number of data points for each subset is given in parentheses.

b The reference data involving MR systems are excluded from the subsets.

## 5 Post-TAO/TDA Method for Excitation Energies

To obtain excitation energies within the framework of TAO-DFT,^112^ a number of extensions have been recently made. Based on a pure-state formalism (e.g., see Appendix B1 of ref. 152 for the definitions of action functionals in the reference system, wherein the TD pure state is adopted), TDTAO-DFT (or LR-TDTAO-DFT)^152^ has been recently developed for excitation energies. Given that the fictitious temperature θ is typically nonzero, the pure-state formalism of TDTAO-DFT (or LR-TDTAO-DFT) is generally inappropriate, as it is inconsistent with the ensemble formalism of TAO-DFT and the TD density (e.g., see eq 5 of ref. 152) in TDTAO-DFT (or LR-TDTAO-DFT), unless θ = 0.

Later, to be consistent with TAO-DFT (i.e., the underlying GS theory) for all fictitious temperatures (i.e., θ ≥ 0), RT-TAO-DFT^153^ has been developed, based on an ensemble formalism (e.g., see eqs 39, 40, and 44 of ref. 153 for the definitions of action functionals in the reference system, wherein the TD density operator (e.g., see eq 35 of ref. 153) is adopted), for the TD density (e.g., see eqs 37 and 50 of ref. 153) and TD properties of an electronic system. In principle, a frequency-domain formulation of linear-response RT-TAO-DFT (denoted as LR-RT-TAO-DFT) can also be formulated to obtain excitation energies. Basically, the resulting LR-RT-TAO-DFT working equations will be similar to the LR-TDTAO-DFT working equations (e.g., see eqs 20, 21, and 22 of ref. 152), as they differ mainly in the definitions of action functionals in the reference systems. However, similar to the adiabatic approximation in time-dependent density-matrix functional theory (TDDMFT),^191^ when adopting an adiabatic xcθ kernel, spurious excitations can occur in LR-TDTAO-DFT^152^ and LR-RT-TAO-DFT^153^ for a nonzero θ.

In general, to resolve the issue of spurious excitations, the use of a nonadiabatic xcθ kernel, which remains unavailable, in LR-TDTAO-DFT/LR-RT-TAO-DFT is expected to be essential. However, the computational cost of LR-TDTAO-DFT/LR-RT-TAO-DFT with a nonadiabatic xcθ kernel is likely to increase significantly, and hence, the resulting scheme can be impractical for obtaining the excitation energies of large electronic systems.

To address the issue of spurious excitations within the adiabatic approximation, Yeh, Hsu, and Yang have recently proposed the KS-DFA+rTAO method,^192^ i.e., KS-DFA with the rTAO energy correction (i.e., a θ-dependent energy correction evaluated with the KS-DFA spin orbitals). Since the rTAO energy correction is simply a post-KS-DFA energy correction (e.g., see eqs 23 and 24 of ref. 192), the KS-DFA spin orbitals, spin densities, and GS density obtained with KS-DFA+rTAO are the same as those obtained with KS-DFA. Therefore, for adiabatic LR-TDDFT,^6,7,9^ KS-DFA+rTAO and KS-DFA would yield the same excitation energies (i.e., no spurious excitations), since the same adiabatic xc kernel (i.e., the second functional derivative of the DFA xc energy functional) should be adopted. However, in contrast to TAO-DFT, the issues of spin symmetry^112,114,115,149,153^ and GS density representability^90–93^ for MR systems remain unresolved in KS-DFA+rTAO and KS-DFA due to the use of KS reference system.

One of the advantages of TAO-DFT is that the issues of spin symmetry and GS density representability for MR systems can be addressed with a properly chosen θ,^112,114,115,149,153^ thereby justifying the use of TAO orbitals and TAO orbital energies in the subsequent calculations for excitation energies. On the other hand, the Tamm–Dancoff approximation (TDA)^8^ to LR-TDDFT (denoted as LR-TDDFT-TDA or simply as TDA) is a widely used and computationally efficient method for excitation energies, especially for the electronic systems possessing triplet instability, wherein LR-TDDFT^6,7,9^ can encounter the issue of imaginary excitation energies. Both LR-TDDFT and TDA do not have the aforementioned issue of spurious excitations even within the adiabatic approximation,^8,9,152^ due to the satisfaction of the idempotency condition (e.g., see eq 73 of ref. 9) for the density matrix of the KS reference state (i.e., a one-determinantal pure state defined in terms of the occupied KS orbitals). Within this idempotency condition, only transitions between occupied and virtual orbitals are allowed. In this work, we adopt a TAO-DFT-related reference state, which also satisfies the idempotency condition, for the subsequent TDA calculation, to address the issue of spurious excitations within the adiabatic approximation. For clarity, the conventional TDA,^8^ which is based on the KS reference state, is hereafter denoted as KS/TDA.

To combine the best of both worlds, in this work, we propose a post-TAO/TDA method (denoted as pTAO/TDA), to compute the excitation energies of an electronic system. To resolve the issues of spin symmetry and GS density representability for MR systems as well as the issue of spurious excitations within the adiabatic approximation, in the pTAO/TDA method, one first defines the pTAO reference state (i.e., a one-determinantal pure state) in terms of the GS outputs of TAO-DFT (with the xcθ energy functional *E*_xcθ_[ρ_α_, ρ_β_]), for the subsequent TDA calculation. Here, the σ-spin TAO orbitals {ψ_*i*σ_(**r**)} and TAO orbital energies {ϵ_*i*σ_}, obtained with TAO-DFT, are preserved, while the other TAO-DFT outputs are modified as follows. The modified TOONs {*f̃*_*i*σ_} are given by

$${\mathrm{f̃}}_{i\sigma }=\{\begin{array}{ll} 1, & \mathrm{for}\;i\leq N_{\sigma }\\ 0, & \mathrm{for}\;i>N_{\sigma } \end{array}$$ 78

satisfying the normalization constraint

$$\sum_{i=1}^{\infty }{\mathrm{f̃}}_{i\sigma }=N_{\sigma }$$ 79

By construction, the lowest *N*_σ_ σ-spin TAO orbitals are defined as the occupied σ-spin pTAO orbitals, and the remaining σ-spin TAO orbitals are defined as the unoccupied (virtual) σ-spin pTAO orbitals. For the pTAO reference state, the σ-spin density ρ̃_σ_(**r**) can be computed using the occupied σ-spin pTAO orbitals:

$${\mathrm{\rho ̃}}_{\sigma }\left(r\right)=\overset{\infty }{\underset{i=1}{\sum }}{\mathrm{f̃}}_{i\sigma }\left|\psi_{i\sigma }\left(r\right){|}^{2}=\overset{N_{\sigma }}{\underset{i=1}{\sum }}\right|\psi_{i\sigma }\left(r\right){|}^{2}$$ 80

with the total density ρ̃(**r**) being given by

$$\mathrm{\rho ̃}\left(r\right)=\overset{\alpha ,\beta }{\underset{\sigma }{\sum }}{\mathrm{\rho ̃}}_{\sigma }\left(r\right)$$ 81

Accordingly, similar to the KS reference state, the pTAO reference state (i.e., a one-determinantal pure state) is defined in terms of the occupied pTAO orbitals, thereby satisfying the idempotency condition. In general, the pTAO reference state can be regarded as an approximation of the KS reference state for the subsequent TDA calculation. Therefore, the electronic energy of the pTAO reference state (denoted as the pTAO energy) is computed using the KS-DFT electronic energy expression:

$$E_{\mathrm{pTAO}}\left[{\mathrm{\rho ̃}}_{\alpha },{\mathrm{\rho ̃}}_{\beta }\right]=\int \mathrm{\rho ̃}\left(r\right)v_{\mathrm{ext}}\left(r\right)dr+T_{s}\left[\left\{\psi_{i\alpha }\right\},\left\{\psi_{i\beta }\right\}\right]+E_{H}\left[\mathrm{\rho ̃}\right]+E_{\mathrm{xc}}\left[{\mathrm{\rho ̃}}_{\alpha },{\mathrm{\rho ̃}}_{\beta }\right]$$ 82

with

$$T_{s}\left[\left\{\psi_{i\alpha }\right\},\left\{\psi_{i\beta }\right\}\right]=-\frac{1}{2}\overset{\alpha ,\beta }{\underset{\sigma }{\sum }}\overset{N_{\sigma }}{\underset{i=1}{\sum }}\int \psi_{i\sigma }^{*}\left(r\right)\nabla^{2}\psi_{i\sigma }\left(r\right)dr$$ 83

being the KS noninteracting kinetic energy of the occupied pTAO orbitals, and *E*_xc_[ρ̃_α_, ρ̃_β_] ≡ *E*_xcθ_[ρ̃_α_, ρ̃_β_]­(with θ = 0).

At θ = 0, the pTAO reference state is the same as the KS reference state. For a single-reference system, where the optimal θ is sufficiently small, the pTAO reference state should be similar to the KS reference state, and hence, the pTAO energy should be close to the GS electronic energy obtained with KS-DFT. However, for an MR system, the pTAO reference state can be very different from the KS reference state. Note that for a singlet GS system with pronounced MR character, spin symmetry can be preserved with the pTAO reference state (with a properly chosen θ),^112,114,115,149,153^ while the issue of spin symmetry remains unresolved with the KS reference state.

In the pTAO/TDA method, the pTAO reference state (i.e., not the KS reference state^8^ nor the TAO density operator^153^) is adopted as the reference state for the subsequent TDA calculation, employing the adiabatic xc kernel, given by

$$f_{\mathrm{xc}}^{\sigma ,\sigma^{\prime }}(r,r^{\prime })≡\frac{\delta^{2}E_{\mathrm{xc}\theta }[{\mathrm{\rho ̃}}_{\alpha },{\mathrm{\rho ̃}}_{\beta }]}{\delta {\mathrm{\rho ̃}}_{\sigma }(r)\delta {\mathrm{\rho ̃}}_{\sigma^{\prime }}(r^{\prime })}\;(\mathrm{with}\;\theta =0)$$ 84

Similar to KS-DFT, for an electronic system, the lowest energy state for each spin symmetry can be obtained with TAO-DFT. Therefore, TAO-DFT calculations for different spin states will yield the lowest energy state (i.e., the electronic GS) and a few of the excited states. Among these excited states, we define state A_1_ as the first excited state with different spin symmetry from the electronic GS. In the pTAO/TDA method, the electronic energy of state X relative to the electronic GS, which is often referred to as the excitation energy of state X, is computed using

$$\omega_{\mathrm{pTAO}/\mathrm{TDA}}[X]=(\omega [X]-\omega [A_{1}])+(E_{A_{1}}-E_{\mathrm{GS}})$$ 85

where ω­[X] and ω­[A_1_], obtained with TDA, are the electronic energies of state X and state A_1_, respectively, relative to the pTAO reference state, while *E*_A_1__ and *E*_GS_, obtained with TAO-DFT, are the electronic energies of state A_1_ and the electronic GS, respectively. Note that (ω­[X] – ω­[A_1_]) (i.e., the electronic energy of state X relative to state A_1_), obtained with TDA, is adopted to suppress the effect of choice of reference state (e.g., the pTAO reference state), while (*E*_A_1__ – *E*_GS_), obtained with TAO-DFT, accounts for the difference between the electronic energies of state A_1_ and the electronic GS. Accordingly, from eq 85, the electronic energy of state X is given by

$$E_{\mathrm{pTAO}/\mathrm{TDA}}[X]=\omega_{\mathrm{pTAO}/\mathrm{TDA}}[X]+E_{\mathrm{GS}}$$ 86

## 6 Results for the Test Sets

To understand how the resulting functionals: TAO-B97-D4, TAO-B97X-D4, TAO-ωB97X-D4, and KS-ωB97X-D4, perform outside their training sets, we comprehensively examine their performance on a very wide variety of test sets, including both single-reference and multireference systems.

For comparison, the results obtained with the following TAO-DFA and TAO-GH functionals (see Table 8 for the optimal system-independent θ values given by eq 55):

- TAO-DFA: TAO-LDA,^112^ TAO-BLYP,^114^ and TAO-PBE^114^
- TAO-GH: TAO-B3LYP,^115^ TAO-PBE0,^115^ and TAO-BHHLYP^115^

as well as their dispersion-corrected versions (e.g., the D3 model with the zero-damping function (D3(0)),^170^ the D3 model with the BJ-damping function (D3­(BJ)),^170^ and the D4 model^89^). Since the dispersion corrections have no effects on the TAO orbitals and TOONs, the optimal system-independent θ of a TAO-DFT functional (i.e., an xcθ energy functional in TAO-DFT) with the dispersion corrections is the same as that of the TAO-DFT functional without the dispersion corrections.^112,114,115^ As aforementioned, the optimal system-independent θ values of TAO-DFA and TAO-GH functionals (given by eq 55) are very close to those proposed in our earlier works (e.g., see eq 43 of the TAO-GH paper^115^ and eq 20 of the CC-model paper^151^). For single-reference systems, the results obtained with their KS-DFT counterparts (i.e., the θ = 0 cases) are also reported for comparison.

**Table 8:** Optimal System-Independent θ Values (Given by Eq 55) of Various TAO-DFA^112,114^ and TAO-GH^115^ Functionals^a^

	*a*_x_	ω (bohr^–1^)	θ (mhartree)
TAO-LDA	0	0	9.59717
TAO-BLYP	0	0	9.59717
TAO-PBE	0	0	9.59717
TAO-B3LYP	0.20	0	18.3183
TAO-PBE0	0.25	0	20.5859
TAO-BHHLYP	0.50	0	32.4479

a Note that the θ values are very close to those proposed in our earlier works (e.g., see eq 43 of the TAO-GH paper^115^ and eq 20 of the CC-model paper^151^).

### 6.1 Single-Reference Systems

#### 6.1.1 GMTKN55 Database

As noted earlier, the GMTKN55 database^170^ (for general main-group thermochemistry, kinetics, and noncovalent interactions) comprises 55 subsets, which are grouped into five categories. The dominance of subsets with a relatively larger ARARE (average relative absolute reference energy) in the overall assessment can be effectively weakened by WTMAD-2 (i.e., the second WTMAD scheme^170^). Therefore, to assess the performance of the resulting functionals (TAO-B97-D4, TAO-B97X-D4, TAO-ωB97X-D4, and KS-ωB97X-D4) on the GMTKN55 database,^170^ their WTMAD-2 values are reported.

For the GMTKN55 database, all calculations are performed using the EML­(75,302) grid, and our strategy for choosing basis sets is as follows:

- For the categories of (iso. + large) and barriers, the 6-311++G­(3df,3pd) basis set is employed for computational efficiency.
- For the other categories, the def2-QZVP basis set^187,188^ is employed without counterpoise correction, except for the WATER27, G21EA, AHB21, and IL16 subsets, where the def2-QZVPPD basis set^193,194^ is adopted.
- Additionally, the def2-ECPs^189,190^ are adopted for heavy elements in some systems of the HEAVY28, HEAVYSB11, and HAL59 subsets.

Besides, spin-restricted theory is used for singlet states and spin-unrestricted theory for others. For KS-ωB97X-D3,^62^ the WTMAD-2 values of all the categories in the GMTKN55 database, obtained in this work, are comparable to those obtained by Goerigk et al., using different basis sets^170^ (see the Supporting Information (SI) for detailed results). This indicates that the basis-set effects on the WTMAD-2 values reported in this work are small, when compared to those obtained by Goerigk et al.^170^

Table 9 summaries the WTMAD-2 values of all the categories in the GMTKN55 database^170^ for various KS-DFT and TAO-DFT functionals. Since the GMTKN55 database is composed of reference data involving mainly single-reference systems, among the four resulting functionals proposed in this work, KS-ωB97X-D4 performs the best, followed by TAO-B97X-D4 and TAO-ωB97X-D4. A noticeably larger error is found for TAO-B97-D4, implying that the inclusion of nonlocal HF exchange in the TAO-DFA functional is essential to greatly improve its performance on the GMTKN55 database. Based on its performance on the GMTKN55 database, KS-ωB97X-D4 yields high accuracy in thermochemistry, kinetics, and noncovalent interactions. Therefore, the performance of the resulting functionals on the training sets is generally transferable to the GMTKN55 database.

**Table 9:** WTMAD-2 Values (in kcal/mol) of the GMTKN55 Database^170^ ^a^

	basic + small (473)	iso. + large (243)	barriers (194)	intermol. NCIs (304)	intramol. NCIs (291)	all NCIs (595)	all (1505)
KS-BLYP^b^	8.52	23.22	14.56	34.90	29.47	32.25	21.05
KS-BLYP-D3(BJ)^b^	6.88	14.56	15.55	7.10	8.04	7.56	9.51
KS-PBE^b^	6.59	16.23	16.72	15.68	19.70	17.65	13.83
KS-PBE-D3(BJ)^b^	6.51	12.36	18.36	10.21	9.58	9.90	10.32
TAO-BLYP	8.55	22.46	14.24	28.76	27.04	27.92	19.19
TAO-BLYP-D3(0)	7.71	15.88	14.71	8.48	8.71	8.59	10.28
TAO-BLYP-D3(BJ)	7.39	13.90	15.76	11.10	9.35	10.25	10.65
TAO-BLYP-D4	7.28	12.97	16.05	9.94	11.25	10.58	10.64
TAO-PBE	6.94	15.78	16.92	13.44	18.01	15.68	13.11
TAO-PBE-D3(0)	6.89	12.78	17.90	14.49	8.99	11.80	11.20
TAO-PBE-D3(BJ)	7.04	12.12	18.57	15.71	9.91	12.87	11.65
TAO-PBE-D4	6.96	11.75	18.57	16.25	10.17	13.28	11.73
TAO-B97-D4^c^	6.98	12.44	11.69	6.74	7.93	7.32	8.61
KS-B3LYP^b^	5.72	17.53	9.19	28.23	25.14	26.72	16.38
KS-B3LYP-D3(BJ)^b^	4.36	10.28	9.04	5.56	5.68	5.62	6.42
KS-PBE0^b^	4.34	11.57	8.63	15.42	17.94	16.65	10.93
KS-PBE0-D3(BJ)^b^	4.45	8.39	9.88	6.65	6.40	6.53	6.61
KS-BHHLYP^b^	6.14	13.35	9.39	22.76	21.59	22.19	14.07
KS-BHHLYP-D3(BJ)^b^	5.32	7.76	7.73	4.46	4.86	4.66	5.76
KS-PW6B95-D3(BJ)^b^	3.27	9.06	6.70	4.22	6.69	5.43	5.50
TAO-B3LYP	5.38	16.34	9.47	15.03	19.36	17.14	12.33
TAO-B3LYP-D3(0)	4.98	10.98	9.86	16.02	8.54	12.36	9.50
TAO-B3LYP-D3(BJ)	4.95	9.40	10.83	18.98	9.38	14.28	10.12
TAO-B3LYP-D4	4.75	9.09	10.68	17.26	10.36	13.88	9.83
TAO-PBE0	5.54	11.88	10.41	10.91	12.89	11.88	9.70
TAO-PBE0-D3(0)	5.86	9.76	11.50	21.25	9.00	15.26	10.93
TAO-PBE0-D3(BJ)	6.02	9.77	11.91	22.01	9.27	15.78	11.24
TAO-PBE0-D4	5.92	9.64	11.87	21.50	9.68	15.72	11.16
TAO-BHHLYP	6.18	11.74	6.44	11.59	10.61	11.11	9.06
TAO-BHHLYP-D3(0)	6.30	8.29	6.17	30.74	12.63	21.88	12.76
TAO-BHHLYP-D3(BJ)	6.33	7.87	6.35	33.12	12.52	23.05	13.19
TAO-BHHLYP-D4	6.13	7.69	6.18	33.46	13.96	23.92	13.42
TAO-B97X-D4^c^	4.17	7.94	5.68	5.14	4.92	5.03	5.31
KS-ωB97X	3.48	9.03	5.06	6.08	6.92	6.49	5.77
KS-ωB97X-D3	3.34	7.45	4.56	4.60	4.86	4.72	4.71
KS-ωB97X-V^d^	3.35	6.66	4.21	3.03	3.66	3.34	3.99
KS-ωB97M-V^d^	2.74	4.77	3.40	2.86	4.57	3.70	3.53
KS-ωB97X-D4rev^e^	3.18	6.04	3.75	2.84	3.62	3.22	3.73
KS-ωB97X-D4^c^	3.07	5.48	4.09	2.61	3.57	3.08	3.60
TAO-ωB97X-D4^c^	4.81	7.69	5.96	5.95	5.92	5.93	5.87

a The number of data points for each category is given in parentheses.

b Data taken from the SI of the GMTKN55 paper.^170^

c Resulting functionals in this work.

d Full-SCF fashion for the VV10 nonlocal correlation. Data taken from the work of Najibi and Goerigk.^196^

e Data taken from the work of Müller et al.^66^

Relative to the other functionals, the resulting functionals show much improved performance on the GMTKN55 database. For example, TAO-B97-D4 outperforms all the KS-DFA functionals and the other TAO-DFA functionals. TAO-B97X-D4 performs comparably to KS-BHHLYP-D3­(BJ)^35,167^ and KS-PW6B95-D3­(BJ)^167,195^ (i.e., the most accurate KS-GH functional identified in previous study^170^), and consistently outperforms the remaining KS-GH and TAO-GH functionals. TAO-ωB97X-D4 performs comparably to KS-ωB97X^55^ and TAO-B97X-D4. KS-ωB97X-D4 performs comparably to KS-ωB97M-V^64^ (i.e., the most accurate KS-RSH functional identified in previous studies^171,196,197^ ), and consistently outperforms the remaining KS-RSH functionals, including KS-ωB97X,^55^ KS-ωB97X-D3,^62^ KS-ωB97X-V,^63^ and KS-ωB97X-D4rev^66^ for the entire GMTKN55 database. Notably, KS-ωB97X-D4 performs the best in the categories of noncovalent interactions, highlighting the superiority of the D4 model.

Since some subsets of the GMTKN55 database are included in the TAO-ωB97X-D4 training set, to ensure a fair comparison, we define the GMTKN55-test database as the GMTKN55 database excluding the subsets (e.g., the W4–11, G21EA, G21IP, BSR36, BH76, BHPERI, S22, RG18, HEAVY28, and BUT14DIOL subsets) that were incorporated into the TAO-ωB97X-D4 training set. As shown in Table 10, the performance of KS-DFT and TAO-DFT functionals on the GMTKN55 database is generally transferable to the GMTKN55-test database.

**Table 10:** WTMAD-2 Values (in kcal/mol) of the GMTKN55-Test Database (See the Context for the Definition)^170^ ^a^

	basic + small (272)	iso. + large (207)	barriers (92)	intermol. NCIs (236)	intramol. NCIs (227)	all NCIs (463)	all (1034)
KS-BLYP^b^	12.16	20.19	6.21	25.31	35.27	30.19	21.31
KS-BLYP-D3(BJ)^b^	10.03	15.40	6.55	5.05	7.90	6.45	9.19
KS-PBE^b^	9.68	14.37	9.13	13.70	23.77	18.64	14.58
KS-PBE-D3(BJ)^b^	9.64	12.57	9.29	8.58	9.65	9.10	9.96
TAO-BLYP	12.56	19.55	7.03	21.31	32.54	26.82	19.85
TAO-BLYP-D3(0)	11.12	15.64	6.93	7.33	9.05	8.17	10.33
TAO-BLYP-D3(BJ)	10.64	14.88	7.40	8.76	9.07	8.91	10.43
TAO-BLYP-D4	10.51	14.72	7.46	8.36	9.79	9.06	10.43
TAO-PBE	10.17	14.18	9.85	12.26	21.49	16.78	13.91
TAO-PBE-D3(0)	10.08	12.73	9.71	11.02	8.72	9.89	10.49
TAO-PBE-D3(BJ)	10.34	12.64	10.02	12.17	9.55	10.88	11.02
TAO-PBE-D4	10.22	12.73	9.97	12.57	9.15	10.89	11.00
TAO-B97-D4^c^	9.88	13.16	5.86	6.08	8.91	7.47	9.10
KS-B3LYP^b^	8.35	14.05	3.52	20.57	30.06	25.22	16.61
KS-B3LYP-D3(BJ)^b^	6.66	10.02	3.78	4.11	5.51	4.80	6.24
KS-PBE0^b^	6.64	8.95	5.54	12.45	21.68	16.98	11.63
KS-PBE0-D3(BJ)^b^	6.82	7.87	5.70	5.71	6.94	6.31	6.70
KS-BHHLYP^b^	6.83	9.39	5.04	17.03	25.56	21.21	13.63
KS-BHHLYP-D3(BJ)^b^	5.81	6.38	4.57	3.50	5.54	4.50	5.23
KS-PW6B95-D3(BJ)^b^	5.18	8.68	3.17	2.63	6.63	4.59	5.44
TAO-B3LYP	8.13	13.02	4.85	12.25	23.61	17.82	13.16
TAO-B3LYP-D3(0)	7.42	9.98	4.74	11.58	8.60	10.12	8.90
TAO-B3LYP-D3(BJ)	7.35	9.36	5.22	14.07	8.99	11.58	9.46
TAO-B3LYP-D4	7.05	9.52	5.12	13.16	9.16	11.20	9.23
TAO-PBE0	8.17	9.79	6.85	9.76	15.43	12.54	10.33
TAO-PBE0-D3(0)	8.72	9.45	6.90	15.46	9.14	12.36	10.33
TAO-PBE0-D3(BJ)	8.96	9.96	7.17	16.16	9.31	12.80	10.72
TAO-PBE0-D4	8.82	10.12	7.10	15.89	9.27	12.64	10.64
TAO-BHHLYP	7.40	8.02	4.57	7.93	12.63	10.23	8.54
TAO-BHHLYP-D3(0)	7.61	6.60	4.83	21.08	13.61	17.42	11.55
TAO-BHHLYP-D3(BJ)	7.74	7.03	4.96	22.91	13.02	18.06	11.97
TAO-BHHLYP-D4	7.41	7.17	4.79	22.95	14.17	18.64	12.16
TAO-B97X-D4^c^	6.17	7.54	4.00	4.48	5.51	4.98	5.72
KS-ωB97X	5.29	7.04	2.65	4.69	8.13	6.38	5.89
KS-ωB97X-D3	5.08	6.38	2.78	3.09	5.60	4.32	4.79
KS-ωB97X-V^d^	4.96	6.55	2.67	2.02	4.46	3.22	4.29
KS-ωB97M-V^d^	3.57	5.13	2.26	1.88	5.57	3.69	3.82
KS-ωB97X-D4rev^e^	4.82	6.07	2.44	2.03	4.28	3.13	4.10
KS-ωB97X-D4^c^	4.59	5.77	3.11	2.09	4.30	3.17	4.06
TAO-ωB97X-D4^c^	7.32	7.62	4.65	5.27	7.02	6.13	6.61

a The number of data points for each category is given in parentheses.

b Computed using the SI data of the GMTKN55 paper.^170^

c Resulting functionals in this work.

d Full-SCF fashion for the VV10 nonlocal correlation. Computed using the SI data of the work of Najibi and Goerigk.^196^

e Computed using the SI data of the work of Müller et al.^66^

#### 6.1.2 Equilibrium Geometries

For various practical applications, satisfactory predictions of molecular geometries are crucial. We perform geometry optimizations for TAO-B97-D4, TAO-B97X-D4, TAO-ωB97X-D4, and KS-ωB97X-D4, using the 6-311++G­(3df,3pd) basis set and EML­(99,590) grid, on the equilibrium experimental test set (EXTS),^198^ which contains the 166 symmetry unique experimental bond lengths of 136 small- to medium-sized molecules. On the EXTS set, the ground states of the molecules near their equilibrium geometries possess single-reference character. For comparison, the results obtained with other KS-DFT and TAO-DFT functionals are also included.

As presented in Table 11 (also see the SI for more detailed results), the TAO-DFT functionals perform comparably to their KS-DFT counterparts (i.e., the θ = 0 cases). This implies that the optimal system-independent θ values adopted (see eq 55) in the TAO-DFT functionals should be sufficiently small. On the other hand, noticeably larger RMS errors are found for the KS-DFA and TAO-DFA functionals, indicating that the inclusion of nonlocal HF exchange in the KS-DFA and TAO-DFA functionals would be useful in improving their performance on the EXTS set.

**Table 11:** Statistical Errors (in Å) of the 166 Bond Lengths in the EXTS Set^198^

	MSE	MAE	RMS
KS-LDA^a^	0.004	0.013	0.017
KS-BLYP^a^	0.018	0.019	0.024
KS-PBE^a^	0.014	0.015	0.019
TAO-LDA^a^	0.005	0.013	0.017
TAO-BLYP^a^	0.019	0.020	0.025
TAO-PBE^a^	0.014	0.015	0.020
TAO-B97-D4^b^	0.013	0.014	0.020
KS-B3LYP^c^	0.003	0.008	0.013
KS-PBE0^c^	–0.002	0.008	0.012
KS-BHHLYP^c^	–0.012	0.013	0.017
TAO-B3LYP^c^	0.003	0.008	0.013
TAO-PBE0^c^	–0.002	0.008	0.013
TAO-BHHLYP^c^	–0.014	0.015	0.019
TAO-B97X-D4^b^	–0.005	0.010	0.016
KS-ωB97^d^	–0.002	0.010	0.015
KS-ωB97X^d^	–0.003	0.009	0.014
KS-ωB97X-D	–0.002	0.008	0.013
KS-ωB97X-D3	–0.003	0.009	0.013
KS-ωB97X-D4^b^	–0.003	0.008	0.013
TAO-ωB97X-D4^b^	–0.007	0.011	0.015

a Data taken from the TAO-DFA papers.^112,114^

b Resulting functionals in this work.

c Data taken from the TAO-GH paper.^115^

d Data taken from the KS-ωB97 paper.^55^

#### 6.1.3 Dissociation of H_2_^+^ and He_2_^+^

Owing to the severe SIEs of KS-DFAs, unphysical barriers can arise in the dissociation curves of symmetric radical cations (e.g., H_2_^+^ and He_2_^+^).^10,11,20–28^ By incorporating the nonlocal exchange effects, the unphysical barriers of the dissociation curves can be partially reduced by the commonly used KS-GH functionals (e.g., those with 20–50% HF exchange), and greatly eliminated by most KS-RSH functionals (with 100% LR-HF exchange).^55,61,62,65^

To examine the performance of TAO-B97-D4, TAO-B97X-D4, TAO-ωB97X-D4, and KS-ωB97X-D4 upon the SIE problems, the dissociation energy curves of H_2_^+^ and He_2_^+^ are computed using the aug-cc-pVQZ basis set and EML­(250,590) grid.

Note that in TAO-DFT, the optimal θ = 0 should be adopted for the dissociation energy curve of H_2_^+^ (i.e., a one-electron system in the nondegenerate electronic GS) at any internuclear distance *R*, possessing perfect single-reference character. For the dissociation energy curve of He_2_^+^, the electronic GS, at any internuclear distance *R* considered, possesses pronounced single-reference character based on our preliminary calculations using the adaptive sampling configuration interaction (ASCI) method^106^ (see the SI for detailed results). Accordingly, in TAO-DFT, the optimal θ = 0 should also be adopted for He_2_^+^ dissociation.

Therefore, for each TAO-DFT functional, we adopt both the optimal system-independent θ (given by eq 55) and θ = 0 (i.e., the optimal θ for the dissociation of H_2_^+^ and He_2_^+^). For comparison, the exact curve, obtained with the HF theory, for the dissociation of H_2_^+^ and the very accurate curve, obtained with the CCSD­(T) theory (i.e., the coupled-cluster theory with iterative singles and doubles and perturbative treatment of triple substitutions),^199^ for the dissociation of He_2_^+^ are also included.

As shown in Figure 2, for each TAO-DFT functional, near the equilibrium geometries of H_2_^+^ and He_2_^+^, the results of the optimal system-independent θ (given by eq 55) are close to the results of θ = 0 (i.e., the optimal θ for the dissociation of H_2_^+^ and He_2_^+^), while at the stretched geometries of H_2_^+^ and He_2_^+^, the results of θ (given by eq 55) yield more severe SIEs, deviating greatly from the results of θ = 0. For all the θ = 0 cases, due to the reduced SIEs, TAO-ωB97X-D4 and KS-ωB97X-D4, which outperform TAO-B97X-D4 and TAO-B97-D4, yield the qualitatively correct dissociation curves of H_2_^+^ and He_2_^+^ (i.e., with no unphysical barriers). This indicates that even with the optimal system-dependent θ = 0, a TAO-RSH functional remains necessary for obtaining the qualitatively correct dissociation curves of H_2_^+^ and He_2_^+^. It seems necessary to employ a more flexible operator^58^ for HF exchange in the TAO-RSH functional to further reduce such errors.

**Figure 2:**
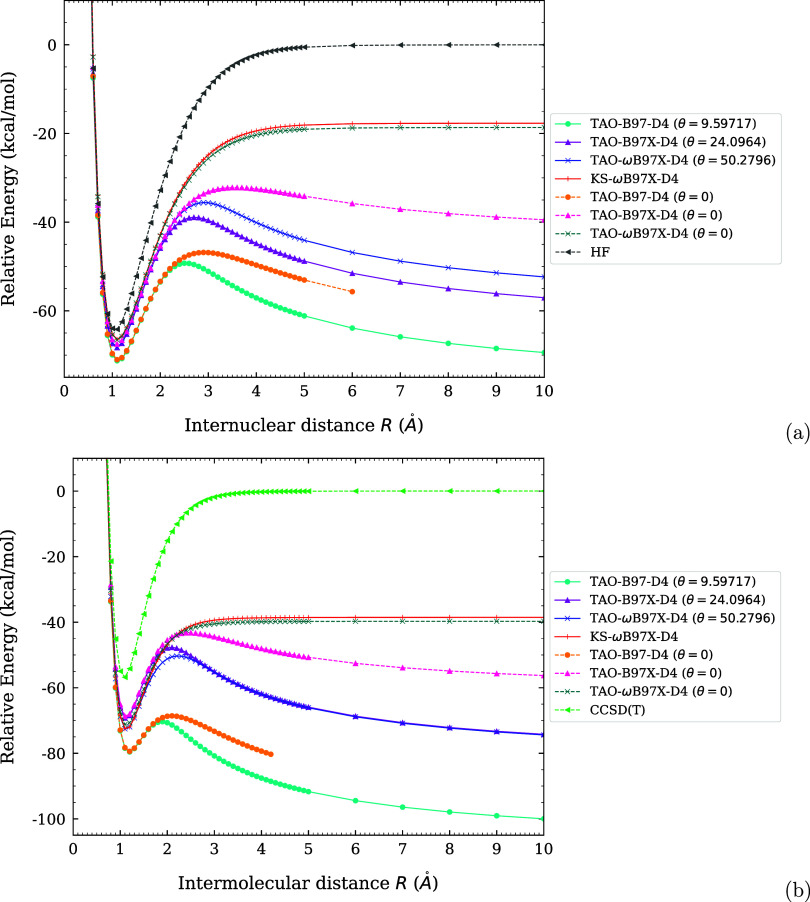
Dissociation curve of X_2_^+^, where X stands for (a) H and (b) He, obtained with spin-unrestricted KS-ωB97X-D4 as well as spin-unrestricted TAO-B97-D4, TAO-B97X-D4, and TAO-ωB97X-D4, with various θ (in mhartree). Zero level is set to *E*(X) + *E*(X^+^) for each method.

In short, to properly describe the dissociation energy curves of H_2_^+^ and He_2_^+^, KS-ωB97X-D4 or TAO-ωB97X-D4 with a system-dependent θ scheme that can yield the optimal θ = 0 for the dissociation of H_2_^+^ and He_2_^+^ should be employed. For the TAO-DFT functionals, this goes beyond the limitations of the system-independent θ scheme (given by eq 55) adopted in this work. As aforementioned, for TAO-DFAs (e.g., TAO-B97-D4), we have recently developed a self-consistent approach^150^ to determine the optimal system-dependent θ, yielding improved performance for diverse applications (e.g., for both single-reference and multireference systems). However, for TAO-GHs (e.g., TAO-B97X-D4) and TAO-RSHs (e.g., TAO-ωB97X-D4), an optimal system-dependent θ scheme remains unavailable, and needs to be developed.

### 6.2 Multireference Systems

#### 6.2.1 Linear Acenes

The nature of linear *n*-acenes (C_4*n*+2_H_2*n*+4_) has attracted much interest from experimental researches.^200–208^ Recently, the largest 13-acene has been successfully synthesized on the Au(111) surface,^203^ creating a milestone in acene chemistry.

Owing to the pronounced static correlation effects in the larger *n*-acenes (e.g., *n* > 5), ab initio MR electronic structure methods^94–111^ are essential to accurately predict the properties of the larger *n*-acenes. These MR electronic structure methods include the configuration interaction (CI)-based approaches,^103,106–109,111^ the density matrix renormalization group (DMRG) algorithm,^96,99,110^ the v2RDM-CASSCF method,^97,98,101,104,105^ the CCVB-SD method,^102^ and many others.^209–213^ However, for the larger *n*-acenes, these MR electronic structure methods can be prohibitively expensive.

On the other hand, KS-DFAs, KS-GHs, and KS-RSHs are computationally efficient for the study of large electronic systems. However, the electronic properties of the larger *n*-acenes, obtained with KS-DFAs, KS-GHs, and KS-RSHs, can be unreliable, since the larger *n*-acenes can possess pronounced MR character in their ground states.^96,101,112,115,124,211^

To remedy this situation, TAO-DFT^112^ has been recently developed to study the electronic properties of large MR systems, such as the larger *n*-acenes. The electronic properties of *n*-acenes, have been recently computed using TAO-DFAs (e.g., TAO-LDA, TAO-BLYP, and TAO-PBE)^112,114,124^ and TAO-GHs (e.g., TAO-B3LYP, TAO-PBE0, and TAO-BHHLYP),^115^ with the results in good agreement with the results of experiments and high-level ab initio electronic structure methods. By contrast, the corresponding KS-DFAs and KS-GHs (i.e., the θ = 0 cases) have been shown to yield unreliable results for the larger *n*-acenes.

Here we assess the performance of TAO-B97-D4, TAO-B97X-D4, TAO-ωB97X-D4, and KS-ωB97X-D4 on the electronic properties of *n*-acenes (*n* = 2–30), using the 6-31G­(d) basis set and EML­(75,302) grid. For comparison, the results obtained with the commonly used KS-RSHs, such as KS-ωB97,^55^ KS-ωB97X,^55^ KS-ωB97X-D,^56^ and KS-ωB97X-D3,^62^ are also included.

The singlet–triplet (ST) energy gap of *n*-acene is computed using

$$E_{\mathrm{ST}}=E_{\mathrm{UT}}-E_{\mathrm{US}}$$ 87

with *E*_UT_/*E*_US_ being the spin-unrestricted energy of the lowest triplet/singlet state of *n*-acene, evaluated at the respective optimized molecular geometry. As indicated by the predicted ST gaps in Figure 3 (also see the SI for more detailed results), *n*-acenes (*n* = 2–30) have singlet ground states, similar to previous findings.^96,101,103,112,114,115,124^ The ST gaps of TAO-B97-D4, TAO-B97X-D4, and TAO-ωB97X-D4 monotonically decrease with increasing acene length, showing consistency with the results of experiments^204–207^ and the reliably accurate DMRG,^96^ ADMRPT2 (also known as ACI-DSRG-MRPT2),^103^ and *w̃*-RDMFT^119^ methods. By contrast, the ST gaps of KS-ωB97, KS-ωB97X, KS-ωB97X-D, KS-ωB97X-D3, and KS-ωB97X-D4 unexpectedly increase beyond 8-acene, due to unphysical spin-symmetry breaking effects (as shown below).

**Figure 3:**
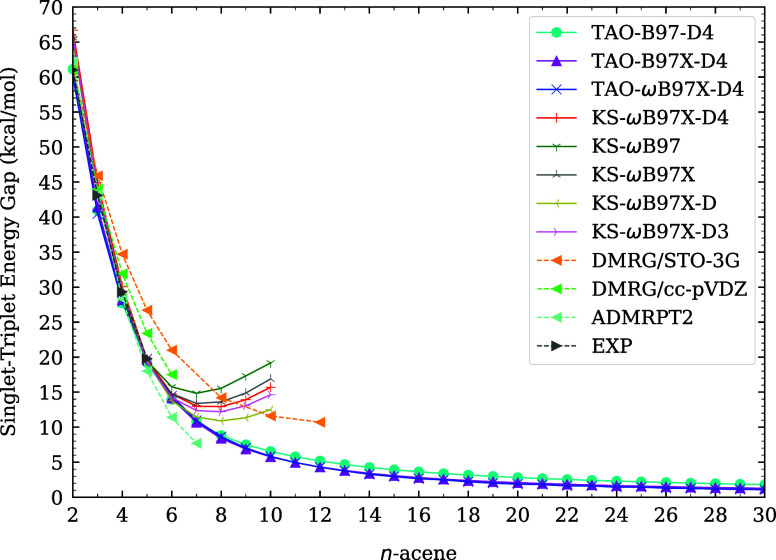
Singlet–triplet energy gap of *n*-acene, obtained with spin-unrestricted TAO-B97-D4, TAO-B97X-D4, TAO-ωB97X-D4, and KS-ωB97X-D4. For comparison, the results of KS-ωB97, KS-ωB97X, KS-ωB97X-D, and KS-ωB97X-D3 are also included. Here, the uncorrected experimental (EXP) data are taken from the works of Birks,^204^ Schiedt et al.,^205^ Sabbatini et al.^206^ and Burgos et al.,^207^ the DMRG data are taken from the work of Hachmann et al.,^96^ and the ADMRPT2 data are taken from the work of Schriber et al.^103^

For an electronic system in the lowest singlet state, spin-symmetry constraint^10–12,112,114,115,149,211^ ensures that spin-restricted and spin-unrestricted calculations should yield identical energies when the exact theory is applied. Consequently, the difference between the spin-restricted energy (*E*_RS_) and spin-unrestricted energy (*E*_US_) of the lowest singlet state (denoted as the RU energy difference Δ*E*_RU_) of the electronic system, given by

$$\Delta E_{\mathrm{RU}}=E_{\mathrm{RS}}-E_{\mathrm{US}}$$ 88

can serve as a quantitative measure of spin-symmetry breaking. To examine the possible spin-symmetry breaking, additional calculations are performed to obtain the spin-restricted energy of the lowest singlet state of *n*-acene, evaluated at the respective optimized molecular geometry. As shown in Figure 4, the RU energy difference Δ*E*_RU_ of *n*-acene, obtained with each TAO-DFT functional, remains very small (with the maximum deviation of 0.1 kcal/mol for 17-acene, obtained with TAO-ωB97X-D4), implying that essentially no unphysical spin-symmetry breaking effects occur in our spin-unrestricted TAO-DFT solutions. By contrast, the RU energy difference Δ*E*_RU_ of *n*-acene, obtained with each KS-DFT functional, dramatically increases with increasing *n* (e.g., Δ*E*_RU_ can exceed 27 kcal/mol for 10-acene, obtained with KS-ωB97), leading to the unphysical spin-symmetry breaking effects in spin-unrestricted KS-DFT solutions for the larger *n*-acenes (e.g., *n* > 5). This indicates that the KS-DFT functionals should be generally inappropriate for studying the larger *n*-acenes, due to the pronounced MR character in their ground states. Accordingly, hereafter the results of KS-DFT functionals are not included for brevity.

**Figure 4:**
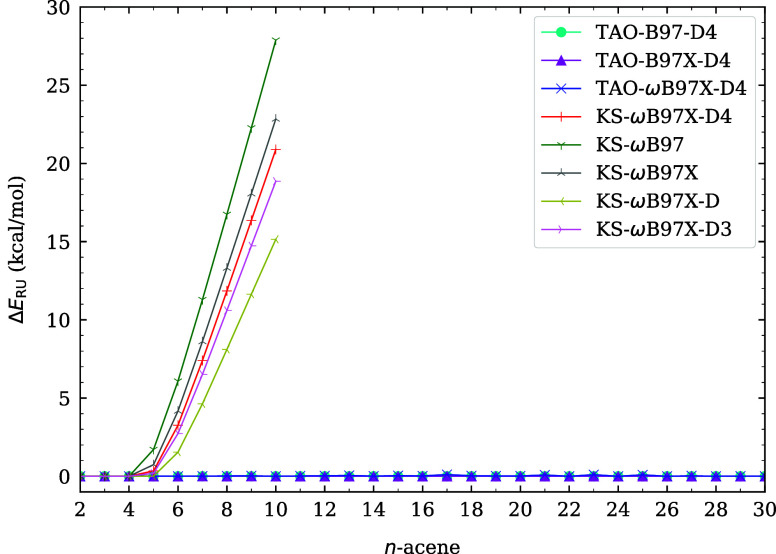
RU energy difference for the lowest singlet state of *n*-acene, obtained with TAO-B97-D4, TAO-B97X-D4, TAO-ωB97X-D4, and KS-ωB97X-D4. For comparison, the results of KS-ωB97, KS-ωB97X, KS-ωB97X-D, and KS-ωB97X-D3 are also included.

At the GS geometry of *n*-acene (with *N* electrons), we compute the vertical ionization potential (IP_v_ = *E*_*N*–1_ – *E*_*N*_), vertical electron affinity (EA_v_ = *E*_*N*_ – *E*_*N*+1_), and fundamental gap (*E*_g_ = IP_v_ – EA_*v*_) for the GS (i.e., the lowest singlet state) of *n*-acene, with *E*_*N*_ being the total energy of the *N*-electron system. As demonstrated in Figure 5, for each TAO-DFT functional, with the increase of acene size, IP_v_ decreases monotonically, EA_v_ increases monotonically, and hence, *E*_g_ decreases monotonically. Besides, the IP_v_, EA_v_, and *E*_g_ values obtained with TAO-B97-D4, TAO-B97X-D4, and TAO-ωB97X-D4 are in good agreement with the experimental data^208^ as well as the data^209,210^ obtained with the CCSD­(T) theory^199^ at the complete basis set (CBS) limit. Similar to our previous findings,^114,115^ *E*_g_ is very insensitive to the choice of TAO-DFT functionals. Relative to the previous results of TAO-DFAs and TAO-GHs,^114,115,124^ notable improvements in the IP_v_ values are achieved with TAO-B97-D4, TAO-B97X-D4, and TAO-ωB97X-D4.

**Figure 5:**
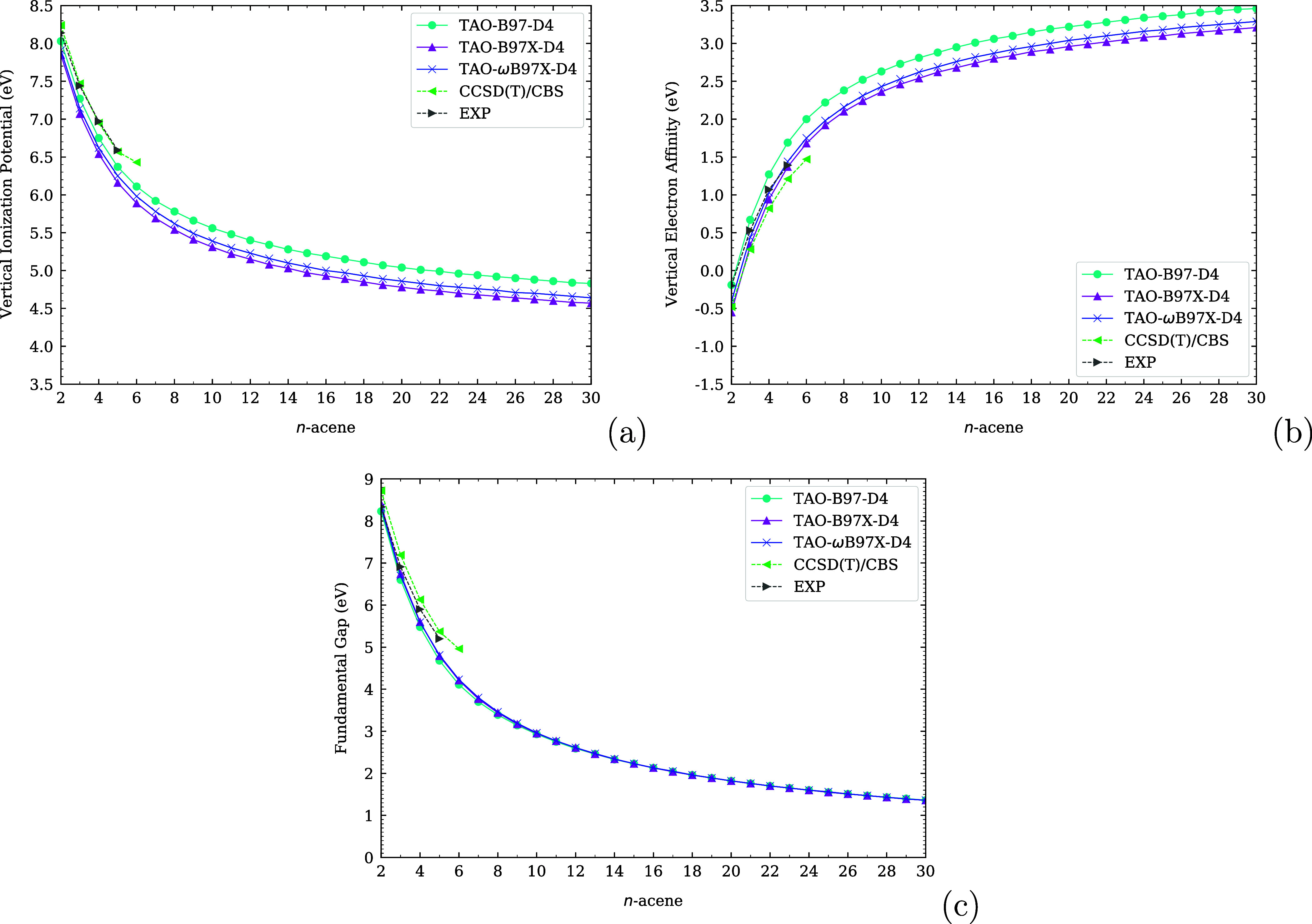
(a) Vertical ionization potential, (b) vertical electron affinity, and (c) fundamental gap for the lowest singlet state of *n*-acene, obtained with spin-unrestricted TAO-B97-D4, TAO-B97X-D4, and TAO-ωB97X-D4. For comparison, the experimental (EXP) data are taken from the work of Malloci et al.,^208^ and the CCSD­(T)/CBS data are taken from the works of Deleuze et al.^209^ and Hajgatoá et al.^210^

As the TOONs can be regarded as the approximate NOONs,^112–115^ the radical nature of a singlet GS system can be assessed by the symmetrized von Neumann entropy^114,115,154,211^

$$S_{\mathrm{vN}}=-\sum_{i=1}^{\infty }\left\{\frac{f_{i}}{2}\ln\left(\frac{f_{i}}{2}\right)+\left(1-\frac{f_{i}}{2}\right)\ln\left(1-\frac{f_{i}}{2}\right)\right\}$$ 89

where *f*_*i*_ (i.e., a number between 0 and 2) is the occupation number of the *i*-th TAO orbital of the singlet GS system, obtained with spin-restricted TAO-DFT. For a singlet GS system with strong nonradical nature, all the TOONs should be in the vicinity of either 0 (fully empty) or 2 (fully occupied), yielding a vanishingly small *S*_vN_ value. However, for a singlet GS system with pronounced radical nature, some of the TOONs can differ greatly from 0 and 2 (e.g., between 0.2 and 1.8), and hence, the *S*_vN_ value can be very large. As presented in Figure 6, the *S*_vN_ value for the lowest singlet state (i.e., the GS) of *n*-acene, obtained with each TAO-DFT functional, monotonically increases with *n*. This implies that the radical nature of GS *n*-acene should increase with the acene length.

**Figure 6:**
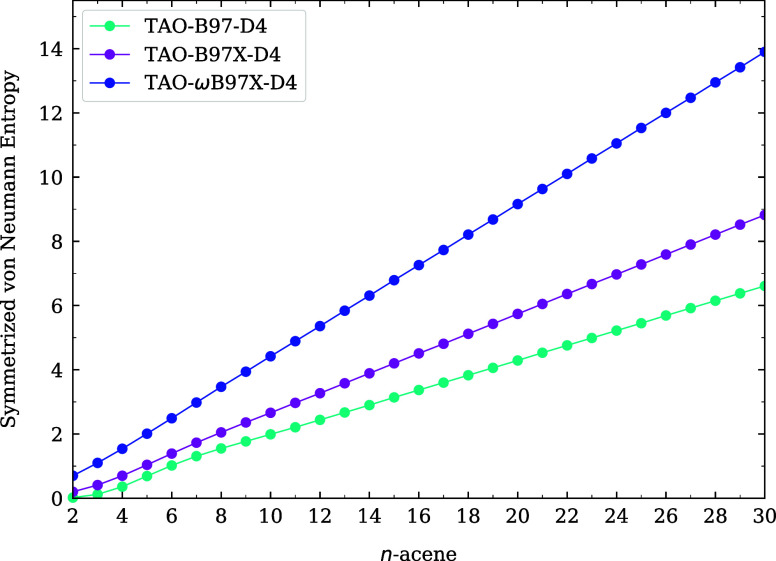
Symmetrized von Neumann entropy for the lowest singlet state of *n*-acene, obtained with spin-restricted TAO-B97-D4, TAO-B97X-D4, and TAO-ωB97X-D4.

To illustrate the reasons why the *S*_vN_ value increases with the acene length, we plot the active TOONs for the lowest singlet state of *n*-acene, calculated using spin-restricted TAO-DFT. Here, the highest occupied molecular orbital (HOMO) is the (*N*/2)-th TAO orbital, the lowest unoccupied molecular orbital (LUMO) is the (*N*/2 + 1)-th TAO orbital, and so forth. As shown in Figure 7, with the increase of acene length, there are more and more active TAO orbitals (e.g., the TAO orbitals with occupation numbers between 0.2 and 1.8) and/or the occupation numbers of active TAO orbitals are closer to 1 (singly occupied), indicating that the larger *n*-acenes should have increasing polyradical nature in their ground states.^96,101,112,115,124,151,211^ Similar to our previous TAO-DFA and TAO-GH studies,^112,115,124^ the active TOONs of TAO-B97-D4 and TAO-B97X-D4 also display a curve crossing behavior in the approach to 1 (singly occupied) with increasing acene length, wherein the TAO orbital with the HOMO (LUMO) character in the smaller *n*-acenes can become the LUMO (HOMO) in the larger *n*-acenes. While the curve crossing behavior of TOONs has been recently supported by the NOONs of v2RDM-CASSCF,^101^ whether such a curve crossing behavior occurs for the larger *n*-acenes remains controversial.^96,102^ On the other hand, the active TOONs of TAO-ωB97X-D4 do not display a curve crossing behavior for *n* ≤ 30, similar to the NOONs of DMRG^96^ and CCVB-SD^102^ methods.

**Figure 7:**
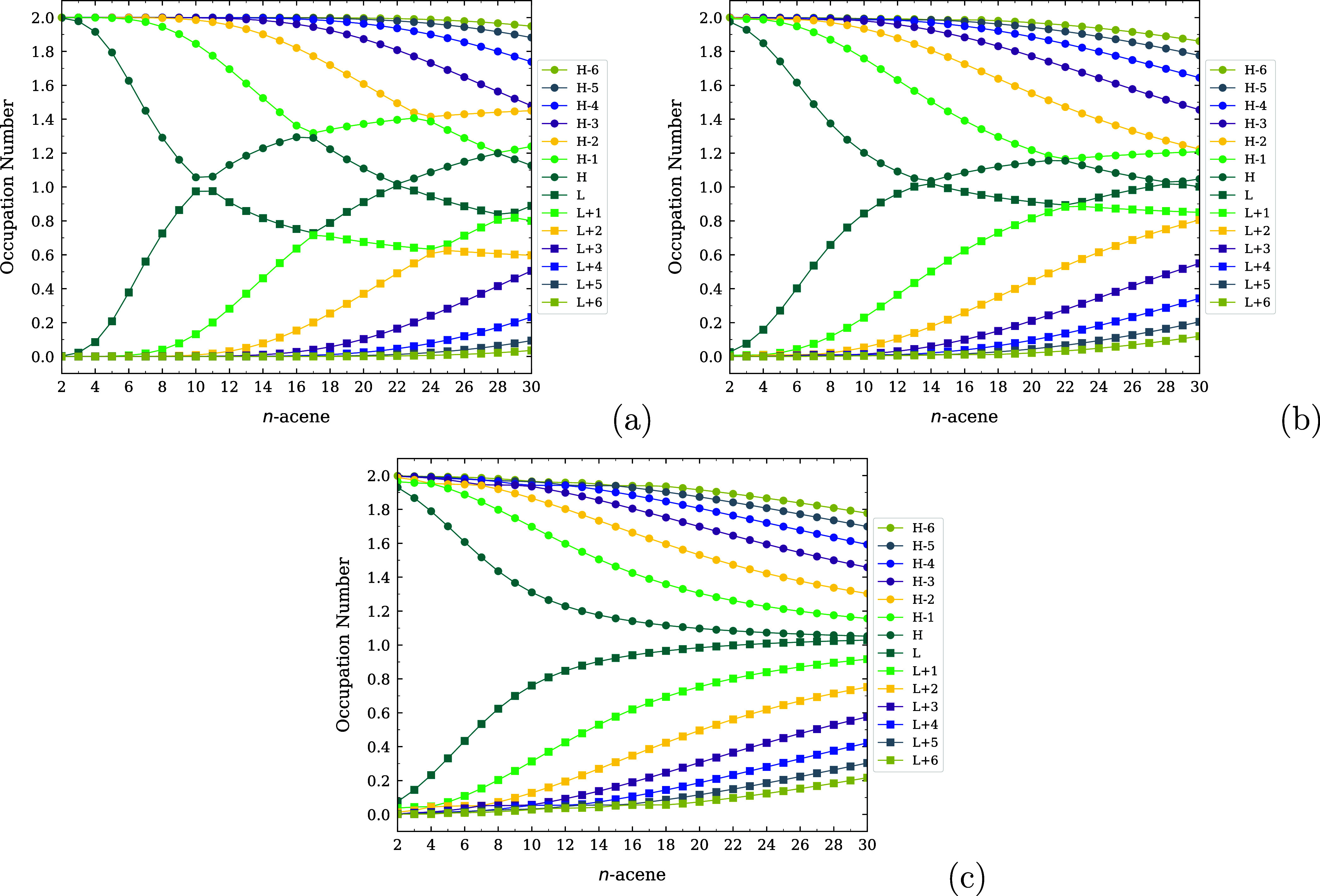
Active TAO orbital occupation numbers (HOMO–6, ..., HOMO–1, HOMO, LUMO, LUMO+1, ..., and LUMO+6) for the lowest singlet state of *n*-acene, obtained with spin-restricted (a) TAO-B97-D4, (b) TAO-B97X-D4, and (c) TAO-ωB97X-D4. For brevity, the HOMO/LUMO is denoted as the H/L.

#### 6.2.2 C_40_ Fullerene Isomers

Recently, Karton developed the iso-C_40_ database,^214^ consisting of the isomerization energies of 29 C_40_ fullerene isomers in the lowest singlet states. In the iso-C_40_ database, each isomer is assigned a serial number based on its lowest singlet-state energy, computed using the high-level G4­(MP2) composite ab initio method (as the approximation of CCSD­(T)/TZ theory)^215–217^ at the KS-PBE-D3/def2-TZVPP optimized geometry. In particular, the electronic isomerization energy (Δ*E*_e,iso_) of an isomer is defined as the CCSD­(T)/TZ energy of the isomer relative to that of isomer No. 1 (e.g., see Table 1 of the iso-C_40_ paper^214^). Using the CCSD­(T)/TZ electronic isomerization energies Δ*E*_e,iso_ as the benchmark values, Karton evaluated the performance of various KS-DFT functionals. The electronic isomerization energies obtained with KS-GHs were found to deviate dramatically from the benchmark values, with even larger discrepancies being observed for KS-RSHs (with 100% LR-HF exchange). Therefore, it was concluded that the inclusion of nonlocal HF exchange in KS-DFAs can deteriorate their performance on the iso-C_40_ database.^214^

We perform spin-unrestricted calculations to examine the performance of TAO-B97-D4, TAO-B97X-D4, TAO-ωB97X-D4, and KS-ωB97X-D4 on the iso-C_40_ database,^214^ using the 6-31G­(d) basis set and EML­(75,302) grid. The isomer geometries and the CCSD­(T)/TZ electronic isomerization energies are taken from the iso-C_40_ database.^214^ For comparison, the results of other KS-DFT and TAO-DFT functionals are also included.

As shown in Table 12 (also see the SI for more detailed results), the KS-RSH functionals (with 100% LR-HF exchange), including KS-ωB97X-D4, KS-ωB97X-D,^56^ KS-M11,^60^ and KS-LC-ωPBE,^50^ perform very poorly, as concluded by Karton.^214^ By contrast, the TAO-GH functionals (with 20–30% HF exchange) consistently outperform the TAO-DFA functionals, suggesting that the inclusion of a suitable amount of HF exchange in the TAO-DFA functionals can greatly improve their performance on the iso-C_40_ database. In particular, TAO-B97X-D4 and TAO-ωB97X-D4 greatly outperform TAO-B97-D4 on the iso-C_40_ database, also highlighting the significance of nonlocal HF exchange in TAO-DFT. On the other hand, the inclusion of dispersion corrections in the TAO-DFT functionals is found to have insignificant effect on their performance for the iso-C_40_ database.

**Table 12:** Statistical Errors (in kJ/mol) of the Electronic Isomerization Energies in the Iso-C_40_ Database^214^

	MSE	MAE	RMS
TAO-LDA	–37.0	37.0	40.1
TAO-BLYP	–27.8	27.8	31.0
TAO-BLYP-D3(0)	–29.4	29.4	32.7
TAO-BLYP-D3(BJ)	–28.1	28.1	31.9
TAO-BLYP-D4	–35.7	35.7	39.2
TAO-PBE	–33.5	33.5	36.6
TAO-PBE-D3(0)	–33.3	33.3	36.5
TAO-PBE-D3(BJ)	–33.2	33.2	36.5
TAO-PBE-D4	–37.1	37.1	40.3
TAO-B97-D4^a^	–39.0	39.0	42.5
TAO-B3LYP	–2.3	11.7	14.2
TAO-B3LYP-D3(0)	–3.4	11.6	14.1
TAO-B3LYP-D3(BJ)	–2.5	11.5	14.0
TAO-B3LYP-D4	–8.1	12.5	15.1
TAO-PBE0	–2.5	12.1	14.8
TAO-PBE0-D3(0)	–2.5	12.1	14.7
TAO-PBE0-D3(BJ)	–2.1	11.9	14.6
TAO-PBE0-D4	–5.3	12.3	14.8
TAO-BHHLYP	26.6	28.5	34.7
TAO-BHHLYP-D3(0)	26.1	27.9	34.0
TAO-BHHLYP-D3(BJ)	26.7	28.5	34.4
TAO-BHHLYP-D4	22.4	24.3	30.1
TAO-B97X-D4^a^	4.6	12.0	15.9
TAO-ωB97X-D4^a^	16.6	18.5	23.3
KS-ωB97X-D4^a^	60.9	60.9	67.5
KS-ωB97X-D^b^			69.3
KS-M11^b^			84.7
KS-LC-ωPBE^b^			103.0

a Resulting functionals in this work.

b Data taken from Karton’s work.^214^

The possible spin-symmetry breaking^10–12,112,114,115,149,211^ is examined by the RU energy difference Δ*E*_RU_ (see eq 88) of each C_40_ fullerene isomer. As shown in Figure 8, the RU energy difference Δ*E*_RU_ of each C_40_ fullerene isomer, obtained with each TAO-DFT functional, remains very small (with the exception of TAO-BHHLYP, where Δ*E*_RU_ = 17 kJ/mol is found for isomer No. 29), implying that essentially no unphysical spin-symmetry breaking effects occur in our spin-unrestricted TAO-DFT solutions (with the exception of TAO-BHHLYP for some isomers). It appears that the optimal system-independent θ (given by eq 55) of TAO-BHHLYP is not sufficiently large^149^ for some isomers (e.g., isomers No. 12, 28, and 29). By contrast, the RU energy difference Δ*E*_RU_ of each C_40_ fullerene isomer, obtained with KS-ωB97X-D4 can be dramatically large (e.g., Δ*E*_RU_ = 93 kJ/mol is found for isomer No. 29), leading to the unphysical spin-symmetry breaking effects in spin-unrestricted KS-ωB97X-D4 solutions for almost all the 29 isomers.

**Figure 8:**
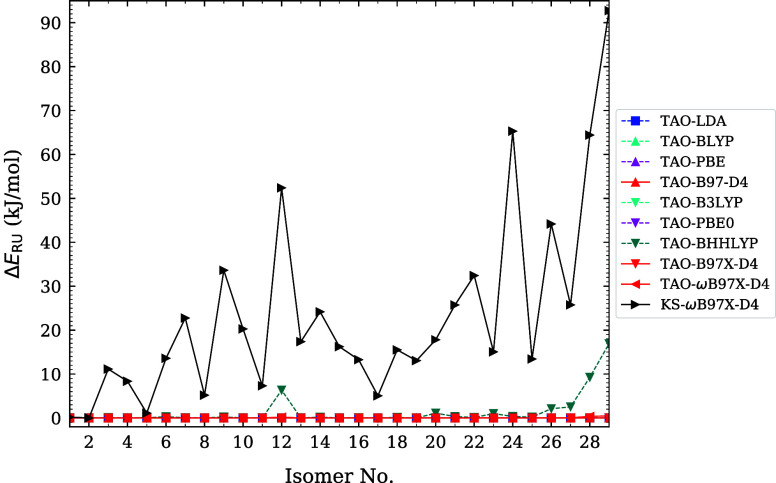
RU energy difference for the lowest singlet state of each C_40_ fullerene isomer (No. 1–29) in the iso-C_40_ database,^214^ obtained with TAO-B97-D4, TAO-B97X-D4, TAO-ωB97X-D4, and KS-ωB97X-D4. For comparison, the results of other TAO-DFT functionals are also included.

The radical nature of each C_40_ fullerene isomer is assessed by the symmetrized von Neumann entropy *S*_vN_ (see eq 89) and active TOONs, obtained with spin-restricted TAO-DFT calculations. As presented in Figure 9, the *S*_vN_ values of all the 29 C_40_ fullerene isomers, obtained with each TAO-DFT functional, are noticeably large, implying that all the 29 C_40_ fullerene isomers should have noticeable radical nature in the lowest singlet states. Besides, as demonstrated in Figure 10, for each TAO-DFT functional, some of the TOONs of each C_40_ fullerene isomer are between 0.2 and 1.8, clearly indicating that all the 29 C_40_ fullerene isomers should have noticeable radical nature in the lowest singlet states.

**Figure 9:**
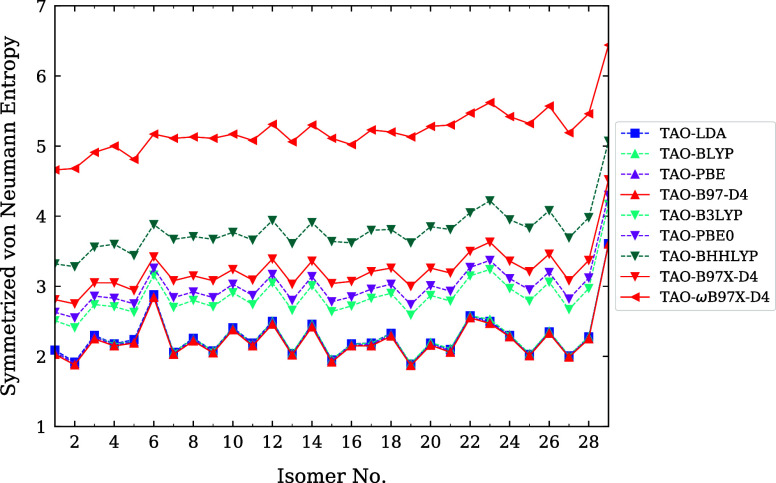
Symmetrized von Neumann entropy for the lowest singlet state of each C_40_ fullerene isomer (No. 1–29) in the iso-C_40_ database,^214^ obtained with spin-restricted TAO-B97-D4, TAO-B97X-D4, and TAO-ωB97X-D4. For comparison, the results of other TAO-DFT functionals are also included.

**Figure 10:**
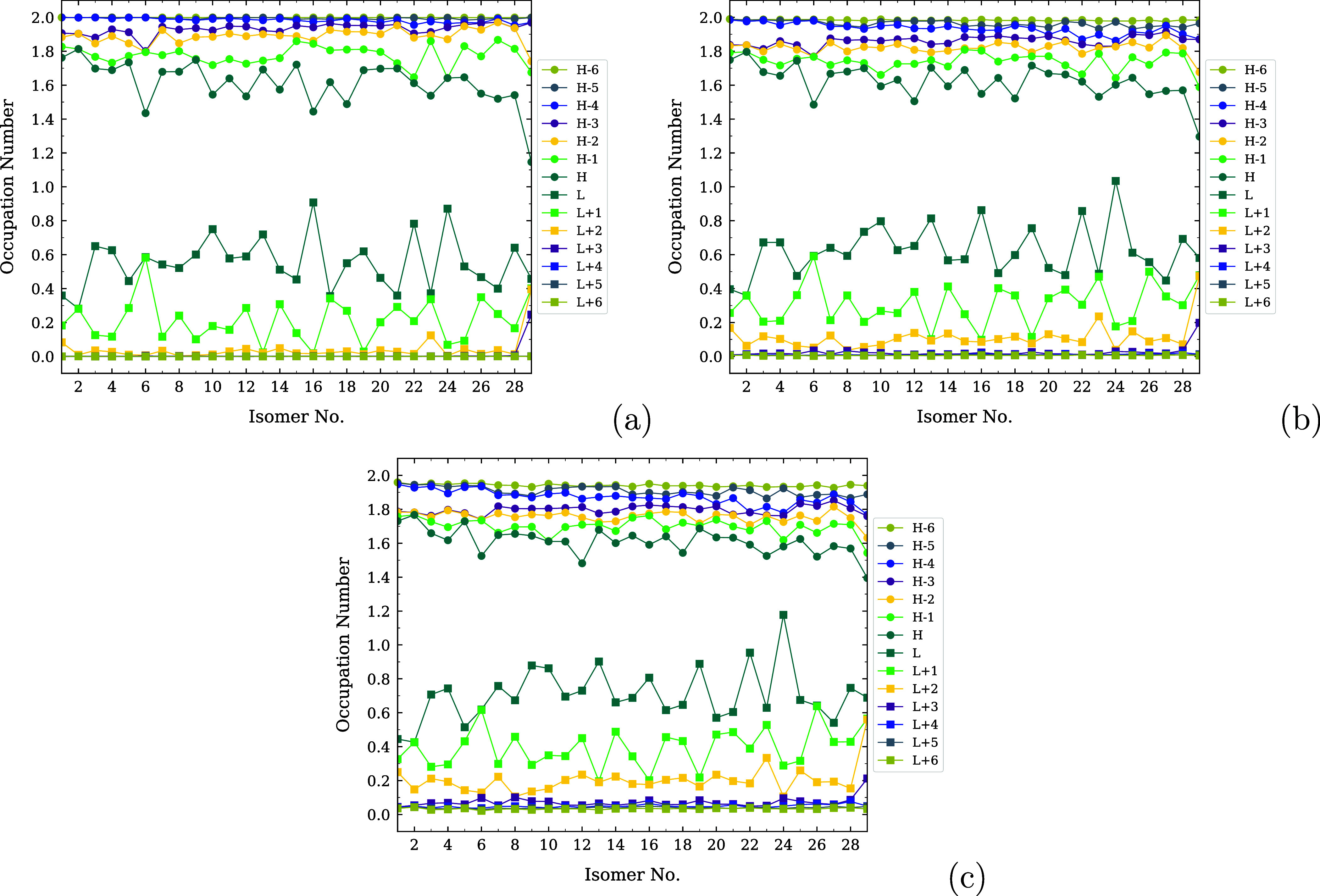
Active TAO orbital occupation numbers (HOMO–6, ..., HOMO–1, HOMO, LUMO, LUMO+1, ..., and LUMO+6) for the lowest singlet state of each C_40_ fullerene isomer (No. 1–29) in the iso-C_40_ database,^214^ obtained with spin-restricted (a) TAO-B97-D4, (b) TAO-B97X-D4, and (c) TAO-ωB97X-D4. For brevity, the HOMO/LUMO is denoted as the H/L.

In short, based on the RU energy difference Δ*E*_RU_, symmetrized von Neumann entropy *S*_vN_, and active TOONs, all the 29 C_40_ fullerene isomers of the iso-C_40_ database,^214^ should possess noticeable radical nature in the lowest singlet states. Therefore, KS-ωB97X-D4 and other KS-RSHs can perform very poorly on the iso-C_40_ database, while TAO-B97X-D4 and TAO-ωB97X-D4 perform reasonably well on the iso-C_40_ database, showing the significance of TAO-GHs and TAO-RSHs.

#### 6.2.3 Dissociation of H_2_ and N_2_

Because of the spin-symmetry constraint,^10–12,112,114,115,149,211^ the spin-restricted and spin-unrestricted potential energy curve of H_2_ (i.e., a single-bond breaking system), computed using the exact theory, should be the same. Therefore, for the exact theory, the RU energy difference Δ*E*_RU_ must be zero for each internuclear separation. KS-DFT with the conventional xc energy functionals, such as KS-DFAs, KS-GHs, and KS-RSHs, can perform reasonably well in a region dominated by a single-reference character (e.g., near the equilibrium geometry of H_2_), while yielding a significantly large error in a region dominated by a multireference character (e.g., at the dissociation limit of H_2_), yielding a significantly large Δ*E*_RU_ at the dissociation limit (often referred to as the SCE).^10,11,33,34,112,114,115^ Similar situation occurs for N_2_ dissociation (i.e., a triple-bond breaking system).

To examine the performance of TAO-B97-D4, TAO-B97X-D4, and TAO-ωB97X-D4 upon the SCE problems, spin-restricted calculations (with various θ values) are performed for the dissociation energy curves of H_2_ and N_2_, using the 6-311++G­(3df,3pd) basis set and EML­(75,302) grid. For H_2_ dissociation, the exact curve obtained with the CCSD theory (i.e., the coupled-cluster theory with iterative singles and doubles)^218^ is included for comparison.

As shown in Figure 11 (for H_2_ dissociation) and Figure 12 (for N_2_ dissociation), TAO-B97-D4 (with θ = 0), TAO-B97X-D4 (with θ = 0), and TAO-ωB97X-D4 (with θ = 0), which, as noted earlier, are the KS-DFA, KS-GH, and KS-RSH functionals, respectively, yield significantly large SCEs. In general, the larger the fraction of HF exchange in the KS-DFT functional, the larger the SCE of the KS-DFT functional. Accordingly, for the dissociation of H_2_ and N_2_, TAO-ωB97X-D4 (with θ = 0) yields much larger SCE than TAO-B97-D4 (with θ = 0). With the optimal system-independent θ values (see Table 3), TAO-B97-D4 (with θ = 9.59717 mhartree), TAO-B97X-D4 (with θ = 24.0964 mhartree), and TAO-ωB97X-D4 (with θ = 50.2796 mhartree) consistently improve upon their KS-DFT counterparts (i.e., the θ = 0 cases), while the corresponding SCEs remain large.

**Figure 11:**
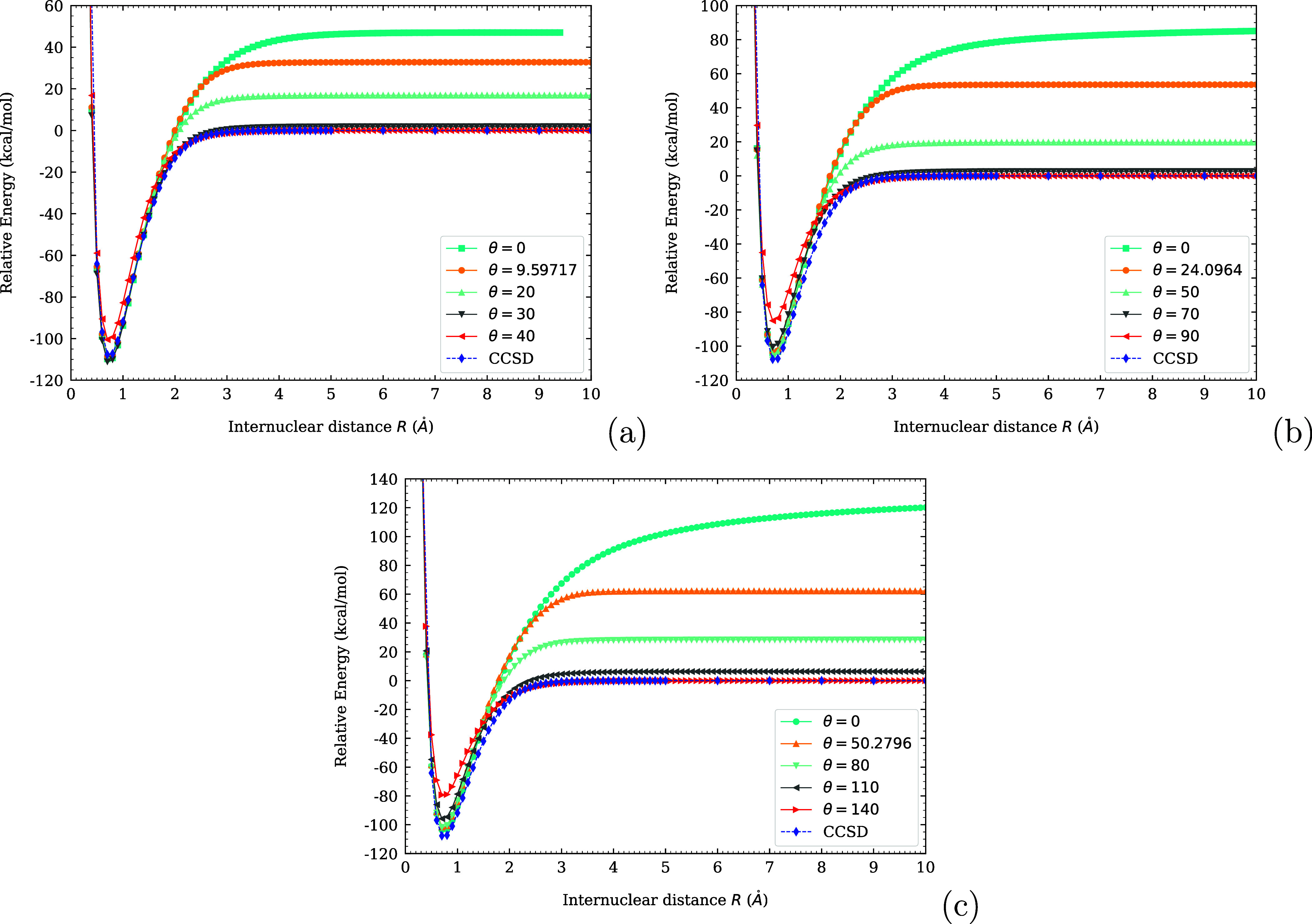
Spin-restricted potential energy curve (in relative energy) for the lowest singlet state of H_2_, computed using (a) TAO-B97-D4, (b) TAO-B97X-D4, and (c) TAO-ωB97X-D4, with various θ (in mhartree). The zero of energy is set at the respective spin-unrestricted dissociation limit. For comparison, the exact curve, obtained with the CCSD theory,^218^ is also included.

**Figure 12:**
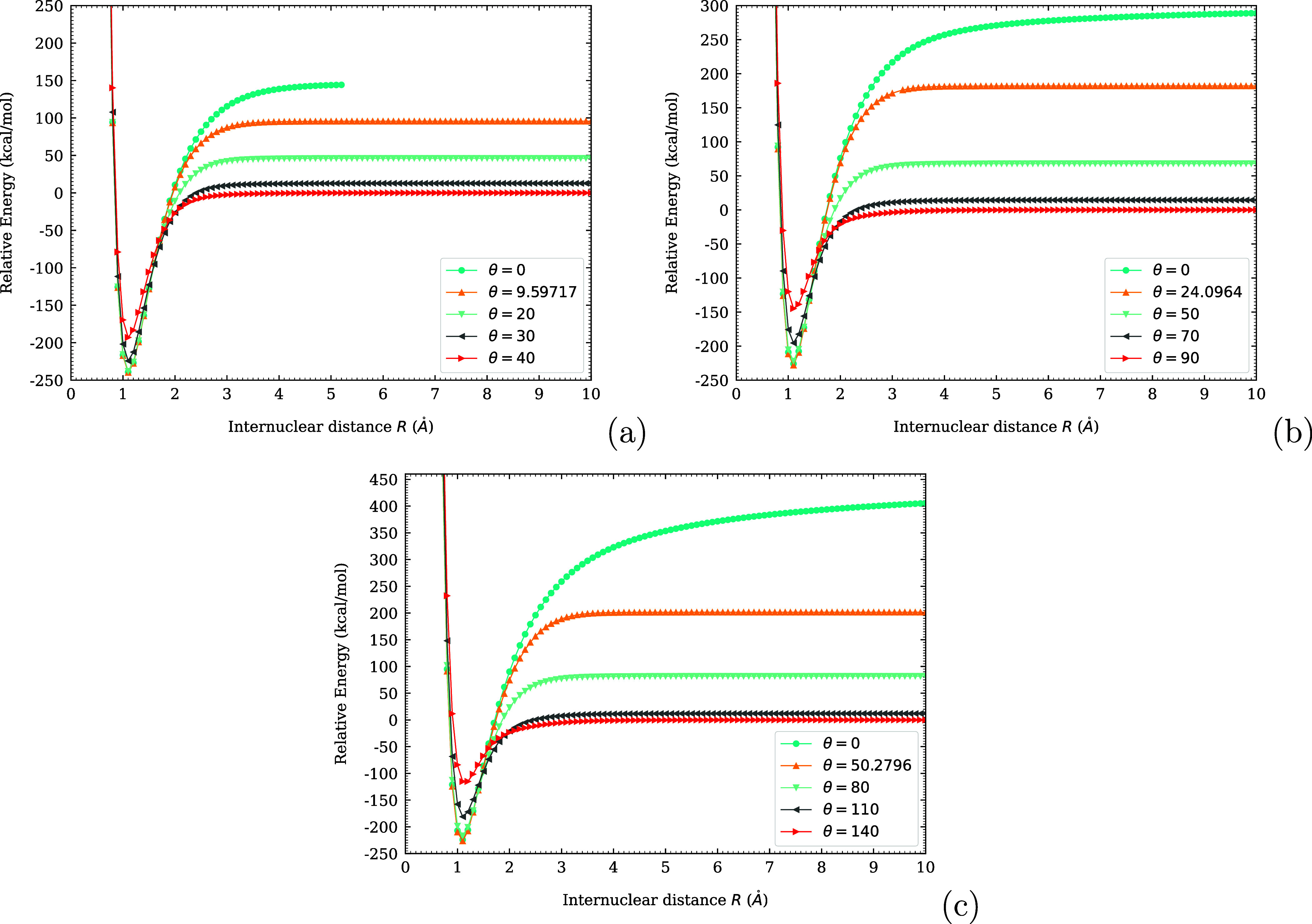
Spin-restricted potential energy curve (in relative energy) for the lowest singlet state of N_2_, computed using (a) TAO-B97-D4, (b) TAO-B97X-D4, and (c) TAO-ωB97X-D4, with various θ (in mhartree). The zero of energy is set at the respective spin-unrestricted dissociation limit.

According to our recent work,^149^ for H_2_ and N_2_ dissociations (i.e., singlet GS systems), TAO-DFT with a sufficiently large θ can always resolve the SCE problems. As aforementioned, the optimal θ values for the dissociation of H_2_ and N_2_ can be defined as the minimum θ values with which the spin-restricted and spin-unrestricted solutions obtained from the same TAO-DFT functionals are identical at the dissociation limits of H_2_ and N_2_, which are approximately 40 mhartree for TAO-B97-D4, 90 mhartree for TAO-B97X-D4, and 140 mhartree for TAO-ωB97X-D4. However, the optimal θ values for the dissociation of H_2_ and N_2_, are approximately three to four times larger than the corresponding optimal system-independent θ values, which are too large for the equilibrium geometries of H_2_ and N_2_, where the ground states of the molecules possess single-reference character. Therefore, for the TAO-DFT functionals, this goes beyond the limitations of the system-independent θ scheme (given by eq 55) adopted in this work, suggesting that an optimal system-dependent θ scheme is needed.

#### 6.2.4 Long-Range Charge-Transfer Excitations

In a neutral system consisting of two well-separated molecules, a long-range charge-transfer (CT) excitation involves the transfer of an electron from one molecule (the donor) to the other molecule (the acceptor).^219^ For a sufficiently large intermolecular distance *R*, the lowest CT excitation energy should have the following asymptote:^9,55,219–221^

$$\omega_{\mathrm{CT}}(R\to \infty )\approx -\frac{1}{R}+{\mathrm{IP}}_{D}-{\mathrm{EA}}_{A}$$ 90

where IP_D_ is the ionization potential of the donor, EA_A_ is the electron affinity of the acceptor, and the (−1/*R*) dependence is the consequence of Coulomb interactions, which can be accurately described by the nonlocal HF exchange.^220^

Here we consider the lowest CT excitation energy between two molecules, involving the ethylene (C_2_H_4_) and tetrafluoroethylene (C_2_F_4_) dimer (e.g., see Figure 1 in the work of Dreuw et al.^220^), Ar···S_3_ dimer (see Figure 13a), and S_3_···Ar dimer (see Figure 13b), with a separation of *R*. Among these molecules, the C_2_H_4_ and C_2_F_4_ molecules and the argon (Ar) atom are single-reference systems, while the S_3_ molecule is a multireference system.^222^

**Figure 13:**
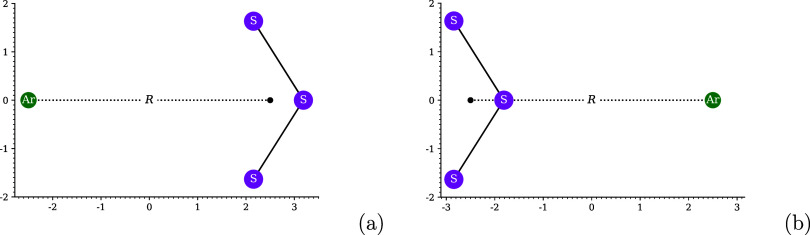
Geometries of (a) Ar···S_3_ and (b) S_3_···Ar dimers. The intermolecular distance *R* is measured from Ar to the center of mass of S_3_.

To examine the possible spin-symmetry breaking effects in the lowest CT excitation energy due to a spin-symmetry-broken GS,^10–12,112,149,211^ we perform both spin-restricted (R) and spin-unrestricted (U) calculations employing the KS/TDA (for each TAO-DFT functional with θ = 0 and for each KS-DFT functional) and pTAO/TDA (for each TAO-DFT functional with the optimal system-independent θ (see Table 3)) methods, within the adiabatic approximations, using the 6-31G­(d) basis set and EML­(99,590) grid, for the lowest CT excitation energy between each dimer (C_2_H_4_···C_2_F_4_ dimer, Ar···S_3_ dimer, and S_3_···Ar dimer) with a separation of *R*.

For each functional, the geometry of each monomer (C_2_H_4_, C_2_F_4_, and S_3_) is optimized using spin-unrestricted GS calculations, and the geometries of each dimer (C_2_H_4_···C_2_F_4_ dimer, Ar···S_3_ dimer, and S_3_···Ar dimer) at different values of *R* are obtained by varying the distance between the centers of mass of the two monomers without further reoptimizations.

As presented in Figure 14 (also see the SI for more detailed results), for the lowest CT excitation energies of C_2_H_4_···C_2_F_4_ dimer, unphysical spin-symmetry breaking effects are not observed in the results of spin-unrestricted KS/TDA and pTAO/TDA for all the functionals considered, which is reasonable given the single-reference character of the two molecules C_2_H_4_ and C_2_F_4_. Besides, TAO-ωB97X-D4, TAO-ωB97X-D4 (with θ = 0), and KS-ωB97X-D4, which contain 100% LR-HF exchange, can capture the long-range Coulomb interaction in the CT excitations, and yield CT excitation curves that are in qualitative agreement with the high-level ab initio results obtained with the symmetry-adapted-cluster configuration interaction (SAC-CI) method,^47^ while the other functionals yield qualitatively incorrect CT excitation curves. Among all the functionals considered, TAO-ωB97X-D4 performs the best, but the predicted CT excitation energies are still in error of 1 eV when compared with the reference data. Therefore, it may be necessary to adopt a more flexible operator^58^ for the HF exchange adopted in the RSH functionals to reduce such errors.

**Figure 14:**
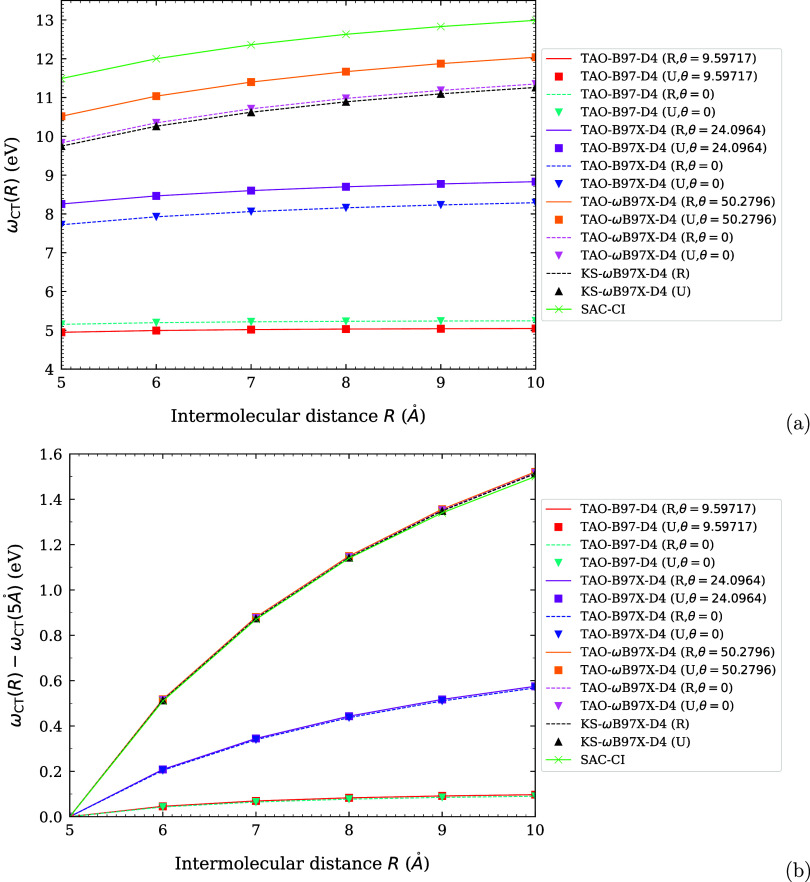
(a) The lowest CT excitation energy of C_2_H_4_···C_2_F_4_ dimer along the intermolecular distance *R* (in Å), obtained with both spin-restricted (R) and spin-unrestricted (U) calculations employing the KS/TDA (for each TAO-DFT functional with θ = 0 and for each KS-DFT functional) and pTAO/TDA (for each TAO-DFT functional with the optimal system-independent θ (in mhartree), see Table 3) methods. For comparison, the SAC-CI data are taken from the work of Tawada et al.^47^ (b) Same as (a), but in relative excitation energy, where the excitation energy at 5 Å is set to zero for each method.

As shown in Figures 15 and 16, for the lowest CT excitation energies of the Ar···S_3_ and S_3_···Ar dimers (i.e., multireference systems), unphysical spin-symmetry breaking effects are observed in the results of spin-unrestricted KS/TDA using KS-ωB97X-D4 and TAO-ωB97X-D4 (with θ = 0). Such an unphysical spin-symmetry breaking feature of spin-unrestricted KS/TDA using KS-ωB97X-D4 and TAO-ωB97X-D4 (with θ = 0) is apparently undesirable for ES calculations. By contrast, unphysical spin-symmetry breaking effects are not observed in the results of spin-unrestricted pTAO/TDA for all the TAO-DFT functionals considered, which can be attributed to the satisfaction of spin-symmetry constraint on the singlet GS densities of the Ar···S_3_ and S_3_···Ar dimers by spin-unrestricted TAO-B97-D4, TAO-B97X-D4, and TAO-ωB97X-D4. It is worth mentioning that for the Ar···S_3_ and S_3_···Ar dimers, TAO-ωB97X-D4 predicts the lowest CT excitation energy curve with the correct asymptotic behavior of (−1/*R*) without unphysical spin-symmetry breaking effects, highlighting the significance of the pTAO/TDA method adopting the TAO-RSH functional with 100% LR-HF exchange.

**Figure 15:**
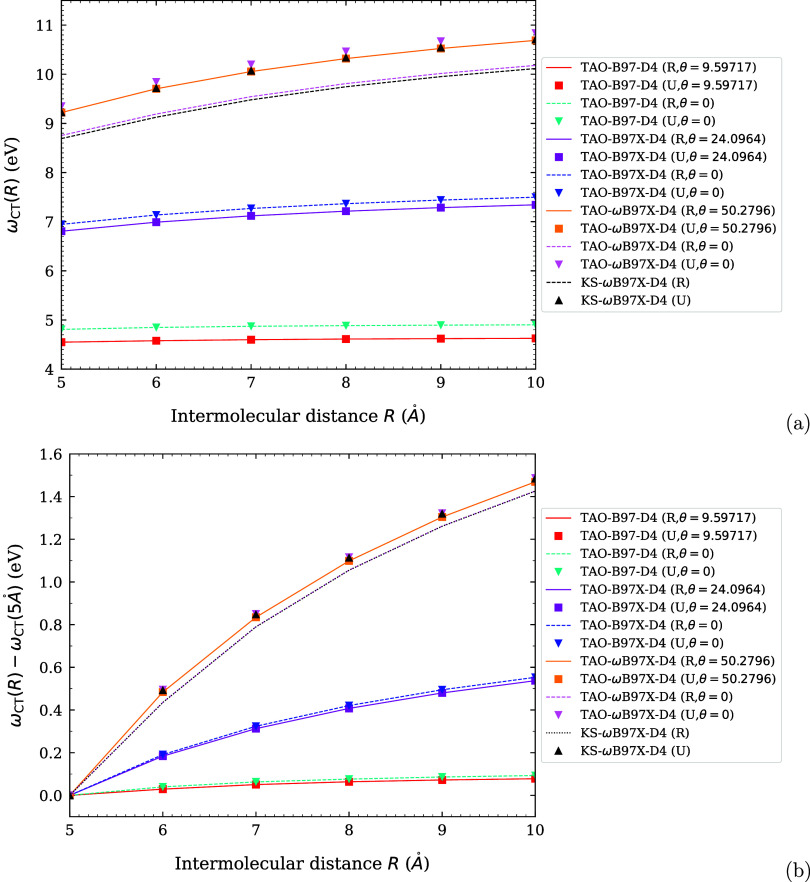
(a) The lowest CT excitation energy of Ar···S_3_ dimer along the intermolecular distance *R* (in Å), obtained with both spin-restricted (R) and spin-unrestricted (U) calculations employing the KS/TDA (for each TAO-DFT functional with θ = 0 and for each KS-DFT functional) and pTAO/TDA (for each TAO-DFT functional with the optimal system-independent θ (in mhartree), see Table 3) methods. (b) Same as (a), but in relative excitation energy, where the excitation energy at 5 Å is set to zero for each method.

**Figure 16:**
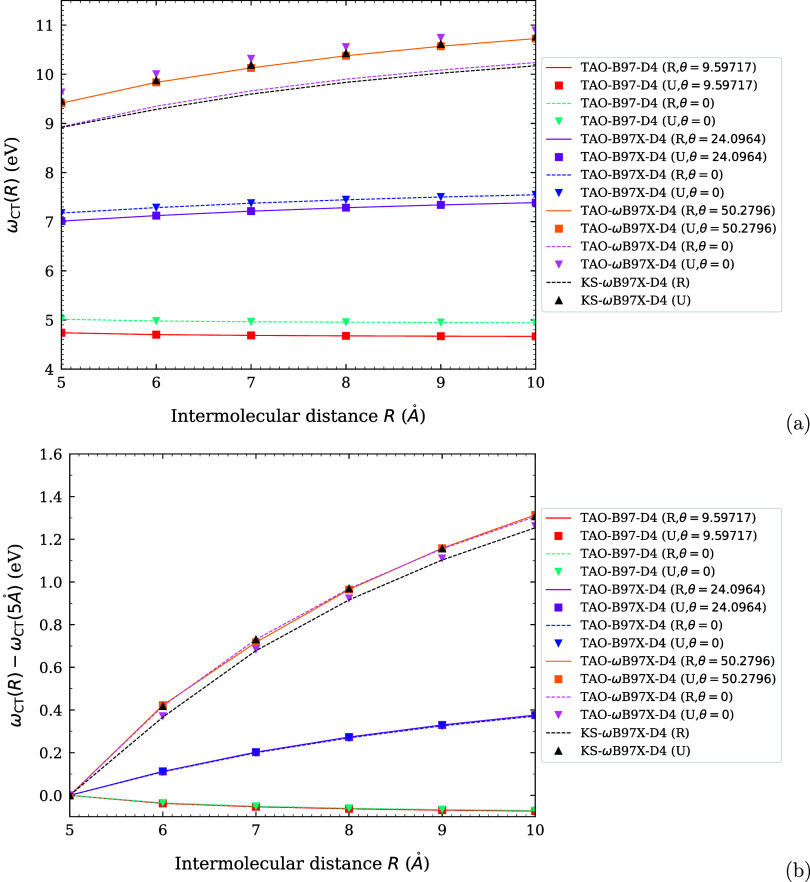
(a) The lowest CT excitation energy of S_3_···Ar dimer along the intermolecular distance *R* (in Å), obtained with both spin-restricted (R) and spin-unrestricted (U) calculations employing the KS/TDA (for each TAO-DFT functional with θ = 0 and for each KS-DFT functional) and pTAO/TDA (for each TAO-DFT functional with the optimal system-independent θ (in mhartree), see Table 3) methods. (b) Same as (a), but in relative excitation energy, where the excitation energy at 5 Å is set to zero for each method.

In short, for MR systems, spin-unrestricted KS/TDA can lead to unphysical spin-symmetry breaking effects in the excitation energies, which is apparently undesirable for ES calculations. This issue can be greatly resolved by spin-unrestricted pTAO/TDA with a sufficiently large θ. Besides, to reasonably predict long-range CT excitations between two well-separated molecules, which can possess single-reference or multireference character in their electronic ground states, the pTAO/TDA method with TAO-ωB97X-D4 seems very promising!

## 7 Conclusions

In conclusion, we have proposed an analytical parametrization for the optimal system-independent fictitious temperature θ (see eq 55) of TAO-ωDFAX (i.e., the TAO-RSH functional with 100% LR-HF exchange), as a function of the range-separation parameter ω and the SR-HF exchange fraction *a*_*x*_, based on CC’s θ_A_-model.^151^ Accordingly, the optimal system-independent θ values of TAO-DFA (with ω = 0 and *a*_*x*_ = 0), TAO-GH (with ω = 0), and TAO-RSH functionals can be determined. Adopting this parametrization of system-independent θ, we have developed the reoptimized B97-type TAO-DFA, TAO-GH, and TAO-RSH functionals with the D4 dispersion corrections, denoted as TAO-B97-D4, TAO-B97X-D4, and TAO-ωB97X-D4, respectively. Besides, by enforcing the constraint θ = 0 in parameter optimization, we have also developed the reoptimized B97-type KS-RSH functional with the D4 dispersion corrections, denoted as KS-ωB97X-D4. Moreover, within TAO-DFT, we have proposed the pTAO/TDA method to obtain excitation energies, wherein the adiabatic approximation (i.e., for the sake of computational efficiency) can be adopted without the issues of spurious excitations.^152,153,192^

To comprehensively assess how TAO-B97-D4, TAO-B97X-D4, TAO-ωB97X-D4, and KS-ωB97X-D4 perform outside their training sets, we have also examined their performance on a very wide variety of test sets, including both single-reference systems (e.g., the GMTKN55 database and the equilibrium geometries of EXTS set) and multireference systems (e.g., the iso-C_40_ database and linear acenes). Furthermore, we have also examined their performance on challenging test sets, including the dissociation of H_2_^+^ and He_2_^+^, dissociation of H_2_ and N_2_, and long-range CT excitations in molecules with or without strong static correlation.

Overall, KS-ωB97X-D4 yields high accuracy for the properties (e.g., thermochemistry, kinetics, and noncovalent interactions) of single-reference systems. For example, among all the functional considered, KS-ωB97X-D4 performs comparably to KS-ωB97M-V, and consistently outperforms KS-ωB97X, KS-ωB97X-D3, and KS-ωB97X-V for the entire GMTKN55 database. Notably, KS-ωB97X-D4 performs the best in the categories of noncovalent interactions. For the dissociation of H_2_^+^ and He_2_^+^, KS-ωB97X-D4 yields the qualitatively correct dissociation curves (i.e., with no unphysical barriers). For long-range CT excitations in molecules without strong static correlation, KS-ωB97X-D4 (using the KS/TDA method) can capture the long-range Coulomb interaction in the CT excitations, yielding qualitatively correct CT excitation curves. However, similar to all the KS-DFAs, KS-GHs, and other KS-RSHs, KS-ωB97X-D4 can perform very poorly for multireference systems, including the iso-C_40_ database, linear acenes, the dissociation of H_2_ and N_2_, and long-range CT excitations in molecules with strong static correlation.

On the other hand, TAO-ωB97X-D4 and TAO-B97X-D4 achieve reasonably good performance for both single-reference and multireference systems. For example, TAO-ωB97X-D4 and TAO-B97X-D4 perform reasonably well for the entire GMTKN55 database, showing comparable performance to KS-ωB97X, KS-BHHLYP-D3­(BJ), and KS-PW6B95-D3­(BJ). For multireference systems, such as the iso-C_40_ database and linear acenes, TAO-ωB97X-D4 and TAO-B97X-D4 consistently outperform the KS-DFT functionals. For long-range CT excitations in molecules with or without strong static correlation, with the pTAO/TDA method, TAO-ωB97X-D4 can capture the long-range Coulomb interaction in the CT excitations, and yield qualitatively correct CT excitation curves. Therefore, TAO-ωB97X-D4, which possesses 100% LR-HF exchange, is more desirable than TAO-B97X-D4.

Due to the lack of nonlocal HF exchange, TAO-B97-D4 is less accurate than TAO-ωB97X-D4 and TAO-B97X-D4 for various applications. However, owing to its computational efficiency, TAO-B97-D4 is favorable for exploring the properties of large single-reference and multireference systems, especially for TAO-AIMD simulations.^154^

Certainly, more extensive examinations remain necessary to have a better understanding of the performance of TAO-B97-D4, TAO-B97X-D4, TAO-ωB97X-D4, and KS-ωB97X-D4. Note, however, that there remain some limitations in the present work due to the use of system-independent θ scheme (see eq 55) in TAO-DFT as well as the erfx operator for HF exchange (see eq 31) adopted in the RSH functionals. For example, for each TAO-DFT functional considered, the optimal system-independent θ (see eq 55) is too large for H_2_^+^ and He_2_^+^ dissociations (i.e., single-reference systems), while it is too small for H_2_ and N_2_ dissociations (i.e., multireference systems). This implies that for each TAO-DFT functional, a scheme^150^ determining the optimal θ of an electronic system remains crucial for improved performance on both single-reference and multireference systems. It can be anticipated that developing a computationally efficient and generally accurate scheme for the determination of the optimal system-dependent θ for the TAO-DFA, TAO-GH, and TAO-RSH functionals will be an important object of futures research in TAO-DFT. Besides, the erfx operator for the HF exchange (see eq 31) adopted in the RSH functionals may not be sufficiently flexible for accurately describing the dissociation of H_2_^+^ and He_2_^+^ as well as the long-range CT excitations. This indicates that a more flexible operator^58^ for the HF exchange adopted in the RSH functionals should be further explored to yield better performance. Alternatively, the optimally tuned RSH functionals^223^ can also be considered. According to our findings in this work, it seems likely that the aforementioned three qualitative errors (SIE, NCIE, and SCE) can be adequately addressed by the dispersion-corrected TAO-RSH functionals (with a flexible operator^58^ for the HF exchange) in the optimal system-dependent θ scheme. We plan to work along these lines, and results may be reported elsewhere.

## Supplementary Material



## Data Availability

The data that support the findings of this study are available within the article and its Supporting Information.

## Notes

Detailed KS-DFT and TAO-DFT results for the GMTKN55 database, EXTS set, the NOONs of He_2_^+^ dissociation, electronic properties of linear acenes, iso-C_40_ database, and lowest CT excitation energies of C_2_H_4_···C_2_F_4_ dimer, Ar···S_3_ dimer, and S_3_···Ar dimer (PDF)
