# Supplementary material for: Optimization of B97-Type Density Functional Approximation, Global Hybrid, and Range-Separated Hybrid Energy Functionals with the D4 Dispersion Corrections in TAO-DFT
Source: J Chem Theory Comput. 2025 Sep 29;21(19):9538–72. doi: 10.1021/acs.jctc.5c01037 (PMC12529923; doi:10.1021/acs.jctc.5c01037)
Supplement: Supplementary file 1 [file ct5c01037_si_001.pdf]

Supporting Information: Optimization of B97-Type Density Functional Approximation, Global Hybrid, and Range-Separated Hybrid Energy Functionals with the D4 Dispersion Corrections in TAO-DFT

Shaozhi Li[†] and Jeng-Da Chai[*,†,‡,¶]

†*Department of Physics, National Taiwan University, Taipei 10617, Taiwan*

‡*Center for Theoretical Physics and Center for Quantum Science and Engineering, National Taiwan University, Taipei 10617, Taiwan*

¶*Physics Division, National Center for Theoretical Sciences, Taipei 10617, Taiwan*

E-mail: jdchai@phys.ntu.edu.tw

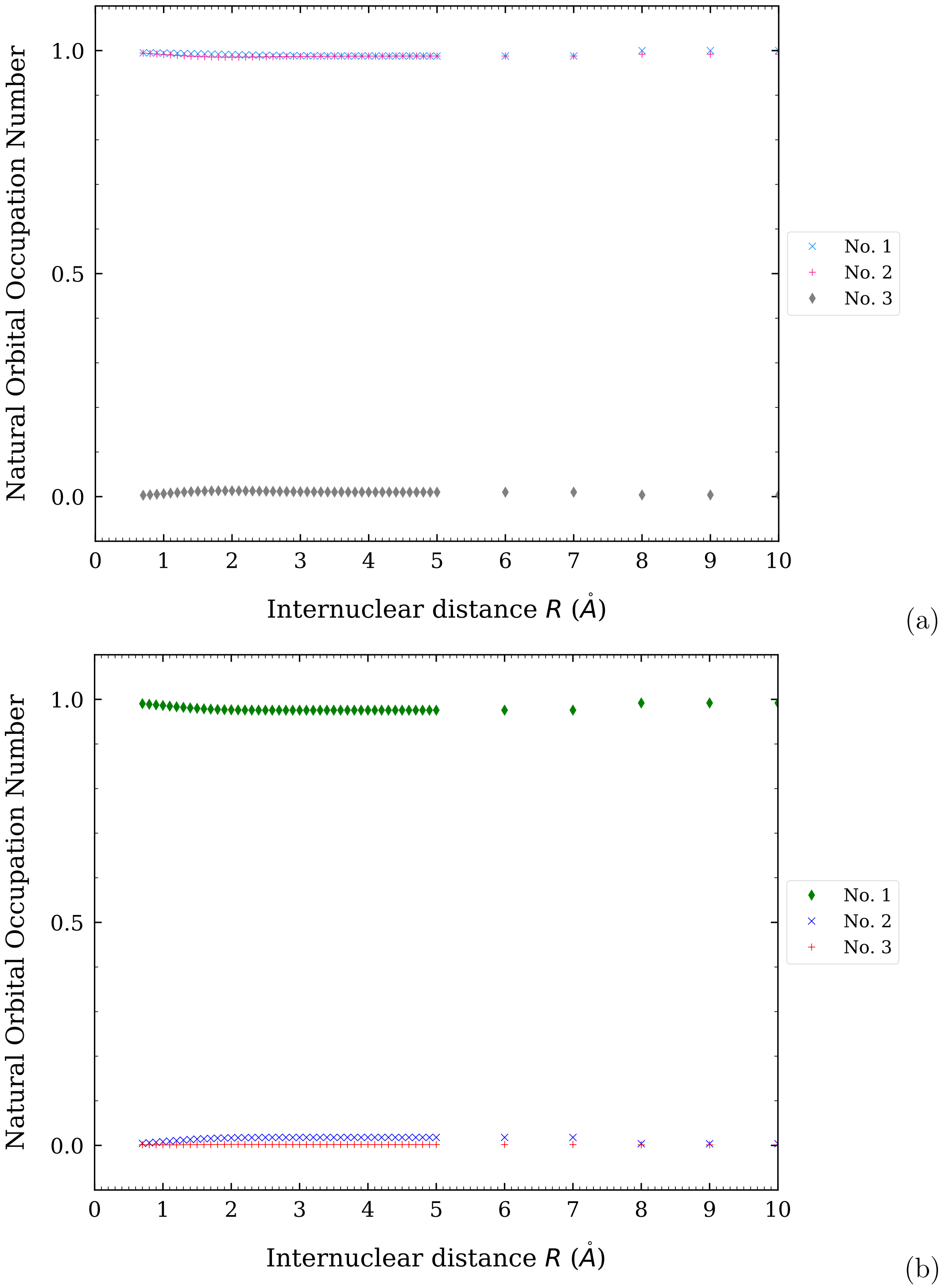

Figure S1: (a) α-spin and (b) β-spin natural orbital occupation numbers for the dissociation curve of He_2^+, obtained with the adaptive sampling configuration interaction (ASCI) method,[1] using the aug-cc-pVQZ basis set. The active space includes all 3 electrons that are allowed to occupy all 92 orbitals, corresponding to the ASCI wavefunction with 385112 determinants. For each spin, the natural orbitals are arranged by their occupation numbers, with the orbitals having higher occupation numbers placed before the orbitals with lower occupation numbers.

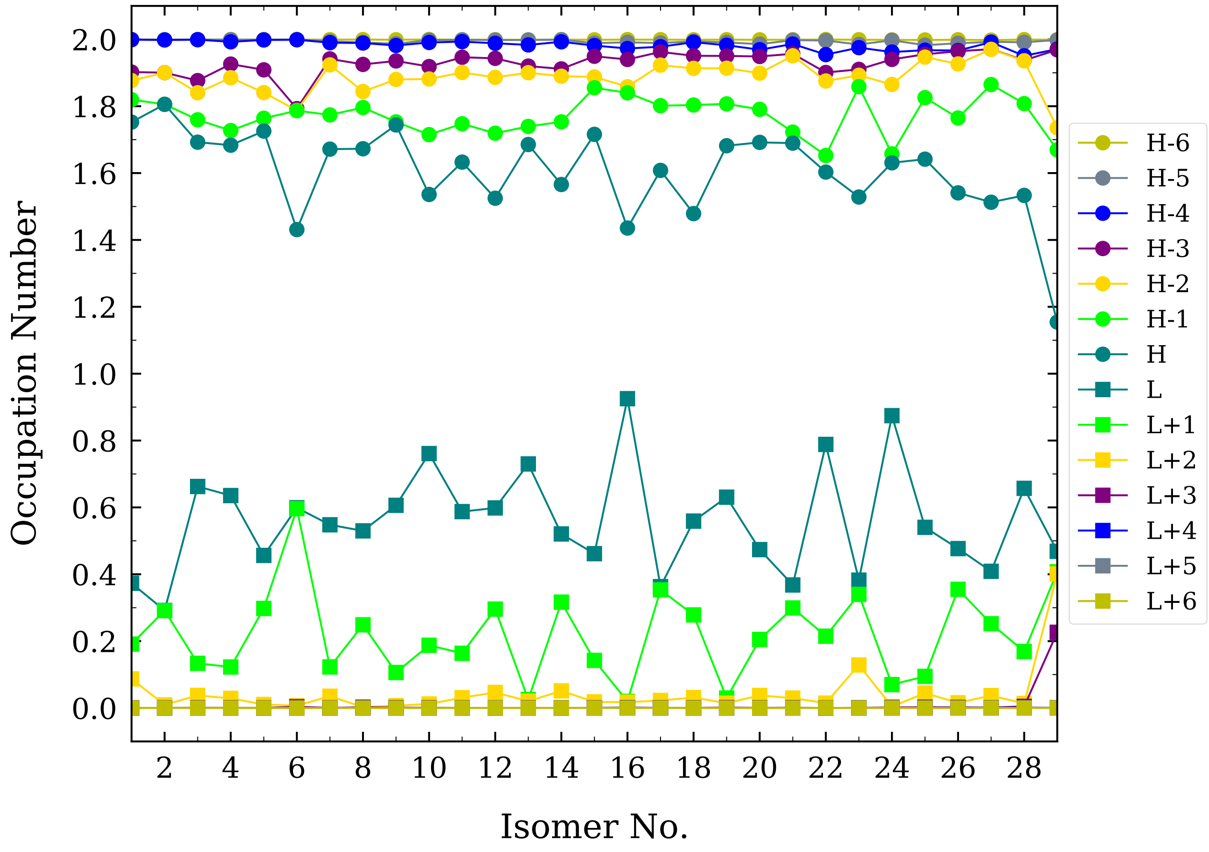

Figure S2: Active TAO orbital occupation numbers (HOMO−6, ..., HOMO−1, HOMO, LUMO, LUMO+1, ..., and LUMO+6) for the lowest singlet state of each C_{40} fullerene isomer (No. 1 to No. 29) in the iso-C_{40} database,[2] obtained with spin-restricted TAO-LDA. For brevity, the HOMO/LUMO is denoted as the H/L.

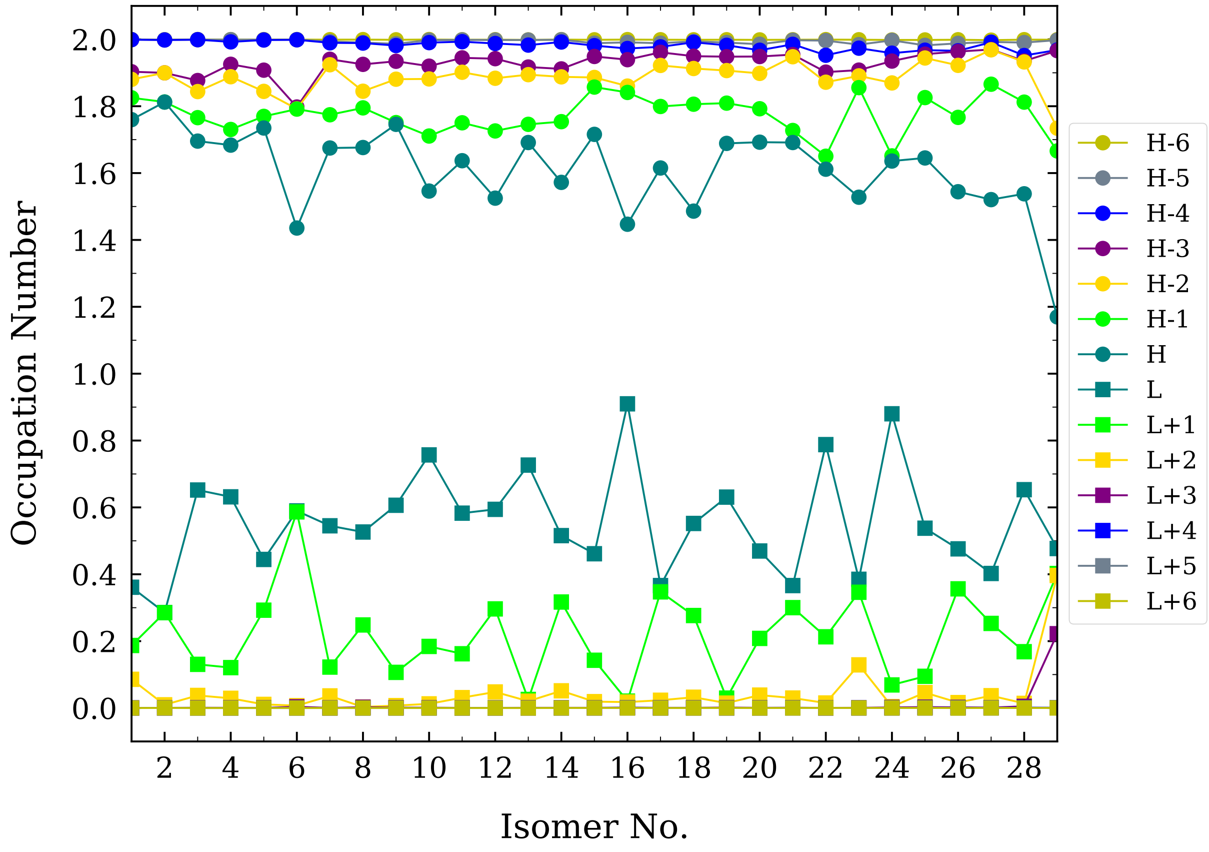

Figure S3: Active TAO orbital occupation numbers (HOMO−6, ..., HOMO−1, HOMO, LUMO, LUMO+1, ..., and LUMO+6) for the lowest singlet state of each C_{40} fullerene isomer (No. 1 to No. 29) in the iso-C_{40} database,[2] obtained with spin-restricted TAO-BLYP. For brevity, the HOMO/LUMO is denoted as the H/L.

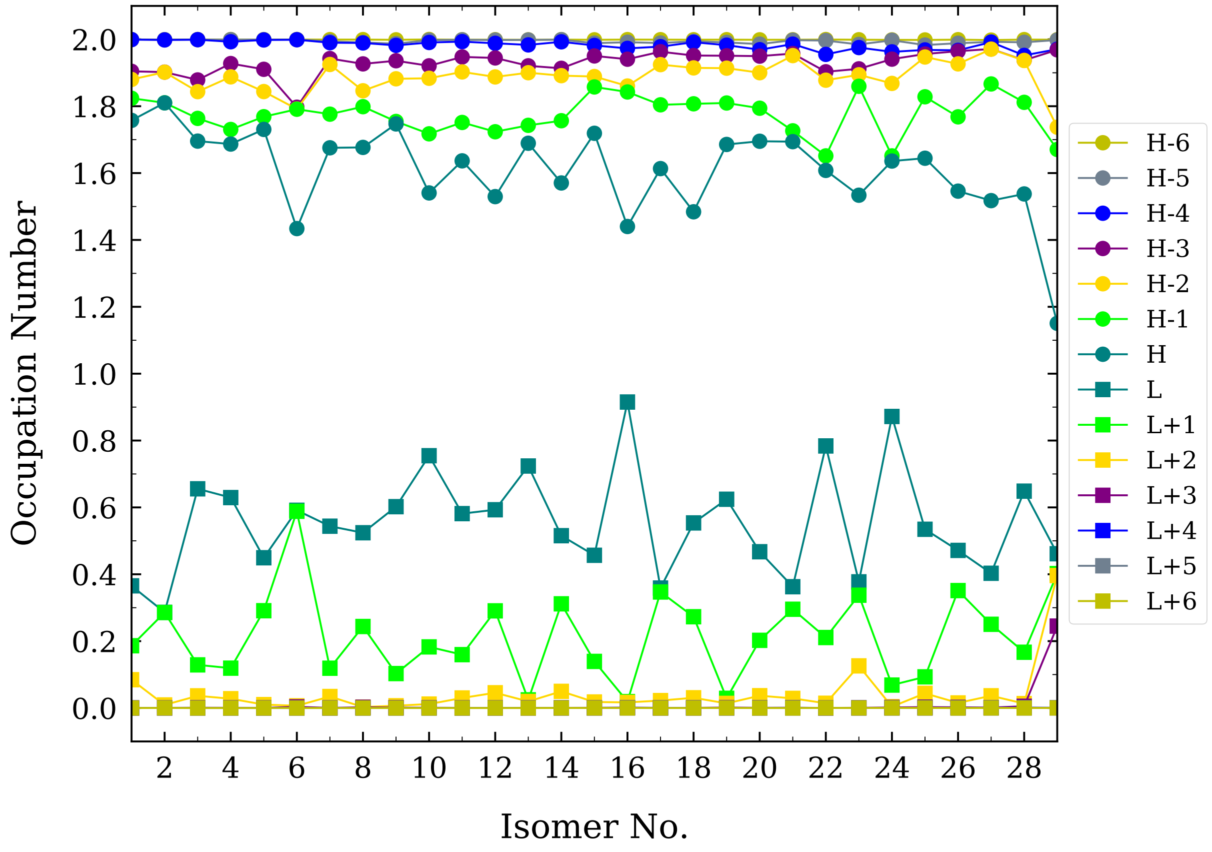

Figure S4: Active TAO orbital occupation numbers (HOMO−6, ..., HOMO−1, HOMO, LUMO, LUMO+1, ..., and LUMO+6) for the lowest singlet state of each C_{40} fullerene isomer (No. 1 to No. 29) in the iso-C_{40} database,[2] obtained with spin-restricted TAO-PBE. For brevity, the HOMO/LUMO is denoted as the H/L.

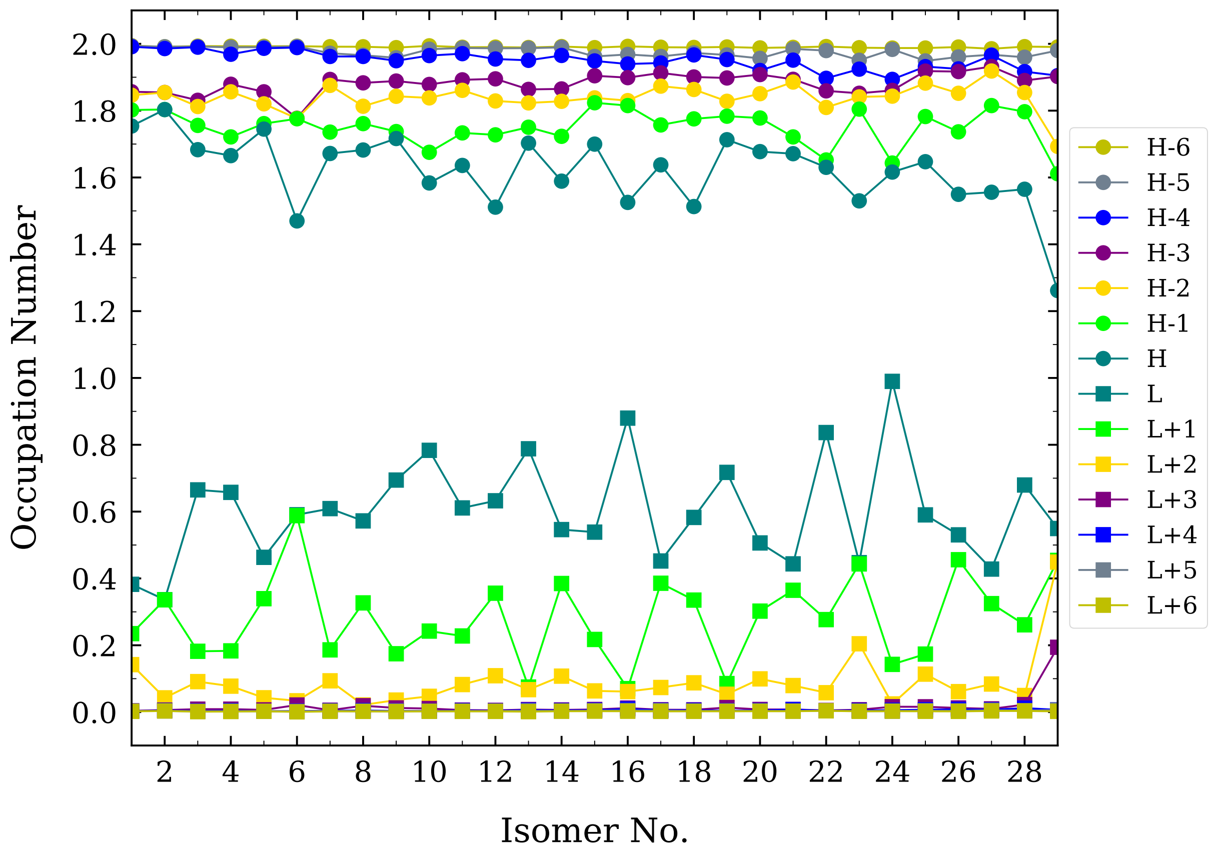

Figure S5: Active TAO orbital occupation numbers (HOMO−6, ..., HOMO−1, HOMO, LUMO, LUMO+1, ..., and LUMO+6) for the lowest singlet state of each C_{40} fullerene isomer (No. 1 to No. 29) in the iso-C_{40} database,[2] obtained with spin-restricted TAO-B3LYP. For brevity, the HOMO/LUMO is denoted as the H/L.

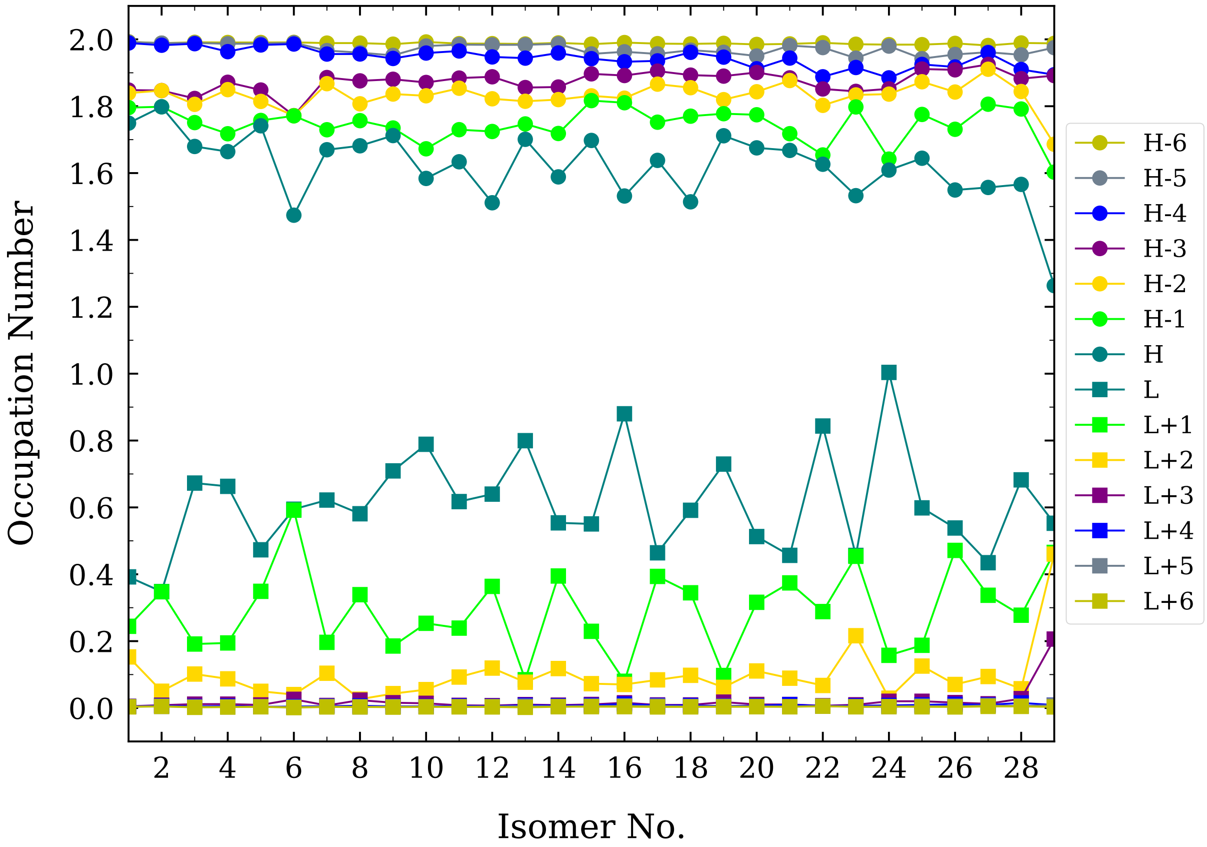

Figure S6: Active TAO orbital occupation numbers (HOMO−6, ..., HOMO−1, HOMO, LUMO, LUMO+1, ..., and LUMO+6) for the lowest singlet state of each C_{40} fullerene isomer (No. 1 to No. 29) in the iso-C_{40} database,[2] obtained with spin-restricted TAO-PBE0. For brevity, the HOMO/LUMO is denoted as the H/L.

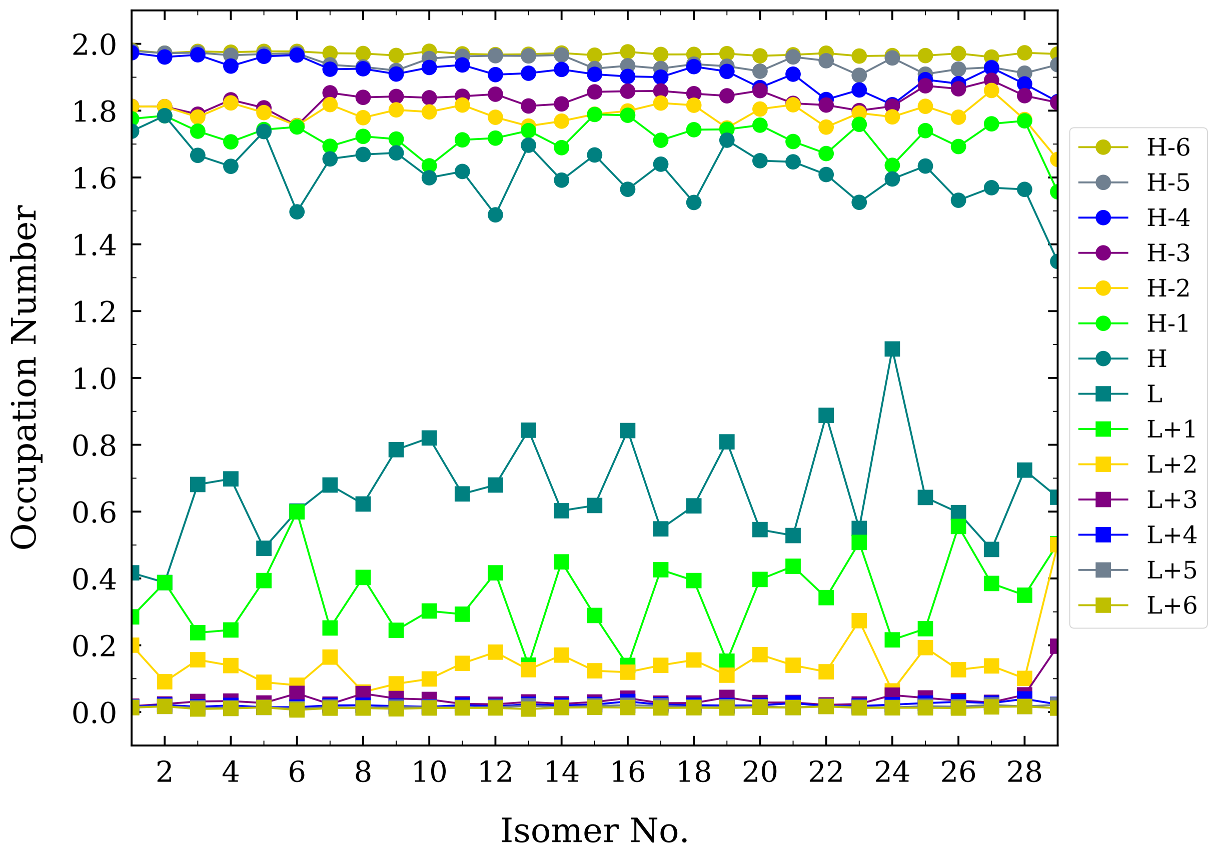

Figure S7: Active TAO orbital occupation numbers (HOMO−6, ..., HOMO−1, HOMO, LUMO, LUMO+1, ..., and LUMO+6) for the lowest singlet state of each C_{40} fullerene isomer (No. 1 to No. 29) in the iso-C_{40} database,[2] obtained with spin-restricted TAO-BHHLYP. For brevity, the HOMO/LUMO is denoted as the H/L.

Table S1: WTMAD-2 values (in kcal/mol) of the GMTKN55 database[3] for KS-ωB97X-D3.[4] The number of data points for each category is given in parenthesis.

	basic + small (473)	iso. + large (243)	barriers (194)	intermol. NCIs (304)	intramol. NCIs (291)	all NCIs (595)	all (1505)
KS-ωB97X-D3[a]	3.32	7.85	4.67	4.54	4.86	4.70	4.77
KS-ωB97X-D3[b]	3.34	7.45	4.56	4.60	4.86	4.72	4.71

[a] Data taken from the SI of the GMTKN55 paper.[3] [b] Data obtained in this work.

Table S2: Statistical errors (in kcal/mol) of the 55 subsets in the GMTKN55 database[3] for TAO-BLYP.

	MSE	MAE	RMS	MIN	MAX
W4-11	-5.77	11.83	14.83	-57.74	31.29
G21EA	-2.97	6.13	7.69	-18.46	9.45
G21IP	-3.58	7.85	9.29	-19.02	15.58
DIPCS10	-7.41	8.73	9.70	-14.59	6.60
PA26	2.05	2.59	3.57	-1.60	9.18
SIE4x4	26.02	26.02	29.54	4.42	53.13
ALKBDE10	3.43	7.13	8.91	-6.51	20.19
YBDE18	-9.22	10.36	13.00	-23.46	8.76
AL2X6	-10.84	10.84	11.34	-15.92	-5.54
HEAVYSB11	-14.92	14.92	15.85	-23.14	-6.59
NBPRC	5.13	6.57	9.08	-3.79	22.81
ALK8	-6.53	6.53	8.59	-16.04	-0.93
RC21	0.14	3.71	5.14	-14.55	9.31
G2RC	4.68	5.63	7.08	-8.07	15.04
BH76RC	-0.63	4.59	5.88	-14.91	8.63
FH51	5.05	6.10	7.86	-18.95	22.89
TAUT15	-0.24	1.65	1.91	-2.67	3.30
DC13	7.34	20.94	24.33	-38.52	36.80
MB16-43	-56.58	56.58	62.13	-109.81	-2.65
DARC	21.16	21.16	21.49	13.21	24.90
RSE43	-3.17	3.17	3.50	-7.67	-0.95
BSR36	-11.17	11.17	12.77	-28.91	-2.81
CDIE20	1.54	1.54	1.66	0.34	2.62
ISO34	-0.91	3.09	4.34	-9.90	12.27
ISOL24	-7.32	13.14	19.54	-63.64	41.18
C60ISO	-17.32	17.32	19.67	-32.15	-4.18
PArel	-0.43	1.74	2.81	-8.36	6.69
BH76	-7.72	7.96	8.92	-18.62	3.47
BHPERI	0.94	3.79	4.43	-11.20	6.51
BHDIV10	-4.13	5.52	6.41	-10.76	4.84
INV24	-4.05	4.15	6.37	-24.95	1.20
BHROT27	0.07	0.40	0.54	-0.57	1.28
PX13	-7.58	7.58	7.67	-10.66	-5.55
WCPT18	-4.59	4.59	4.96	-8.56	-1.74
RG18	-0.82	0.82	1.06	-2.40	-0.06
ADIM6	-5.32	5.32	5.81	-8.90	-2.11
S22	-4.17	4.17	5.34	-14.06	-0.22
S66	-3.51	3.51	4.10	-9.94	-0.28
HEAVY28	-1.21	1.21	1.31	-2.33	-0.32
WATER27	-6.38	6.62	10.16	-28.10	1.76
CARBHB12	-0.08	0.79	0.95	-1.40	1.85
PNICO23	-1.68	1.68	1.82	-3.79	-0.66
HAL59	-1.45	1.70	2.30	-8.42	4.08
AHB21	1.04	1.16	1.57	-1.05	4.38
CHB6	1.13	1.49	1.98	-0.73	4.16
IL16	3.73	3.73	3.83	2.31	5.89
IDISP	6.06	18.20	21.98	-17.64	43.49
ICONF	-0.08	0.64	0.92	-2.12	2.27
ACONF	0.96	0.96	1.08	0.36	2.03
Amino20x4	-0.16	0.71	0.90	-2.10	2.31
PCONF21	-0.75	4.25	4.71	-6.19	7.42
MCONF	-2.66	2.71	3.06	-4.75	0.51
SCONF	-0.27	0.49	0.63	-1.24	1.30
UPU23	1.99	2.73	3.95	-2.42	10.64
BUT14DIOL	-0.09	0.37	0.44	-0.75	1.04

Table S3: Statistical errors (in kcal/mol) of the 55 subsets in the GMTKN55 database[3] for TAO-BLYP-D3(0).

	MSE	MAE	RMS	MIN	MAX
W4-11	-5.34	11.54	14.37	-55.56	31.65
G21EA	-2.97	6.14	7.70	-18.46	9.45
G21IP	-3.59	7.86	9.29	-19.01	15.58
DIPCS10	-7.46	8.78	9.74	-14.45	6.60
PA26	2.54	2.86	3.96	-1.56	9.55
SIE4x4	26.57	26.57	29.97	4.42	53.37
ALKBDE10	3.45	7.12	8.91	-6.50	20.19
YBDE18	-6.21	7.85	10.05	-19.76	10.56
AL2X6	-6.08	6.08	6.29	-8.25	-4.03
HEAVYSB11	-11.96	11.96	13.91	-23.10	-1.21
NBPRC	2.27	4.03	5.44	-3.08	13.74
ALK8	-3.33	3.63	4.35	-8.09	1.23
RC21	2.20	4.44	5.39	-10.23	9.10
G2RC	4.37	5.44	6.70	-8.19	15.20
BH76RC	-0.69	4.52	5.84	-14.91	8.79
FH51	3.49	4.49	5.84	-12.44	15.28
TAUT15	-0.80	1.60	1.95	-3.12	2.67
DC13	5.57	17.48	20.08	-31.56	35.57
MB16-43	-34.13	35.21	41.38	-79.83	11.88
DARC	14.62	14.62	14.71	10.63	16.06
RSE43	-2.64	2.64	2.99	-7.04	-0.56
BSR36	-4.93	4.93	6.23	-17.46	-1.26
CDIE20	1.25	1.25	1.32	-0.01	2.08
ISO34	-0.71	2.63	3.73	-8.52	11.10
ISOL24	-5.37	9.46	12.87	-33.27	29.06
C60ISO	-17.70	17.70	20.17	-32.98	-4.11
PArel	-0.49	1.80	2.89	-9.07	6.55
BH76	-8.43	8.61	9.55	-18.79	2.14
BHPERI	-3.04	3.06	3.92	-11.39	0.27
BHDIV10	-4.50	5.60	6.49	-11.17	4.07
INV24	-2.97	3.13	5.99	-25.45	1.10
BHROT27	0.11	0.39	0.55	-0.92	1.14
PX13	-7.92	7.92	8.06	-11.23	-5.98
WCPT18	-5.56	5.56	6.10	-11.29	-1.38
RG18	0.15	0.15	0.23	0.01	0.57
ADIM6	1.57	1.57	1.72	0.61	2.52
S22	0.78	0.78	0.88	0.20	1.86
S66	0.92	0.92	1.00	0.08	1.99
HEAVY28	0.32	0.35	0.41	-0.36	0.74
WATER27	6.14	6.44	9.37	-4.06	22.86
CARBHB12	1.41	1.41	1.65	0.35	2.98
PNICO23	0.02	0.31	0.38	-0.60	0.70
HAL59	0.59	0.69	1.18	-0.51	5.62
AHB21	-0.00	0.80	1.20	-1.85	4.20
CHB6	0.32	0.88	1.10	-0.84	2.25
IL16	-0.45	0.67	0.83	-1.84	0.61
IDISP	6.08	6.15	10.03	-0.21	23.26
ICONF	-0.06	0.58	0.74	-1.60	1.28
ACONF	-0.05	0.11	0.15	-0.37	0.17
Amino20x4	-0.07	0.31	0.47	-1.06	1.84
PCONF21	-0.21	0.89	1.10	-1.98	1.84
MCONF	0.36	0.51	0.61	-0.76	1.32
SCONF	-0.11	0.62	1.04	-3.39	0.77
UPU23	-0.15	0.51	0.74	-2.30	1.52
BUT14DIOL	0.34	0.37	0.44	-0.41	1.11

Table S4: Statistical errors (in kcal/mol) of the 55 subsets in the GMTKN55 database[3] for TAO-BLYP-D3(BJ).

	MSE	MAE	RMS	MIN	MAX
W4-11	-3.68	10.84	13.35	-50.93	33.49
G21EA	-2.97	6.12	7.69	-18.46	9.45
G21IP	-3.59	7.86	9.30	-19.02	15.58
DIPCS10	-7.38	8.70	9.66	-14.51	6.60
PA26	2.84	3.05	4.12	-1.07	9.87
SIE4x4	26.55	26.55	29.91	4.68	53.19
ALKBDE10	4.24	7.24	9.30	-5.91	21.51
YBDE18	-4.34	6.86	8.56	-17.28	11.01
AL2X6	-3.30	3.30	3.64	-6.57	-2.19
HEAVYSB11	-9.49	10.00	12.08	-21.10	2.83
NBPRC	1.41	2.78	4.01	-2.66	10.55
ALK8	1.23	1.87	2.53	-1.29	5.34
RC21	2.92	4.49	5.29	-8.22	8.72
G2RC	4.04	5.11	6.50	-8.44	15.51
BH76RC	-0.63	4.48	5.80	-14.67	9.24
FH51	3.38	4.41	5.85	-9.82	16.65
TAUT15	-0.39	1.71	1.99	-2.89	3.69
DC13	4.45	15.20	17.50	-24.36	32.94
MB16-43	-15.19	22.81	27.75	-67.91	35.26
DARC	11.76	11.76	11.91	7.26	13.41
RSE43	-2.87	2.87	3.21	-7.01	-0.75
BSR36	-2.36	2.36	2.83	-7.18	-0.84
CDIE20	1.21	1.21	1.28	0.05	1.79
ISO34	-0.52	2.49	3.56	-8.24	10.94
ISOL24	-4.11	8.75	11.63	-26.31	28.48
C60ISO	-16.63	16.63	19.13	-31.85	-3.54
PArel	-0.45	1.77	2.73	-8.08	6.30
BH76	-8.70	8.82	9.74	-19.50	1.59
BHPERI	-4.65	4.65	5.12	-10.82	-1.62
BHDIV10	-5.60	6.54	7.46	-12.55	4.66
INV24	-2.52	3.09	5.75	-24.34	2.92
BHROT27	0.14	0.41	0.57	-0.59	1.32
PX13	-8.56	8.56	8.71	-12.25	-6.64
WCPT18	-6.05	6.05	6.60	-12.01	-1.80
RG18	0.05	0.15	0.22	-0.54	0.46
ADIM6	0.76	0.76	0.86	0.20	1.24
S22	0.69	0.69	0.83	0.06	1.74
S66	0.64	0.64	0.70	0.05	1.36
HEAVY28	0.72	0.72	0.77	-0.04	1.18
WATER27	4.86	5.31	7.63	-6.13	18.82
CARBHB12	1.53	1.53	1.77	0.62	3.26
PNICO23	1.03	1.03	1.27	0.20	3.31
HAL59	1.22	1.23	1.64	-0.12	5.91
AHB21	-0.19	0.91	1.27	-2.01	4.04
CHB6	-0.43	1.04	1.23	-1.73	1.83
IL16	-0.29	0.44	0.52	-0.91	0.77
IDISP	4.84	4.84	7.28	0.79	16.81
ICONF	-0.16	0.33	0.41	-0.94	0.44
ACONF	-0.22	0.22	0.28	-0.63	-0.04
Amino20x4	-0.05	0.34	0.45	-1.05	1.49
PCONF21	-0.33	0.89	0.97	-1.55	1.34
MCONF	0.22	0.49	0.59	-1.12	1.25
SCONF	0.07	0.73	1.09	-3.43	0.91
UPU23	0.12	0.47	0.67	-1.78	1.43
BUT14DIOL	0.50	0.51	0.56	-0.22	1.21

Table S5: Statistical errors (in kcal/mol) of the 55 subsets in the GMTKN55 database[3] for TAO-BLYP-D4.

	MSE	MAE	RMS	MIN	MAX
W4-11	-3.68	10.82	13.31	-50.98	33.41
G21EA	-2.47	5.80	7.43	-18.46	9.45
G21IP	-3.39	7.67	9.13	-18.80	15.58
DIPCS10	-6.50	7.82	8.66	-12.89	6.60
PA26	1.55	2.28	3.10	-1.64	8.08
SIE4x4	26.40	26.40	29.82	4.45	53.13
ALKBDE10	4.32	7.37	9.50	-5.94	22.14
YBDE18	-4.10	6.76	8.39	-17.05	11.25
AL2X6	-3.79	3.79	4.06	-6.84	-2.59
HEAVYSB11	-9.70	10.11	12.19	-21.14	2.27
NBPRC	1.23	2.66	3.63	-2.79	9.23
ALK8	-4.49	4.84	6.83	-15.33	1.40
RC21	2.89	4.41	5.20	-8.12	8.60
G2RC	4.04	5.21	6.68	-8.76	15.79
BH76RC	-0.63	4.49	5.83	-14.71	9.45
FH51	3.26	4.30	5.73	-8.65	16.79
TAUT15	-0.20	1.66	1.96	-2.66	3.95
DC13	5.05	13.22	15.33	-22.93	30.17
MB16-43	-17.12	25.04	30.49	-72.48	39.63
DARC	10.72	10.72	10.89	6.29	12.49
RSE43	-2.93	2.93	3.26	-7.13	-0.92
BSR36	-0.74	0.83	0.93	-1.92	1.51
CDIE20	1.29	1.29	1.35	0.18	1.81
ISO34	-0.56	2.43	3.47	-8.63	10.42
ISOL24	-3.87	7.94	10.58	-23.02	25.70
C60ISO	-18.63	18.63	21.24	-34.75	-4.30
PArel	-0.34	1.71	2.67	-7.59	6.65
BH76	-8.74	8.88	9.79	-19.50	1.76
BHPERI	-5.17	5.17	5.58	-10.58	-1.78
BHDIV10	-5.77	6.66	7.58	-13.00	4.42
INV24	-2.79	3.01	5.73	-24.30	1.28
BHROT27	0.15	0.43	0.58	-0.59	1.32
PX13	-8.59	8.59	8.74	-12.22	-6.60
WCPT18	-6.10	6.10	6.64	-12.10	-1.81
RG18	-0.01	0.13	0.19	-0.57	0.38
ADIM6	0.30	0.30	0.34	0.05	0.55
S22	0.78	0.81	1.10	-0.28	2.55
S66	0.56	0.56	0.70	-0.07	1.91
HEAVY28	0.50	0.53	0.59	-0.22	1.27
WATER27	4.73	5.20	7.27	-6.37	18.48
CARBHB12	1.38	1.38	1.64	0.48	3.10
PNICO23	0.95	0.95	1.17	0.19	3.04
HAL59	1.28	1.28	1.67	-0.02	5.90
AHB21	-0.43	0.98	1.26	-1.97	3.77
CHB6	0.57	1.43	1.81	-1.21	2.98
IL16	-1.09	1.09	1.25	-2.46	-0.09
IDISP	4.00	4.00	6.13	0.58	13.67
ICONF	-0.22	0.32	0.41	-0.92	0.25
ACONF	-0.31	0.31	0.36	-0.76	-0.09
Amino20x4	-0.05	0.38	0.51	-1.29	1.72
PCONF21	-0.24	0.86	0.98	-1.64	1.52
MCONF	0.25	0.56	0.65	-1.19	1.23
SCONF	0.21	0.98	1.32	-3.92	1.14
UPU23	0.10	0.45	0.61	-1.47	1.43
BUT14DIOL	0.81	0.81	0.84	-0.00	1.28

Table S6: Statistical errors (in kcal/mol) of the 55 subsets in the GMTKN55 database[3] for TAO-PBE.

	MSE	MAE	RMS	MIN	MAX
W4-11	3.84	10.86	14.59	-38.34	42.99
G21EA	-1.58	4.29	5.29	-11.75	8.97
G21IP	-1.85	6.66	7.86	-17.11	15.74
DIPCS10	-4.03	5.11	6.40	-13.83	3.82
PA26	1.78	2.16	2.91	-1.68	7.66
SIE4x4	24.75	24.75	27.81	4.45	47.53
ALKBDE10	5.44	7.67	10.79	-4.89	27.00
YBDE18	-1.40	5.69	6.68	-11.53	10.74
AL2X6	-3.09	3.45	3.96	-6.62	1.09
HEAVYSB11	-7.74	7.74	8.66	-14.43	-1.88
NBPRC	0.42	2.78	3.34	-6.34	5.49
ALK8	2.71	3.81	5.68	-2.32	13.37
RC21	6.02	6.29	7.39	-1.91	13.14
G2RC	0.61	6.56	7.93	-13.61	19.39
BH76RC	-0.30	4.27	5.98	-13.13	19.50
FH51	1.44	3.33	4.62	-10.95	12.88
TAUT15	0.30	1.86	2.39	-2.64	5.21
DC13	1.42	10.49	14.34	-37.07	18.50
MB16-43	1.94	21.75	27.66	-77.21	57.29
DARC	4.52	5.07	5.82	-2.75	8.06
RSE43	-3.14	3.14	3.45	-7.75	-0.83
BSR36	-7.12	7.12	8.29	-18.71	-2.01
CDIE20	1.72	1.72	1.87	0.14	3.01
ISO34	-0.94	1.72	2.33	-6.02	4.30
ISOL24	-3.40	6.42	9.55	-28.60	21.67
C60ISO	-18.19	18.19	20.71	-34.11	-4.31
PArel	0.30	1.91	2.58	-5.89	6.95
BH76	-8.64	8.70	9.83	-27.19	2.26
BHPERI	-5.04	5.04	5.32	-8.65	-2.13
BHDIV10	-8.12	8.54	9.54	-14.79	2.12
INV24	-3.69	3.69	6.31	-25.99	-0.02
BHROT27	0.34	0.44	0.63	-0.44	1.65
PX13	-12.06	12.06	12.26	-17.68	-9.07
WCPT18	-9.12	9.12	9.56	-16.31	-5.03
RG18	0.01	0.25	0.32	-0.71	0.65
ADIM6	-2.66	2.66	2.98	-4.76	-0.82
S22	-1.97	2.07	3.06	-8.91	0.65
S66	-1.55	1.67	2.17	-6.18	0.82
HEAVY28	-0.10	0.31	0.38	-0.84	0.53
WATER27	4.30	5.10	6.33	-10.76	18.30
CARBHB12	1.59	1.59	1.98	0.32	4.10
PNICO23	0.44	0.78	1.44	-1.77	5.70
HAL59	0.22	1.20	1.81	-5.18	6.31
AHB21	-1.09	1.32	1.56	-3.16	1.34
CHB6	-0.17	0.74	0.97	-1.36	1.69
IL16	0.40	0.82	0.99	-1.20	2.18
IDISP	3.14	9.83	11.58	-9.69	22.10
ICONF	0.15	0.39	0.58	-0.51	1.92
ACONF	0.49	0.49	0.56	0.18	1.12
Amino20x4	0.02	0.47	0.60	-1.26	1.67
PCONF21	-0.81	3.18	3.43	-4.81	4.91
MCONF	-1.43	1.56	1.78	-3.15	0.94
SCONF	0.20	0.41	0.57	-1.51	0.99
UPU23	1.20	1.83	2.59	-1.98	6.78
BUT14DIOL	0.24	0.28	0.37	-0.27	1.16

Table S7: Statistical errors (in kcal/mol) of the 55 subsets in the GMTKN55 database[3] for TAO-PBE-D3(0).

	MSE	MAE	RMS	MIN	MAX
W4-11	4.02	10.83	14.56	-37.48	43.14
G21EA	-1.58	4.30	5.30	-11.75	8.97
G21IP	-1.86	6.66	7.86	-17.11	15.74
DIPCS10	-4.05	5.13	6.41	-13.80	3.82
PA26	2.00	2.28	3.06	-1.50	7.85
SIE4x4	25.02	25.02	28.05	4.45	47.62
ALKBDE10	5.45	7.66	10.79	-4.89	27.00
YBDE18	-0.03	5.06	5.93	-9.90	11.56
AL2X6	-0.79	1.88	2.63	-5.67	1.72
HEAVYSB11	-6.32	6.44	7.87	-14.42	0.65
NBPRC	-0.90	2.62	3.19	-6.99	4.38
ALK8	4.21	4.64	6.97	-1.05	14.92
RC21	6.98	7.30	8.42	-2.04	14.11
G2RC	0.48	6.85	8.22	-15.21	19.46
BH76RC	-0.32	4.31	6.01	-13.13	19.57
FH51	0.72	3.14	4.49	-11.71	12.89
TAUT15	0.06	1.84	2.25	-2.75	4.94
DC13	0.53	9.39	13.45	-37.10	17.89
MB16-43	12.41	23.38	29.97	-65.97	71.69
DARC	1.39	2.76	3.04	-4.02	4.25
RSE43	-2.91	2.91	3.23	-7.49	-0.73
BSR36	-3.72	3.72	4.69	-13.07	-1.03
CDIE20	1.60	1.60	1.72	0.35	2.78
ISO34	-0.84	1.50	2.02	-5.55	3.86
ISOL24	-2.52	4.66	6.51	-13.23	15.56
C60ISO	-18.32	18.32	20.90	-34.43	-4.25
PArel	0.27	1.88	2.57	-6.24	6.88
BH76	-8.96	9.01	10.13	-27.44	2.11
BHPERI	-6.88	6.88	7.11	-10.03	-3.06
BHDIV10	-8.26	8.64	9.66	-14.84	1.88
INV24	-3.19	3.21	6.16	-26.17	0.21
BHROT27	0.34	0.40	0.60	-0.28	1.60
PX13	-12.20	12.20	12.44	-18.09	-9.17
WCPT18	-9.55	9.55	10.10	-17.56	-4.88
RG18	0.53	0.53	0.64	0.18	1.78
ADIM6	1.27	1.27	1.33	0.66	1.75
S22	0.68	0.73	0.87	-0.58	1.87
S66	0.87	0.87	0.96	-0.01	2.00
HEAVY28	0.58	0.58	0.64	0.15	1.20
WATER27	11.12	11.79	16.25	-9.05	38.93
CARBHB12	2.35	2.35	2.67	0.89	4.64
PNICO23	1.27	1.27	1.82	0.16	6.34
HAL59	1.21	1.21	1.86	-0.13	6.95
AHB21	-1.58	1.70	1.87	-3.36	1.27
CHB6	-0.48	0.80	0.88	-1.45	0.96
IL16	-1.65	1.65	1.69	-2.40	-1.04
IDISP	3.16	3.43	4.96	-0.82	10.85
ICONF	0.15	0.37	0.54	-0.45	1.68
ACONF	-0.02	0.08	0.10	-0.21	0.23
Amino20x4	0.05	0.32	0.41	-0.90	1.25
PCONF21	-0.55	0.95	1.18	-2.12	1.11
MCONF	0.24	0.49	0.58	-0.62	1.19
SCONF	0.24	0.81	1.04	-3.02	1.04
UPU23	-0.07	0.43	0.58	-1.41	1.21
BUT14DIOL	0.48	0.49	0.55	-0.17	1.22

Table S8: Statistical errors (in kcal/mol) of the 55 subsets in the GMTKN55 database[3] for TAO-PBE-D3(BJ).

	MSE	MAE	RMS	MIN	MAX
W4-11	4.80	10.95	14.74	-35.50	44.04
G21EA	-1.58	4.29	5.29	-11.75	8.97
G21IP	-1.86	6.66	7.87	-17.12	15.74
DIPCS10	-4.01	5.10	6.38	-13.79	3.82
PA26	2.19	2.41	3.17	-1.31	8.03
SIE4x4	25.03	25.03	28.03	4.59	47.56
ALKBDE10	5.78	7.82	11.02	-4.82	27.53
YBDE18	0.91	4.92	5.74	-8.71	11.83
AL2X6	0.42	2.10	2.55	-4.92	2.53
HEAVYSB11	-5.26	5.80	7.20	-13.63	2.45
NBPRC	-1.39	3.01	3.57	-7.83	3.33
ALK8	5.96	6.25	8.72	-1.07	18.33
RC21	7.41	7.72	8.87	-2.00	14.48
G2RC	0.28	7.23	8.63	-18.03	19.61
BH76RC	-0.31	4.36	6.06	-13.02	19.79
FH51	0.59	3.23	4.63	-12.23	12.96
TAUT15	0.21	1.89	2.38	-2.68	5.38
DC13	0.01	9.73	13.96	-39.30	17.99
MB16-43	20.22	27.57	35.40	-61.05	91.13
DARC	-0.13	2.33	2.73	-5.62	2.87
RSE43	-2.98	2.98	3.29	-7.44	-0.68
BSR36	-2.61	2.61	3.28	-9.13	-0.77
CDIE20	1.56	1.56	1.67	0.32	2.61
ISO34	-0.75	1.43	1.95	-5.32	3.69
ISOL24	-1.84	4.02	5.90	-11.98	15.00
C60ISO	-17.85	17.85	20.45	-33.95	-3.99
PArel	0.28	1.91	2.55	-5.81	6.76
BH76	-9.12	9.18	10.28	-27.88	2.08
BHPERI	-7.77	7.77	8.06	-11.27	-3.24
BHDIV10	-8.77	9.18	10.25	-16.62	2.03
INV24	-2.93	3.17	6.06	-25.76	1.77
BHROT27	0.36	0.43	0.64	-0.42	1.66
PX13	-12.54	12.54	12.78	-18.51	-9.58
WCPT18	-9.84	9.84	10.42	-18.04	-5.06
RG18	0.46	0.46	0.54	0.17	1.35
ADIM6	0.93	0.93	0.97	0.52	1.28
S22	0.72	0.76	0.90	-0.42	1.94
S66	0.80	0.80	0.89	0.00	2.13
HEAVY28	0.75	0.75	0.81	0.31	1.49
WATER27	10.52	11.25	15.33	-9.86	37.80
CARBHB12	2.43	2.43	2.75	1.01	4.82
PNICO23	1.75	1.75	2.31	0.45	7.48
HAL59	1.48	1.48	2.10	-0.04	7.17
AHB21	-1.71	1.82	2.03	-3.88	1.17
CHB6	-0.75	1.06	1.13	-1.79	0.92
IL16	-1.67	1.67	1.75	-2.67	-0.89
IDISP	2.65	2.65	3.56	0.57	7.78
ICONF	0.12	0.30	0.39	-0.51	0.91
ACONF	-0.12	0.13	0.16	-0.37	0.06
Amino20x4	0.07	0.37	0.46	-0.91	1.06
PCONF21	-0.61	1.04	1.37	-2.52	1.13
MCONF	0.17	0.53	0.61	-0.84	1.22
SCONF	0.34	0.95	1.17	-3.16	1.22
UPU23	0.08	0.44	0.57	-1.15	1.48
BUT14DIOL	0.54	0.55	0.60	-0.16	1.30

Table S9: Statistical errors (in kcal/mol) of the 55 subsets in the GMTKN55 database[3] for TAO-PBE-D4.

	MSE	MAE	RMS	MIN	MAX
W4-11	4.75	10.95	14.73	-35.11	43.89
G21EA	-1.37	4.19	5.21	-11.75	8.97
G21IP	-1.77	6.59	7.80	-17.02	15.74
DIPCS10	-3.64	4.76	6.01	-13.03	3.82
PA26	1.56	2.03	2.72	-1.80	7.17
SIE4x4	24.93	24.93	27.96	4.47	47.53
ALKBDE10	5.84	7.87	11.13	-4.84	27.88
YBDE18	0.98	4.85	5.68	-8.52	11.84
AL2X6	0.51	2.13	2.55	-4.85	2.51
HEAVYSB11	-5.04	5.72	7.09	-13.51	2.83
NBPRC	-1.43	3.04	3.53	-7.80	2.87
ALK8	3.77	4.16	5.99	-1.26	13.92
RC21	7.30	7.60	8.76	-2.00	14.40
G2RC	0.34	7.25	8.67	-18.34	19.72
BH76RC	-0.30	4.35	6.06	-13.04	19.86
FH51	0.59	3.22	4.63	-12.30	12.97
TAUT15	0.30	1.85	2.38	-2.63	5.46
DC13	0.30	10.03	14.26	-38.63	18.35
MB16-43	21.20	28.55	36.52	-58.18	95.86
DARC	-0.51	2.25	2.75	-5.91	2.50
RSE43	-3.02	3.02	3.33	-7.51	-0.77
BSR36	-1.75	1.75	2.17	-5.83	-0.45
CDIE20	1.62	1.62	1.73	0.29	2.65
ISO34	-0.78	1.42	1.96	-5.56	3.57
ISOL24	-1.80	3.87	5.62	-12.52	13.46
C60ISO	-18.92	18.92	21.59	-35.54	-4.38
PArel	0.33	1.87	2.53	-5.58	6.93
BH76	-9.10	9.16	10.26	-27.81	2.15
BHPERI	-7.90	7.90	8.21	-11.50	-3.23
BHDIV10	-8.81	9.20	10.27	-16.70	1.95
INV24	-3.08	3.14	6.05	-25.75	0.46
BHROT27	0.36	0.43	0.64	-0.41	1.66
PX13	-12.46	12.46	12.70	-18.38	-9.46
WCPT18	-9.79	9.79	10.35	-17.91	-5.05
RG18	0.46	0.46	0.54	0.16	1.30
ADIM6	0.73	0.73	0.75	0.44	0.99
S22	0.86	0.86	1.00	0.30	2.14
S66	0.81	0.81	0.90	0.25	2.25
HEAVY28	0.79	0.79	0.85	0.25	1.59
WATER27	9.99	10.73	14.48	-9.99	36.19
CARBHB12	2.37	2.37	2.70	0.99	4.75
PNICO23	1.79	1.79	2.33	0.49	7.47
HAL59	1.63	1.63	2.20	0.07	7.14
AHB21	-1.80	1.90	2.11	-4.05	1.08
CHB6	-0.46	0.81	0.83	-0.99	1.04
IL16	-2.05	2.05	2.18	-3.19	-1.13
IDISP	2.26	2.26	2.83	0.43	5.75
ICONF	0.08	0.28	0.34	-0.49	0.75
ACONF	-0.15	0.15	0.19	-0.43	0.00
Amino20x4	0.07	0.37	0.46	-0.90	1.25
PCONF21	-0.53	0.89	1.14	-2.15	0.93
MCONF	0.22	0.52	0.63	-0.92	1.27
SCONF	0.38	1.03	1.25	-3.37	1.26
UPU23	0.06	0.43	0.56	-1.12	1.48
BUT14DIOL	0.67	0.68	0.71	-0.10	1.26

Table S10: Statistical errors (in kcal/mol) of the 55 subsets in the GMTKN55 database[3] for TAO-B97-D4.

	MSE	MAE	RMS	MIN	MAX
W4-11	-0.66	8.84	11.69	-43.97	33.87
G21EA	6.78	7.42	8.76	-6.01	16.61
G21IP	8.25	9.01	10.92	-6.36	23.25
DIPCS10	17.04	17.04	17.43	12.89	23.77
PA26	6.15	6.15	6.63	2.62	12.76
SIE4x4	25.83	25.83	29.02	6.58	51.24
ALKBDE10	4.68	7.08	10.46	-5.45	28.54
YBDE18	-0.77	5.37	6.14	-11.83	9.61
AL2X6	-4.40	4.40	5.20	-10.39	-2.43
HEAVYSB11	-7.37	7.51	9.47	-17.76	0.82
NBPRC	1.24	2.46	3.14	-3.74	6.38
ALK8	-0.61	2.55	4.15	-9.17	7.06
RC21	5.28	5.53	6.57	-1.92	11.23
G2RC	2.09	6.06	7.52	-13.73	19.90
BH76RC	-0.46	4.09	5.43	-11.37	15.52
FH51	1.50	3.13	4.31	-7.74	12.82
TAUT15	-0.67	1.61	1.91	-3.30	2.80
DC13	3.64	11.45	13.56	-24.54	23.09
MB16-43	-2.00	25.83	32.56	-81.08	67.78
DARC	2.20	3.20	3.50	-2.96	5.35
RSE43	-3.25	3.25	3.57	-7.80	-1.28
BSR36	-2.38	2.38	2.69	-5.88	-0.15
CDIE20	1.72	1.72	1.84	0.23	2.89
ISO34	-0.89	1.62	2.14	-4.94	4.07
ISOL24	-2.68	4.82	6.55	-16.63	11.47
C60ISO	-18.73	18.73	21.44	-35.50	-4.19
PArel	0.24	1.44	2.28	-6.52	6.15
BH76	-5.90	6.16	7.03	-19.81	3.66
BHPERI	-4.22	4.22	4.65	-7.65	-0.73
BHDIV10	-5.62	6.06	6.89	-10.71	2.23
INV24	-2.52	2.84	5.82	-25.29	2.04
BHROT27	0.33	0.40	0.60	-0.27	1.52
PX13	-4.59	4.59	4.67	-6.25	-3.58
WCPT18	-4.86	4.86	5.45	-10.16	-1.35
RG18	0.08	0.11	0.13	-0.15	0.25
ADIM6	-0.86	0.86	1.01	-1.75	-0.19
S22	-0.64	0.66	0.86	-1.83	0.10
S66	-0.64	0.65	0.78	-1.77	0.09
HEAVY28	0.06	0.24	0.31	-0.77	0.60
WATER27	-8.68	8.73	13.03	-33.87	0.60
CARBHB12	0.82	0.91	1.26	-0.29	2.65
PNICO23	0.21	0.53	0.95	-0.98	3.90
HAL59	0.09	0.52	0.79	-1.17	3.76
AHB21	0.36	1.09	1.43	-2.47	3.79
CHB6	1.93	1.93	2.50	0.44	4.54
IL16	0.06	1.12	1.20	-1.70	1.50
IDISP	2.87	4.70	5.24	-4.92	7.85
ICONF	-0.04	0.39	0.54	-1.28	1.05
ACONF	0.08	0.09	0.12	-0.02	0.36
Amino20x4	-0.18	0.34	0.46	-1.22	1.23
PCONF21	-0.42	0.88	0.97	-1.68	1.61
MCONF	-0.45	0.50	0.60	-1.27	0.38
SCONF	-0.34	0.42	0.54	-1.12	0.23
UPU23	0.35	0.80	0.97	-1.65	1.86
BUT14DIOL	-0.08	0.22	0.28	-0.59	0.68

Table S11: Statistical errors (in kcal/mol) of the 55 subsets in the GMTKN55 database[3] for TAO-B3LYP.

	MSE	MAE	RMS	MIN	MAX
W4-11	-0.25	4.89	6.64	-28.54	14.62
G21EA	-2.14	4.03	5.07	-13.96	7.55
G21IP	-0.41	4.69	5.70	-10.42	12.10
DIPCS10	-1.57	4.00	4.72	-8.44	5.47
PA26	3.21	3.24	4.05	-0.44	10.43
SIE4x4	19.71	19.71	22.48	3.77	41.13
ALKBDE10	2.86	3.79	5.59	-2.09	12.87
YBDE18	-5.78	6.27	7.80	-14.92	3.26
AL2X6	-6.26	6.26	6.77	-10.36	-2.60
HEAVYSB11	-9.16	9.16	9.71	-15.60	-3.98
NBPRC	2.82	3.81	5.30	-3.17	12.57
ALK8	-1.13	1.92	2.95	-5.43	3.17
RC21	2.19	3.06	3.59	-6.18	5.66
G2RC	-0.03	2.89	3.49	-5.26	7.32
BH76RC	-0.92	2.66	3.38	-8.13	3.89
FH51	1.85	3.17	4.15	-11.74	9.48
TAUT15	-0.09	1.28	1.59	-2.36	2.85
DC13	2.78	13.73	16.47	-28.99	22.99
MB16-43	-34.62	34.77	40.26	-88.02	1.91
DARC	11.46	11.46	11.93	3.73	14.51
RSE43	-2.14	2.14	2.39	-5.16	-0.77
BSR36	-10.10	10.10	11.66	-26.35	-3.52
CDIE20	1.33	1.33	1.49	0.08	2.71
ISO34	-0.43	1.91	2.83	-7.90	10.14
ISOL24	-4.43	8.76	12.96	-40.87	29.44
C60ISO	-11.58	11.58	13.67	-23.49	-2.22
PArel	0.10	1.18	1.93	-3.38	6.35
BH76	-5.16	5.26	5.97	-11.76	2.60
BHPERI	1.52	2.40	2.80	-3.99	4.95
BHDIV10	-1.85	3.28	3.73	-5.61	5.08
INV24	-2.74	2.75	4.73	-19.86	0.09
BHROT27	0.33	0.47	0.66	-0.45	1.53
PX13	-4.75	4.75	4.91	-7.20	-2.62
WCPT18	-2.20	2.30	2.83	-5.64	0.83
RG18	-0.30	0.33	0.47	-1.08	0.10
ADIM6	-3.37	3.37	3.72	-5.84	-1.18
S22	-2.42	2.49	3.58	-10.11	0.37
S66	-1.97	2.03	2.60	-6.84	0.43
HEAVY28	-0.52	0.52	0.62	-1.31	-0.01
WATER27	1.38	1.87	2.43	-4.61	6.82
CARBHB12	0.73	0.80	1.15	-0.41	2.94
PNICO23	-0.72	0.72	0.87	-2.55	-0.14
HAL59	-0.79	1.00	1.48	-5.89	2.85
AHB21	-0.83	0.99	1.09	-2.05	1.07
CHB6	-0.35	1.14	1.36	-1.83	2.18
IL16	1.58	1.64	1.84	-0.45	3.79
IDISP	4.26	14.03	16.65	-14.67	31.85
ICONF	-0.01	0.43	0.64	-1.67	1.39
ACONF	0.67	0.67	0.75	0.24	1.39
Amino20x4	-0.04	0.53	0.67	-1.33	1.93
PCONF21	-0.44	3.07	3.43	-4.32	5.33
MCONF	-1.81	1.88	2.15	-3.48	0.48
SCONF	-0.27	0.41	0.51	-0.94	1.06
UPU23	1.59	2.08	2.99	-1.51	7.51
BUT14DIOL	0.04	0.21	0.29	-0.39	0.74

Table S12: Statistical errors (in kcal/mol) of the 55 subsets in the GMTKN55 database[3] for TAO-B3LYP-D3(0).

	MSE	MAE	RMS	MIN	MAX
W4-11	0.11	5.00	6.61	-26.73	14.90
G21EA	-2.14	4.04	5.09	-13.96	7.71
G21IP	-0.41	4.69	5.70	-10.42	12.10
DIPCS10	-1.61	3.98	4.71	-8.42	5.47
PA26	3.59	3.62	4.40	-0.44	10.72
SIE4x4	20.14	20.14	22.81	3.77	41.32
ALKBDE10	2.88	3.80	5.59	-2.09	12.87
YBDE18	-3.35	4.17	5.32	-10.30	4.75
AL2X6	-2.43	2.43	2.57	-3.40	-1.26
HEAVYSB11	-6.71	6.71	7.73	-13.16	-1.63
NBPRC	0.49	2.50	3.11	-4.54	5.42
ALK8	1.71	1.78	2.43	-0.24	4.22
RC21	3.82	4.26	4.77	-2.88	8.19
G2RC	-0.27	3.14	3.80	-5.85	7.46
BH76RC	-0.95	2.66	3.38	-8.16	3.88
FH51	0.60	2.26	3.15	-6.58	9.46
TAUT15	-0.50	1.30	1.53	-2.58	2.46
DC13	1.45	11.02	13.26	-22.79	20.22
MB16-43	-15.69	21.01	25.00	-67.09	20.12
DARC	6.28	6.28	6.50	1.77	7.66
RSE43	-1.73	1.73	2.01	-4.67	-0.45
BSR36	-4.77	4.77	6.15	-17.78	-1.09
CDIE20	1.07	1.08	1.19	-0.05	2.23
ISO34	-0.29	1.55	2.29	-3.43	9.38
ISOL24	-2.83	5.82	7.85	-16.07	20.27
C60ISO	-11.97	11.97	14.12	-24.25	-2.20
PArel	0.07	1.22	1.94	-3.57	6.26
BH76	-5.74	5.82	6.48	-11.91	2.31
BHPERI	-1.67	1.77	2.06	-4.17	1.08
BHDIV10	-2.21	3.39	3.81	-5.93	4.55
INV24	-1.88	2.05	4.52	-20.23	1.77
BHROT27	0.37	0.43	0.65	-0.52	1.64
PX13	-4.93	4.93	5.11	-7.47	-2.92
WCPT18	-2.98	3.13	3.96	-7.82	1.13
RG18	0.48	0.48	0.53	0.13	1.08
ADIM6	2.08	2.08	2.25	0.88	3.32
S22	1.54	1.54	1.67	0.45	2.68
S66	1.56	1.56	1.63	0.72	2.86
HEAVY28	0.80	0.80	0.83	0.28	1.24
WATER27	11.70	11.82	16.86	-1.69	40.16
CARBHB12	1.94	1.94	2.15	0.79	3.84
PNICO23	0.66	0.66	0.73	0.12	1.33
HAL59	0.94	0.94	1.15	0.08	4.11
AHB21	-1.67	1.67	1.71	-2.72	-1.18
CHB6	-1.08	1.24	1.33	-1.92	0.47
IL16	-1.81	1.81	1.86	-2.49	-1.04
IDISP	4.10	5.12	6.90	-3.06	15.07
ICONF	0.01	0.45	0.57	-1.16	1.05
ACONF	-0.15	0.15	0.19	-0.42	-0.02
Amino20x4	0.05	0.25	0.35	-0.77	1.44
PCONF21	-0.01	0.82	1.11	-1.74	2.09
MCONF	0.61	0.66	0.77	-0.50	1.35
SCONF	-0.08	0.47	0.78	-2.55	0.53
UPU23	-0.23	0.70	0.95	-3.16	1.25
BUT14DIOL	0.41	0.41	0.45	-0.10	0.81

Table S13: Statistical errors (in kcal/mol) of the 55 subsets in the GMTKN55 database[3] for TAO-B3LYP-D3(BJ).

	MSE	MAE	RMS	MIN	MAX
W4-11	1.46	5.25	6.62	-22.79	16.39
G21EA	-2.14	4.03	5.07	-13.96	7.46
G21IP	-0.41	4.70	5.70	-10.42	12.10
DIPCS10	-1.54	3.98	4.69	-8.36	5.47
PA26	3.85	3.86	4.60	-0.24	10.98
SIE4x4	20.14	20.14	22.78	3.97	41.18
ALKBDE10	3.55	4.10	6.03	-1.98	13.98
YBDE18	-1.77	3.39	4.10	-8.18	5.08
AL2X6	0.07	0.55	0.74	-1.42	0.96
HEAVYSB11	-4.55	4.92	6.07	-10.51	2.01
NBPRC	-0.24	2.01	2.19	-3.15	3.30
ALK8	5.49	5.53	6.78	-0.16	11.56
RC21	4.47	4.72	5.28	-1.64	8.77
G2RC	-0.54	3.46	4.41	-10.86	7.70
BH76RC	-0.91	2.66	3.38	-8.49	4.12
FH51	0.49	2.32	3.20	-7.30	9.63
TAUT15	-0.22	1.33	1.60	-2.41	3.16
DC13	0.37	9.38	11.67	-23.24	17.74
MB16-43	0.09	13.92	18.11	-34.76	56.15
DARC	3.74	3.90	4.15	-1.14	5.41
RSE43	-1.90	1.90	2.16	-4.67	-0.56
BSR36	-2.74	2.74	3.48	-9.84	-0.58
CDIE20	1.06	1.07	1.18	-0.02	2.02
ISO34	-0.11	1.44	2.14	-3.59	9.07
ISOL24	-1.80	5.13	6.87	-10.83	18.84
C60ISO	-11.00	11.00	13.23	-23.23	-1.69
PArel	0.08	1.15	1.83	-3.03	6.03
BH76	-5.95	6.03	6.65	-12.47	2.31
BHPERI	-3.06	3.11	3.35	-5.66	0.74
BHDIV10	-3.05	4.04	4.58	-7.77	4.93
INV24	-1.49	2.06	4.39	-19.35	4.32
BHROT27	0.38	0.48	0.69	-0.47	1.71
PX13	-5.53	5.53	5.73	-8.49	-3.45
WCPT18	-3.38	3.47	4.41	-8.45	0.79
RG18	0.42	0.42	0.49	0.09	0.99
ADIM6	1.78	1.78	1.92	0.76	2.76
S22	1.65	1.65	1.82	0.40	2.90
S66	1.52	1.52	1.59	0.71	2.97
HEAVY28	1.11	1.11	1.15	0.21	1.57
WATER27	10.67	10.91	15.31	-3.23	36.02
CARBHB12	2.07	2.07	2.27	0.99	4.05
PNICO23	1.54	1.54	1.66	0.75	3.17
HAL59	1.44	1.44	1.63	0.29	4.34
AHB21	-1.84	1.84	1.91	-2.84	-1.12
CHB6	-1.70	1.73	1.92	-2.65	0.10
IL16	-1.74	1.74	1.80	-2.44	-0.83
IDISP	3.27	3.27	4.51	0.86	9.50
ICONF	-0.08	0.33	0.42	-0.71	0.84
ACONF	-0.30	0.30	0.35	-0.68	-0.10
Amino20x4	0.04	0.28	0.38	-0.94	1.22
PCONF21	-0.09	0.85	1.03	-1.49	1.99
MCONF	0.60	0.70	0.82	-0.61	1.47
SCONF	-0.01	0.56	0.86	-2.81	0.57
UPU23	-0.00	0.61	0.88	-3.04	1.25
BUT14DIOL	0.53	0.53	0.56	-0.08	0.92

Table S14: Statistical errors (in kcal/mol) of the 55 subsets in the GMTKN55 database[3] for TAO-B3LYP-D4.

	MSE	MAE	RMS	MIN	MAX
W4-11	1.23	5.11	6.47	-23.20	16.07
G21EA	-1.79	3.77	4.85	-13.96	7.60
G21IP	-0.28	4.59	5.61	-10.19	12.10
DIPCS10	-0.95	3.62	4.21	-6.97	5.47
PA26	2.85	2.89	3.64	-0.46	9.64
SIE4x4	19.99	19.99	22.68	3.79	41.13
ALKBDE10	3.53	4.10	6.11	-2.03	14.36
YBDE18	-1.99	3.46	4.22	-8.45	5.01
AL2X6	-0.60	0.60	0.88	-1.86	-0.01
HEAVYSB11	-4.88	5.14	6.28	-10.80	1.42
NBPRC	-0.10	1.69	1.93	-2.93	2.63
ALK8	0.51	1.80	2.45	-4.79	3.77
RC21	4.19	4.49	5.03	-1.64	8.36
G2RC	-0.45	3.41	4.37	-10.82	7.84
BH76RC	-0.92	2.66	3.38	-8.36	4.19
FH51	0.54	2.28	3.17	-7.25	9.62
TAUT15	-0.08	1.28	1.59	-2.34	3.27
DC13	1.04	8.45	10.57	-21.34	16.20
MB16-43	-3.62	15.48	19.50	-47.36	55.54
DARC	3.62	3.80	4.07	-1.29	5.32
RSE43	-1.96	1.96	2.22	-4.77	-0.73
BSR36	-1.89	1.89	2.32	-6.12	-0.38
CDIE20	1.16	1.16	1.27	0.11	2.11
ISO34	-0.17	1.46	2.14	-3.58	8.91
ISOL24	-1.89	4.77	6.41	-10.63	16.90
C60ISO	-12.66	12.66	14.89	-25.60	-2.32
PArel	0.16	1.12	1.85	-2.95	6.32
BH76	-5.89	5.97	6.59	-12.39	2.43
BHPERI	-3.01	3.05	3.29	-5.32	0.57
BHDIV10	-3.01	3.97	4.51	-7.59	4.80
INV24	-1.78	1.99	4.36	-19.43	2.02
BHROT27	0.37	0.48	0.69	-0.47	1.70
PX13	-5.41	5.41	5.60	-8.28	-3.28
WCPT18	-3.27	3.36	4.25	-8.18	0.79
RG18	0.38	0.38	0.43	0.08	0.88
ADIM6	1.38	1.38	1.47	0.61	2.06
S22	1.68	1.68	1.94	0.33	3.68
S66	1.41	1.41	1.51	0.61	3.04
HEAVY28	0.92	0.92	0.96	0.08	1.55
WATER27	9.84	10.09	13.98	-3.43	32.98
CARBHB12	1.89	1.89	2.10	0.88	3.89
PNICO23	1.38	1.38	1.48	0.67	2.72
HAL59	1.42	1.42	1.58	0.37	4.18
AHB21	-1.93	1.93	1.98	-2.82	-1.25
CHB6	-0.87	1.21	1.38	-2.18	1.03
IL16	-2.13	2.13	2.25	-3.44	-1.19
IDISP	2.78	2.78	3.62	0.94	7.54
ICONF	-0.13	0.32	0.41	-0.85	0.69
ACONF	-0.32	0.32	0.37	-0.71	-0.11
Amino20x4	0.04	0.29	0.40	-0.83	1.48
PCONF21	0.00	0.81	1.09	-1.73	1.96
MCONF	0.61	0.76	0.86	-0.71	1.43
SCONF	0.04	0.65	0.95	-3.05	0.71
UPU23	-0.02	0.60	0.86	-2.90	1.31
BUT14DIOL	0.72	0.72	0.74	0.02	1.03

Table S15: Statistical errors (in kcal/mol) of the 55 subsets in the GMTKN55 database[3] for TAO-PBE0.

	MSE	MAE	RMS	MIN	MAX
W4-11	1.13	4.99	6.47	-20.19	12.28
G21EA	-5.36	5.46	6.32	-11.80	1.25
G21IP	-2.61	4.77	5.77	-12.53	9.49
DIPCS10	-5.60	5.60	6.50	-9.63	-0.52
PA26	3.55	3.58	4.16	-0.44	9.79
SIE4x4	16.46	16.46	18.56	3.95	32.48
ALKBDE10	0.57	4.43	5.32	-5.78	12.00
YBDE18	0.16	1.51	1.90	-4.64	3.77
AL2X6	0.28	1.23	1.50	-1.54	2.92
HEAVYSB11	-2.81	3.80	4.37	-9.22	4.60
NBPRC	-1.06	3.84	4.32	-6.65	6.59
ALK8	7.21	7.29	10.10	-0.31	18.74
RC21	6.73	6.93	7.99	-1.05	15.60
G2RC	-4.03	7.55	9.54	-24.93	11.40
BH76RC	-0.58	2.64	3.59	-8.06	10.23
FH51	-1.53	3.37	4.41	-10.37	8.78
TAUT15	0.36	1.32	1.91	-2.79	3.96
DC13	-2.58	11.40	14.92	-37.14	23.54
MB16-43	13.48	20.32	25.14	-30.70	77.38
DARC	-4.19	4.19	5.27	-11.13	-0.99
RSE43	-1.73	1.73	1.94	-4.50	-0.46
BSR36	-6.81	6.81	7.97	-18.56	-2.46
CDIE20	1.45	1.46	1.70	-0.14	3.14
ISO34	-0.42	1.68	2.27	-5.10	3.41
ISOL24	-0.64	3.45	4.83	-10.39	10.24
C60ISO	-11.35	11.35	13.53	-23.67	-2.01
PArel	0.81	1.39	2.02	-2.62	6.32
BH76	-4.66	4.96	5.52	-14.76	3.59
BHPERI	-3.04	3.35	3.56	-5.57	2.15
BHDIV10	-4.69	5.19	5.80	-8.65	2.47
INV24	-2.20	2.50	4.64	-19.61	2.72
BHROT27	0.55	0.60	0.81	-0.33	1.86
PX13	-7.33	7.33	7.56	-11.43	-4.62
WCPT18	-5.10	5.10	5.85	-10.86	-1.39
RG18	0.19	0.23	0.32	-0.32	0.96
ADIM6	-1.62	1.62	1.86	-3.08	-0.35
S22	-0.88	1.37	2.14	-6.39	2.09
S66	-0.65	1.14	1.43	-4.21	2.05
HEAVY28	0.24	0.29	0.39	-0.22	0.89
WATER27	7.30	7.77	9.60	-6.36	24.81
CARBHB12	1.91	1.91	2.24	0.75	4.70
PNICO23	0.81	0.91	1.34	-1.11	4.74
HAL59	0.25	0.77	1.18	-3.56	3.80
AHB21	-2.39	2.39	2.65	-5.15	-0.19
CHB6	-1.36	1.42	1.77	-3.48	0.18
IL16	-0.81	1.03	1.36	-3.18	1.27
IDISP	1.37	7.03	7.82	-9.43	12.21
ICONF	0.17	0.27	0.41	-0.31	1.03
ACONF	0.32	0.32	0.36	0.11	0.67
Amino20x4	0.09	0.37	0.49	-1.10	1.52
PCONF21	-0.46	2.26	2.47	-3.33	3.48
MCONF	-0.90	1.04	1.20	-2.27	0.79
SCONF	0.05	0.21	0.29	-0.86	0.49
UPU23	1.06	1.48	2.06	-1.25	4.85
BUT14DIOL	0.17	0.19	0.25	-0.11	0.75

Table S16: Statistical errors (in kcal/mol) of the 55 subsets in the GMTKN55 database[3] for TAO-PBE0-D3(0).

	MSE	MAE	RMS	MIN	MAX
W4-11	1.33	5.11	6.56	-19.46	13.21
G21EA	-5.36	5.47	6.32	-11.80	1.34
G21IP	-2.61	4.78	5.78	-12.53	9.49
DIPCS10	-5.63	5.63	6.51	-9.62	-0.56
PA26	3.78	3.80	4.36	-0.29	9.98
SIE4x4	16.73	16.73	18.78	3.95	32.59
ALKBDE10	0.58	4.42	5.32	-5.77	12.00
YBDE18	1.62	1.84	2.02	-1.03	4.17
AL2X6	2.65	2.84	3.12	-0.56	4.14
HEAVYSB11	-1.30	2.34	3.19	-7.77	4.61
NBPRC	-2.47	5.11	5.44	-9.15	6.79
ALK8	8.89	8.89	12.05	0.28	21.90
RC21	7.73	7.99	9.10	-1.31	16.26
G2RC	-4.17	7.88	9.96	-26.53	11.99
BH76RC	-0.61	2.69	3.67	-8.07	10.31
FH51	-2.29	3.87	5.17	-13.22	8.77
TAUT15	0.12	1.29	1.73	-2.91	3.73
DC13	-3.43	11.68	15.00	-37.18	22.97
MB16-43	24.81	26.96	32.87	-18.72	93.29
DARC	-7.39	7.39	7.72	-12.36	-4.87
RSE43	-1.49	1.49	1.71	-4.22	-0.38
BSR36	-3.30	3.30	4.36	-13.02	-0.68
CDIE20	1.31	1.31	1.52	0.11	2.93
ISO34	-0.34	1.48	2.06	-5.18	3.67
ISOL24	0.33	3.00	4.16	-9.94	7.79
C60ISO	-11.52	11.52	13.74	-24.04	-1.97
PArel	0.80	1.33	1.97	-2.42	6.26
BH76	-5.00	5.27	5.84	-15.04	3.43
BHPERI	-4.96	5.26	5.65	-8.51	2.05
BHDIV10	-4.89	5.33	6.01	-9.26	2.20
INV24	-1.67	2.32	4.59	-19.81	2.73
BHROT27	0.57	0.58	0.79	-0.15	1.89
PX13	-7.42	7.42	7.68	-11.67	-4.75
WCPT18	-5.56	5.56	6.50	-12.17	-1.22
RG18	0.69	0.69	0.80	0.22	1.98
ADIM6	2.15	2.15	2.29	1.04	3.27
S22	1.74	1.74	1.88	0.65	3.49
S66	1.72	1.72	1.80	0.93	3.60
HEAVY28	1.01	1.01	1.05	0.48	1.58
WATER27	14.16	14.50	19.99	-4.60	46.51
CARBHB12	2.68	2.68	2.98	1.16	5.24
PNICO23	1.66	1.66	1.96	0.67	5.42
HAL59	1.32	1.32	1.57	0.32	4.52
AHB21	-2.90	2.90	3.04	-5.38	-1.73
CHB6	-1.75	1.75	1.99	-3.63	-0.74
IL16	-2.94	2.94	2.98	-4.08	-2.17
IDISP	1.30	2.00	2.46	-2.09	5.10
ICONF	0.18	0.30	0.45	-0.62	1.22
ACONF	-0.21	0.21	0.23	-0.43	-0.06
Amino20x4	0.14	0.32	0.41	-0.74	1.07
PCONF21	-0.22	0.83	0.95	-1.41	1.70
MCONF	0.74	0.77	0.90	-0.39	1.76
SCONF	0.14	0.62	0.80	-2.31	0.70
UPU23	-0.22	0.61	0.83	-2.69	1.02
BUT14DIOL	0.42	0.42	0.45	-0.14	0.81

Table S17: Statistical errors (in kcal/mol) of the 55 subsets in the GMTKN55 database[3] for TAO-PBE0-D3(BJ).

	MSE	MAE	RMS	MIN	MAX
W4-11	1.93	5.42	6.86	-18.93	16.93
G21EA	-5.36	5.46	6.32	-11.80	1.23
G21IP	-2.61	4.78	5.78	-12.53	9.49
DIPCS10	-5.60	5.60	6.49	-9.62	-0.50
PA26	3.88	3.90	4.45	-0.22	10.09
SIE4x4	16.70	16.70	18.75	4.05	32.51
ALKBDE10	0.88	4.51	5.47	-5.65	12.49
YBDE18	2.23	2.32	2.53	-0.46	4.79
AL2X6	3.71	3.71	4.09	0.08	5.40
HEAVYSB11	-0.31	2.12	2.86	-6.62	5.18
NBPRC	-2.70	5.25	5.78	-10.15	6.94
ALK8	10.47	10.47	13.75	0.24	23.83
RC21	7.95	8.18	9.40	-1.20	17.73
G2RC	-4.29	8.09	10.34	-28.78	11.80
BH76RC	-0.59	2.72	3.73	-8.23	10.46
FH51	-2.27	3.89	5.21	-12.50	8.85
TAUT15	0.28	1.32	1.86	-2.83	4.08
DC13	-3.88	12.45	15.84	-39.16	23.00
MB16-43	31.25	32.46	38.82	-14.92	109.81
DARC	-8.41	8.41	8.72	-13.65	-5.69
RSE43	-1.59	1.59	1.80	-4.23	-0.32
BSR36	-2.48	2.48	3.20	-9.24	-0.42
CDIE20	1.32	1.32	1.53	0.01	2.96
ISO34	-0.26	1.50	2.09	-5.16	4.00
ISOL24	0.74	3.17	4.44	-10.21	9.97
C60ISO	-11.06	11.06	13.33	-23.57	-1.72
PArel	0.80	1.34	1.97	-2.72	6.16
BH76	-5.06	5.35	5.91	-15.33	3.44
BHPERI	-5.49	5.79	6.25	-9.68	2.26
BHDIV10	-5.24	5.73	6.45	-10.04	2.40
INV24	-1.50	2.41	4.56	-19.44	3.95
BHROT27	0.57	0.60	0.81	-0.29	1.93
PX13	-7.70	7.70	7.97	-12.12	-5.00
WCPT18	-5.71	5.71	6.66	-12.33	-1.40
RG18	0.64	0.64	0.74	0.19	1.59
ADIM6	1.87	1.87	1.99	0.93	2.77
S22	1.74	1.74	1.88	0.58	3.47
S66	1.63	1.63	1.69	0.91	3.57
HEAVY28	1.13	1.13	1.18	0.43	1.77
WATER27	12.84	13.25	18.00	-5.56	42.46
CARBHB12	2.70	2.70	3.00	1.27	5.29
PNICO23	2.08	2.08	2.41	0.86	6.40
HAL59	1.52	1.52	1.77	0.31	4.55
AHB21	-2.94	2.94	3.11	-5.80	-1.48
CHB6	-1.98	1.98	2.24	-3.97	-0.81
IL16	-2.75	2.75	2.85	-4.29	-1.82
IDISP	0.93	1.62	2.21	-1.89	4.78
ICONF	0.13	0.28	0.40	-0.61	1.11
ACONF	-0.25	0.25	0.28	-0.49	-0.08
Amino20x4	0.13	0.36	0.45	-0.81	1.19
PCONF21	-0.25	0.82	0.96	-1.76	1.55
MCONF	0.68	0.75	0.88	-0.46	1.75
SCONF	0.15	0.62	0.80	-2.35	0.69
UPU23	-0.06	0.52	0.75	-2.45	1.14
BUT14DIOL	0.45	0.45	0.48	-0.12	0.87

Table S18: Statistical errors (in kcal/mol) of the 55 subsets in the GMTKN55 database[3] for TAO-PBE0-D4.

	MSE	MAE	RMS	MIN	MAX
W4-11	1.87	5.36	6.79	-18.55	16.97
G21EA	-5.19	5.29	6.17	-11.74	1.29
G21IP	-2.54	4.72	5.71	-12.53	9.49
DIPCS10	-5.29	5.29	6.17	-9.62	-0.29
PA26	3.36	3.43	3.97	-0.89	9.37
SIE4x4	16.62	16.62	18.69	3.96	32.48
ALKBDE10	0.90	4.51	5.52	-5.65	12.73
YBDE18	2.20	2.31	2.50	-0.60	4.71
AL2X6	3.53	3.53	3.90	0.01	5.14
HEAVYSB11	-0.36	2.12	2.86	-6.58	5.18
NBPRC	-2.67	5.09	5.69	-10.18	6.85
ALK8	8.19	8.19	10.72	0.11	19.28
RC21	7.83	8.05	9.28	-1.20	17.70
G2RC	-4.25	8.05	10.30	-28.91	11.76
BH76RC	-0.59	2.71	3.72	-8.17	10.51
FH51	-2.26	3.86	5.18	-12.44	8.85
TAUT15	0.35	1.30	1.88	-2.78	4.14
DC13	-3.56	12.91	16.39	-38.43	26.63
MB16-43	30.50	31.83	38.25	-13.75	111.40
DARC	-8.59	8.59	8.90	-13.81	-5.90
RSE43	-1.63	1.63	1.84	-4.29	-0.40
BSR36	-1.96	1.96	2.48	-7.01	-0.27
CDIE20	1.36	1.36	1.58	-0.01	3.00
ISO34	-0.29	1.50	2.10	-5.18	3.93
ISOL24	0.74	3.20	4.60	-10.68	10.55
C60ISO	-12.02	12.02	14.28	-24.96	-2.07
PArel	0.84	1.35	1.99	-2.64	6.31
BH76	-5.04	5.33	5.88	-15.26	3.50
BHPERI	-5.51	5.82	6.29	-9.70	2.32
BHDIV10	-5.26	5.73	6.45	-10.06	2.34
INV24	-1.64	2.32	4.52	-19.44	2.77
BHROT27	0.57	0.60	0.81	-0.29	1.93
PX13	-7.65	7.65	7.91	-12.02	-4.90
WCPT18	-5.67	5.67	6.60	-12.22	-1.40
RG18	0.62	0.62	0.72	0.18	1.55
ADIM6	1.64	1.64	1.73	0.84	2.38
S22	1.79	1.79	1.97	0.53	3.48
S66	1.59	1.59	1.67	0.87	3.66
HEAVY28	1.08	1.08	1.13	0.37	1.74
WATER27	12.37	12.79	17.24	-5.66	40.97
CARBHB12	2.62	2.62	2.92	1.22	5.23
PNICO23	2.02	2.02	2.35	0.80	6.28
HAL59	1.55	1.55	1.79	0.35	4.50
AHB21	-3.01	3.01	3.17	-5.90	-1.70
CHB6	-1.61	1.61	1.80	-2.86	-0.42
IL16	-3.02	3.02	3.15	-4.80	-1.95
IDISP	0.63	1.71	2.23	-3.25	4.14
ICONF	0.11	0.27	0.38	-0.57	1.05
ACONF	-0.26	0.26	0.29	-0.50	-0.09
Amino20x4	0.14	0.35	0.44	-0.72	1.08
PCONF21	-0.19	0.78	0.88	-1.32	1.56
MCONF	0.69	0.78	0.90	-0.47	1.74
SCONF	0.19	0.69	0.87	-2.50	0.81
UPU23	-0.07	0.51	0.74	-2.37	1.15
BUT14DIOL	0.55	0.55	0.57	-0.07	0.84

Table S19: Statistical errors (in kcal/mol) of the 55 subsets in the GMTKN55 database[3] for TAO-BHHLYP.

	MSE	MAE	RMS	MIN	MAX
W4-11	-14.33	15.32	19.32	-56.23	13.00
G21EA	-10.74	10.84	12.04	-22.01	1.25
G21IP	-5.83	7.17	8.30	-15.65	8.30
DIPCS10	-10.87	11.55	13.20	-22.78	3.36
PA26	5.01	5.02	5.86	-0.13	12.93
SIE4x4	10.61	10.61	12.50	2.18	23.92
ALKBDE10	-6.58	6.76	8.20	-17.61	0.91
YBDE18	-4.01	4.01	4.51	-7.97	-1.23
AL2X6	-1.66	2.33	2.82	-5.07	2.01
HEAVYSB11	-3.81	5.93	6.95	-12.11	10.77
NBPRC	0.50	3.32	4.19	-7.03	7.54
ALK8	5.19	5.19	6.66	0.88	14.16
RC21	3.89	4.21	5.07	-3.12	11.96
G2RC	-5.11	6.42	7.84	-17.04	5.78
BH76RC	-1.72	4.18	5.13	-13.25	8.11
FH51	-1.74	3.01	3.76	-9.70	6.37
TAUT15	-0.19	0.94	1.30	-2.78	1.74
DC13	-2.35	11.51	13.22	-21.11	17.74
MB16-43	-18.65	30.31	41.12	-109.93	57.27
DARC	1.47	2.76	3.11	-5.81	3.98
RSE43	-0.78	0.82	1.04	-2.04	0.50
BSR36	-9.45	9.45	11.25	-27.50	-3.02
CDIE20	1.10	1.11	1.36	-0.08	2.77
ISO34	0.16	1.25	2.03	-5.62	8.22
ISOL24	-1.48	4.82	6.71	-16.96	17.81
C60ISO	-4.69	5.72	7.45	-13.10	4.12
PArel	0.71	1.40	2.02	-2.08	6.28
BH76	-0.96	2.51	3.16	-9.24	7.79
BHPERI	3.47	3.47	3.64	1.28	5.22
BHDIV10	1.74	2.16	2.60	-1.43	5.44
INV24	-0.96	2.54	4.23	-13.00	11.80
BHROT27	0.64	0.74	1.03	-0.43	2.54
PX13	0.73	1.19	1.45	-1.35	3.02
WCPT18	2.02	2.64	3.39	-1.06	6.25
RG18	0.46	0.46	0.58	0.10	1.68
ADIM6	-0.57	0.62	0.78	-1.49	0.14
S22	-0.10	1.31	1.80	-4.74	3.30
S66	0.13	0.93	1.18	-2.61	3.20
HEAVY28	0.48	0.48	0.53	0.14	0.95
WATER27	10.47	10.47	13.98	1.16	32.99
CARBHB12	1.69	1.69	1.90	0.88	3.98
PNICO23	0.56	0.64	0.74	-0.81	1.74
HAL59	0.22	0.54	0.68	-2.25	1.36
AHB21	-2.84	2.84	3.11	-7.04	-1.05
CHB6	-2.52	2.52	2.83	-4.46	-0.54
IL16	-0.94	1.15	1.43	-3.46	1.21
IDISP	2.56	9.45	11.48	-15.15	20.15
ICONF	-0.00	0.47	0.60	-1.31	1.08
ACONF	0.29	0.29	0.34	0.08	0.59
Amino20x4	0.05	0.36	0.47	-1.03	1.42
PCONF21	-0.01	1.28	1.44	-1.76	2.25
MCONF	-0.72	0.85	0.96	-1.78	0.55
SCONF	-0.40	0.51	0.56	-0.77	0.86
UPU23	0.98	1.24	1.69	-1.02	3.80
BUT14DIOL	0.16	0.17	0.21	-0.12	0.46

Table S20: Statistical errors (in kcal/mol) of the 55 subsets in the GMTKN55 database[3] for TAO-BHHLYP-D3(0).

	MSE	MAE	RMS	MIN	MAX
W4-11	-14.05	15.15	19.14	-56.17	14.00
G21EA	-10.74	10.85	12.04	-22.01	1.38
G21IP	-5.83	7.18	8.31	-15.68	8.31
DIPCS10	-10.91	11.54	13.22	-22.75	3.16
PA26	5.31	5.32	6.14	-0.13	13.17
SIE4x4	10.94	10.94	12.71	3.08	24.08
ALKBDE10	-6.56	6.74	8.18	-17.61	0.91
YBDE18	-2.07	2.13	2.80	-6.78	0.46
AL2X6	1.38	1.38	1.66	0.59	3.33
HEAVYSB11	-1.82	4.02	5.21	-10.14	10.79
NBPRC	-1.37	4.06	4.78	-8.29	7.82
ALK8	7.51	7.51	9.25	0.84	15.95
RC21	5.19	5.36	6.22	-1.26	12.76
G2RC	-5.30	6.81	8.27	-19.06	6.68
BH76RC	-1.75	4.22	5.19	-13.24	8.11
FH51	-2.75	3.58	4.67	-14.52	5.40
TAUT15	-0.50	0.92	1.25	-2.95	1.40
DC13	-3.36	10.00	11.83	-19.34	12.31
MB16-43	-3.39	27.58	36.94	-99.19	71.03
DARC	-2.61	2.61	3.03	-7.30	-1.64
RSE43	-0.45	0.60	0.81	-1.72	0.78
BSR36	-5.14	5.14	7.00	-21.12	-0.81
CDIE20	0.89	0.93	1.15	-0.36	2.45
ISO34	0.26	0.98	1.66	-2.11	7.68
ISOL24	-0.17	2.96	4.07	-8.20	10.80
C60ISO	-5.00	5.83	7.67	-13.70	3.15
PArel	0.69	1.39	2.02	-1.96	6.21
BH76	-1.42	2.76	3.37	-9.49	7.66
BHPERI	0.96	1.59	2.09	-2.10	5.40
BHDIV10	1.42	1.99	2.38	-1.59	4.99
INV24	-0.26	2.58	4.30	-13.29	11.75
BHROT27	0.67	0.75	1.03	-0.47	2.67
PX13	0.64	1.22	1.48	-1.31	3.19
WCPT18	1.40	3.35	3.69	-2.68	6.22
RG18	1.05	1.05	1.21	0.29	2.71
ADIM6	3.70	3.70	3.96	1.72	5.78
S22	3.01	3.01	3.24	0.90	5.11
S66	2.91	2.91	3.03	1.44	5.17
HEAVY28	1.56	1.56	1.59	0.67	2.16
WATER27	18.64	18.64	26.52	1.74	63.54
CARBHB12	2.65	2.65	2.79	1.44	4.70
PNICO23	1.67	1.67	1.76	0.80	3.06
HAL59	1.62	1.62	1.72	0.70	3.52
AHB21	-3.51	3.51	3.66	-7.17	-2.38
CHB6	-3.14	3.14	3.28	-4.74	-1.98
IL16	-3.65	3.65	3.71	-5.01	-2.70
IDISP	2.41	4.44	5.44	-6.10	9.34
ICONF	0.01	0.59	0.74	-1.14	1.39
ACONF	-0.35	0.35	0.40	-0.74	-0.11
Amino20x4	0.13	0.30	0.41	-0.75	1.09
PCONF21	0.29	1.66	2.02	-3.24	3.29
MCONF	1.18	1.20	1.33	-0.39	2.00
SCONF	-0.21	0.34	0.55	-1.71	0.40
UPU23	-0.49	1.17	1.60	-5.42	2.04
BUT14DIOL	0.45	0.45	0.48	-0.05	0.71

Table S21: Statistical errors (in kcal/mol) of the 55 subsets in the GMTKN55 database[3] for TAO-BHHLYP-D3(BJ).

	MSE	MAE	RMS	MIN	MAX
W4-11	-12.98	14.44	18.39	-55.64	15.82
G21EA	-10.75	10.84	12.04	-22.01	1.13
G21IP	-5.83	7.18	8.30	-15.64	8.30
DIPCS10	-10.86	11.53	13.19	-22.71	3.34
PA26	5.47	5.47	6.28	0.01	13.33
SIE4x4	10.92	10.92	12.68	2.87	23.95
ALKBDE10	-5.95	6.20	7.75	-16.89	1.25
YBDE18	-0.85	1.72	2.41	-6.61	2.42
AL2X6	3.76	3.76	3.86	2.50	4.93
HEAVYSB11	0.33	2.84	4.43	-7.16	11.90
NBPRC	-1.88	4.35	4.73	-5.95	8.04
ALK8	11.38	11.38	13.82	0.59	21.83
RC21	5.63	5.76	6.77	-1.05	15.29
G2RC	-5.45	7.04	8.79	-22.95	6.59
BH76RC	-1.71	4.18	5.15	-13.20	8.09
FH51	-2.77	3.56	4.64	-13.31	5.55
TAUT15	-0.27	0.92	1.27	-2.80	1.92
DC13	-4.32	8.95	11.52	-23.27	11.14
MB16-43	11.28	29.82	36.22	-81.56	76.44
DARC	-4.46	4.46	4.76	-9.57	-3.45
RSE43	-0.61	0.69	0.90	-1.86	0.67
BSR36	-3.63	3.63	4.96	-14.73	-0.32
CDIE20	0.91	0.94	1.17	-0.30	2.52
ISO34	0.41	1.03	1.68	-2.24	7.42
ISOL24	0.54	3.21	4.25	-7.51	9.73
C60ISO	-4.20	5.64	7.14	-12.84	4.24
PArel	0.69	1.30	1.94	-1.73	6.05
BH76	-1.55	2.79	3.39	-9.40	7.57
BHPERI	-0.04	1.82	2.20	-3.45	5.05
BHDIV10	0.79	1.78	2.32	-2.29	5.33
INV24	0.01	2.71	4.33	-12.53	11.84
BHROT27	0.68	0.78	1.07	-0.42	2.67
PX13	0.16	1.11	1.35	-1.70	2.79
WCPT18	1.14	3.49	3.73	-3.18	6.21
RG18	1.01	1.01	1.18	0.27	2.45
ADIM6	3.44	3.44	3.67	1.64	5.21
S22	3.04	3.04	3.30	0.91	5.32
S66	2.83	2.83	2.94	1.41	5.17
HEAVY28	1.84	1.84	1.90	0.62	2.59
WATER27	17.46	17.46	24.69	1.62	58.52
CARBHB12	2.72	2.72	2.86	1.58	4.83
PNICO23	2.37	2.37	2.46	1.50	3.63
HAL59	2.03	2.03	2.14	0.84	4.01
AHB21	-3.61	3.61	3.76	-7.24	-2.60
CHB6	-3.84	3.84	3.99	-5.60	-2.48
IL16	-3.49	3.49	3.57	-5.01	-2.23
IDISP	1.79	2.81	4.04	-3.08	8.83
ICONF	-0.07	0.57	0.69	-1.33	1.19
ACONF	-0.45	0.45	0.50	-0.85	-0.18
Amino20x4	0.11	0.32	0.42	-0.84	1.14
PCONF21	0.25	1.45	1.82	-2.75	3.15
MCONF	1.16	1.19	1.34	-0.41	2.03
SCONF	-0.21	0.31	0.56	-1.89	0.26
UPU23	-0.28	1.04	1.48	-5.32	1.98
BUT14DIOL	0.53	0.53	0.55	-0.03	0.76

Table S22: Statistical errors (in kcal/mol) of the 55 subsets in the GMTKN55 database[3] for TAO-BHHLYP-D4.

	MSE	MAE	RMS	MIN	MAX
W4-11	-13.27	14.58	18.51	-55.70	14.91
G21EA	-10.51	10.61	11.83	-21.94	1.29
G21IP	-5.74	7.09	8.21	-15.49	8.37
DIPCS10	-10.48	11.21	12.81	-21.68	3.67
PA26	4.75	4.76	5.56	-0.14	12.37
SIE4x4	10.80	10.80	12.62	2.55	23.92
ALKBDE10	-5.99	6.23	7.72	-16.86	1.23
YBDE18	-1.16	1.83	2.53	-6.81	2.22
AL2X6	3.34	3.34	3.45	2.11	4.49
HEAVYSB11	0.29	2.75	4.36	-7.05	11.74
NBPRC	-1.69	3.99	4.42	-5.85	7.85
ALK8	6.55	6.55	7.77	0.38	14.62
RC21	5.31	5.47	6.44	-1.35	14.82
G2RC	-5.34	6.91	8.59	-22.46	6.78
BH76RC	-1.72	4.19	5.14	-13.17	8.11
FH51	-2.67	3.44	4.47	-12.55	5.53
TAUT15	-0.19	0.92	1.27	-2.76	1.94
DC13	-3.73	8.25	11.04	-21.49	10.77
MB16-43	8.40	28.47	35.14	-78.08	75.82
DARC	-4.29	4.29	4.60	-9.41	-3.21
RSE43	-0.66	0.73	0.95	-1.93	0.60
BSR36	-3.05	3.05	4.09	-11.82	-0.24
CDIE20	1.00	1.02	1.24	-0.20	2.60
ISO34	0.35	1.04	1.66	-2.23	7.45
ISOL24	0.35	3.10	3.97	-7.48	7.92
C60ISO	-5.56	6.03	8.11	-14.79	1.75
PArel	0.75	1.34	2.02	-1.72	6.26
BH76	-1.46	2.74	3.34	-9.31	7.60
BHPERI	0.18	1.73	2.12	-3.69	4.96
BHDIV10	0.89	1.77	2.29	-2.22	5.26
INV24	-0.24	2.56	4.24	-12.65	11.82
BHROT27	0.66	0.76	1.05	-0.42	2.64
PX13	0.34	1.12	1.37	-1.62	2.91
WCPT18	1.30	3.34	3.64	-2.76	6.24
RG18	1.02	1.02	1.19	0.26	2.42
ADIM6	3.25	3.25	3.47	1.55	4.91
S22	3.17	3.17	3.50	0.85	6.31
S66	2.84	2.84	2.97	1.34	5.26
HEAVY28	1.88	1.88	1.95	0.54	2.72
WATER27	16.51	16.51	23.25	1.55	54.79
CARBHB12	2.61	2.61	2.75	1.54	4.71
PNICO23	2.32	2.32	2.39	1.36	3.50
HAL59	2.13	2.13	2.25	0.94	4.48
AHB21	-3.64	3.64	3.79	-7.29	-2.71
CHB6	-3.19	3.19	3.28	-4.12	-2.15
IL16	-3.72	3.72	3.86	-5.76	-2.27
IDISP	1.48	2.23	3.52	-2.24	8.12
ICONF	-0.12	0.58	0.70	-1.36	1.09
ACONF	-0.45	0.45	0.50	-0.87	-0.18
Amino20x4	0.11	0.33	0.43	-0.74	1.22
PCONF21	0.35	1.70	2.02	-3.01	3.33
MCONF	1.24	1.31	1.46	-0.44	2.13
SCONF	-0.21	0.36	0.60	-2.02	0.27
UPU23	-0.34	1.11	1.60	-5.69	2.14
BUT14DIOL	0.65	0.65	0.68	0.04	0.97

Table S23: Statistical errors (in kcal/mol) of the 55 subsets in the GMTKN55 database[3] for TAO-B97X-D4.

	MSE	MAE	RMS	MIN	MAX
W4-11	-4.44	5.05	6.64	-24.10	9.26
G21EA	-0.41	2.81	3.60	-7.32	7.85
G21IP	3.64	5.59	6.69	-11.57	13.30
DIPCS10	5.80	6.07	7.94	-1.34	15.44
PA26	6.21	6.21	6.68	2.12	13.37
SIE4x4	14.82	14.82	16.88	3.78	29.65
ALKBDE10	-0.65	5.83	6.49	-8.23	12.60
YBDE18	-0.16	2.12	2.51	-5.40	4.08
AL2X6	-0.40	0.61	1.05	-2.48	0.44
HEAVYSB11	-1.01	2.79	3.63	-8.55	4.26
NBPRC	0.00	1.60	1.99	-2.34	4.51
ALK8	3.95	3.95	5.31	0.31	8.74
RC21	4.70	4.82	5.53	-0.65	11.15
G2RC	-2.74	4.67	6.18	-19.02	7.58
BH76RC	-0.46	2.29	2.61	-4.37	4.78
FH51	-0.97	2.04	2.85	-6.88	7.51
TAUT15	-0.12	0.99	1.18	-2.18	1.82
DC13	-0.97	7.59	10.14	-23.59	17.21
MB16-43	0.61	14.17	18.19	-30.66	62.84
DARC	-3.04	3.04	3.63	-8.07	-1.02
RSE43	-1.45	1.45	1.71	-3.82	-0.33
BSR36	-2.92	2.92	3.11	-6.54	-1.36
CDIE20	1.27	1.27	1.50	-0.05	2.95
ISO34	-0.20	1.15	1.53	-3.30	4.68
ISOL24	-0.28	2.34	3.43	-8.51	6.37
C60ISO	-9.78	9.78	12.01	-21.46	-1.29
PArel	0.68	0.94	1.61	-0.80	5.80
BH76	-2.34	2.53	2.99	-6.58	1.98
BHPERI	-1.46	2.06	2.41	-4.54	3.27
BHDIV10	-1.74	2.43	2.76	-4.32	3.43
INV24	-1.03	2.29	4.15	-16.72	6.25
BHROT27	0.48	0.52	0.74	-0.12	1.99
PX13	-2.43	2.43	2.75	-3.97	-0.13
WCPT18	-0.83	2.00	2.31	-4.67	2.38
RG18	0.02	0.08	0.09	-0.14	0.16
ADIM6	-0.90	0.90	1.00	-1.59	-0.31
S22	0.18	0.33	0.46	-0.47	1.29
S66	-0.10	0.38	0.53	-1.26	1.47
HEAVY28	0.08	0.24	0.29	-0.59	0.50
WATER27	-1.64	2.68	4.19	-12.79	2.24
CARBHB12	1.15	1.15	1.48	0.27	3.28
PNICO23	0.43	0.54	0.69	-0.65	1.79
HAL59	-0.01	0.36	0.49	-1.18	1.57
AHB21	-1.39	1.41	1.68	-3.56	0.11
CHB6	-0.12	0.92	1.13	-1.34	2.17
IL16	-0.93	1.10	1.47	-3.36	0.81
IDISP	1.18	1.92	2.39	-1.94	4.14
ICONF	-0.02	0.30	0.44	-0.79	1.02
ACONF	-0.02	0.03	0.05	-0.12	0.04
Amino20x4	-0.02	0.23	0.30	-0.72	0.99
PCONF21	-0.21	0.46	0.57	-1.18	0.56
MCONF	-0.06	0.25	0.31	-0.76	0.53
SCONF	-0.12	0.30	0.48	-1.67	0.27
UPU23	0.61	0.76	0.94	-0.87	2.03
BUT14DIOL	0.14	0.14	0.17	-0.02	0.46

Table S24: Statistical errors (in kcal/mol) of the 55 subsets in the GMTKN55 database[3] for KS-ωB97X.

	MSE	MAE	RMS	MIN	MAX
W4-11	-2.76	3.27	6.74	-48.40	3.85
G21EA	-1.32	2.30	2.73	-5.07	4.75
G21IP	-0.26	2.97	3.89	-10.49	7.27
DIPCS10	-4.82	5.50	7.52	-16.66	2.19
PA26	2.71	2.71	3.18	0.46	6.46
SIE4x4	11.36	11.36	13.38	3.82	29.29
ALKBDE10	-1.83	4.22	5.75	-12.74	5.50
YBDE18	-1.88	2.05	2.50	-5.66	0.84
AL2X6	-3.31	3.31	3.49	-4.75	-1.59
HEAVYSB11	-2.38	3.13	3.39	-5.05	3.10
NBPRC	1.20	2.70	3.45	-3.60	8.28
ALK8	-2.34	2.48	3.33	-6.87	0.56
RC21	2.55	2.99	3.44	-2.38	6.31
G2RC	-2.31	4.11	5.00	-10.01	9.16
BH76RC	-0.10	1.70	2.02	-3.76	3.47
FH51	-1.08	2.20	2.88	-9.10	4.34
TAUT15	-0.41	0.87	1.04	-2.28	1.60
DC13	-1.49	6.81	8.63	-16.22	18.09
MB16-43	-37.17	38.65	45.66	-114.44	16.76
DARC	-0.85	1.28	1.94	-5.27	0.98
RSE43	-1.24	1.24	1.35	-2.77	-0.59
BSR36	-5.83	5.83	7.22	-19.37	-1.63
CDIE20	-0.02	0.65	0.86	-1.11	1.94
ISO34	-0.65	1.10	1.54	-3.61	2.53
ISOL24	-1.25	3.15	4.27	-11.98	8.14
C60ISO	14.16	14.16	15.52	4.77	22.80
PArel	0.28	0.82	1.25	-1.14	3.67
BH76	-0.57	1.97	2.31	-5.49	5.01
BHPERI	3.86	3.96	4.42	-1.35	9.84
BHDIV10	1.02	1.27	1.63	-0.74	3.69
INV24	-0.46	1.19	1.97	-5.68	6.25
BHROT27	0.28	0.41	0.56	-0.54	1.29
PX13	-1.05	1.23	1.59	-2.94	0.73
WCPT18	0.13	1.68	2.21	-2.63	6.70
RG18	-0.12	0.16	0.22	-0.68	0.19
ADIM6	-0.44	0.44	0.51	-0.86	-0.05
S22	-0.43	0.81	1.13	-3.20	1.39
S66	-0.13	0.53	0.67	-1.99	1.43
HEAVY28	0.25	0.25	0.36	-0.01	0.99
WATER27	5.53	5.53	7.56	0.56	18.09
CARBHB12	0.77	0.77	0.87	0.12	1.72
PNICO23	0.23	0.38	0.44	-0.70	0.77
HAL59	0.05	0.45	0.59	-2.05	0.94
AHB21	-0.82	0.82	0.88	-1.50	-0.23
CHB6	0.69	0.82	1.00	-0.38	1.60
IL16	1.04	1.04	1.12	0.22	1.68
IDISP	0.20	4.35	5.31	-9.88	6.51
ICONF	0.14	0.42	0.55	-0.80	1.15
ACONF	0.09	0.09	0.11	0.01	0.26
Amino20x4	-0.03	0.25	0.32	-0.95	0.85
PCONF21	0.17	0.82	0.97	-0.83	1.90
MCONF	-0.34	0.46	0.54	-1.07	0.54
SCONF	-0.23	0.26	0.28	-0.46	0.30
UPU23	1.16	1.17	1.52	-0.13	3.13
BUT14DIOL	0.12	0.13	0.15	-0.05	0.37

Table S25: Statistical errors (in kcal/mol) of the 55 subsets in the GMTKN55 database[3] for KS-ωB97X-D3.

	MSE	MAE	RMS	MIN	MAX
W4-11	-2.60	3.10	6.48	-48.72	3.86
G21EA	-1.31	2.22	2.63	-4.49	4.28
G21IP	-0.06	2.99	3.87	-10.83	7.04
DIPCS10	-4.59	5.40	7.21	-14.90	1.99
PA26	3.60	3.60	4.02	1.05	7.74
SIE4x4	12.32	12.32	14.29	3.85	30.82
ALKBDE10	-2.58	4.38	5.72	-12.66	6.58
YBDE18	-2.24	2.35	2.83	-5.92	0.60
AL2X6	-3.04	3.04	3.15	-4.35	-1.97
HEAVYSB11	-1.74	2.46	2.67	-4.49	2.41
NBPRC	1.16	1.92	2.62	-2.17	6.45
ALK8	-3.45	3.46	4.68	-8.66	0.04
RC21	2.85	3.04	3.49	-1.10	6.35
G2RC	-1.79	3.97	4.86	-9.58	10.86
BH76RC	-0.19	1.64	2.04	-4.46	4.15
FH51	-0.84	1.92	2.48	-6.45	4.09
TAUT15	-0.34	0.74	0.90	-1.87	1.24
DC13	-1.14	6.38	8.20	-14.38	16.91
MB16-43	-30.09	31.54	37.38	-94.66	10.42
DARC	-1.33	1.39	1.97	-5.13	0.24
RSE43	-1.24	1.24	1.39	-3.02	-0.36
BSR36	-3.89	3.89	4.69	-12.26	-1.25
CDIE20	0.12	0.57	0.83	-0.99	1.92
ISO34	-0.55	1.03	1.39	-3.16	2.39
ISOL24	-0.85	2.50	3.31	-8.90	6.72
C60ISO	12.54	12.54	13.59	4.59	19.33
PArel	0.32	0.75	1.13	-0.85	2.65
BH76	-1.09	1.94	2.35	-5.15	4.01
BHPERI	2.38	2.51	2.87	-1.62	6.61
BHDIV10	0.14	1.00	1.25	-1.66	2.66
INV24	-0.35	1.11	1.80	-4.92	5.55
BHROT27	0.23	0.40	0.53	-0.65	1.20
PX13	-2.29	2.29	2.49	-4.04	-0.68
WCPT18	-0.59	1.62	2.07	-3.68	5.07
RG18	-0.21	0.22	0.32	-0.87	0.02
ADIM6	0.33	0.33	0.40	0.03	0.65
S22	0.09	0.21	0.30	-0.49	0.98
S66	0.24	0.26	0.32	-0.30	1.15
HEAVY28	-0.11	0.19	0.23	-0.45	0.44
WATER27	1.84	1.87	2.54	-0.45	6.11
CARBHB12	0.59	0.59	0.70	0.10	1.43
PNICO23	-0.20	0.25	0.31	-0.74	0.19
HAL59	-0.35	0.41	0.49	-1.50	0.34
AHB21	-0.23	0.30	0.41	-1.04	0.28
CHB6	1.25	1.25	1.41	0.38	2.05
IL16	0.95	0.98	1.17	-0.10	2.33
IDISP	0.51	2.73	3.52	-5.67	5.52
ICONF	0.15	0.43	0.58	-1.02	1.35
ACONF	0.02	0.05	0.06	-0.07	0.14
Amino20x4	-0.05	0.24	0.31	-0.86	0.72
PCONF21	0.09	0.31	0.38	-0.62	0.73
MCONF	0.31	0.31	0.34	-0.11	0.59
SCONF	-0.23	0.26	0.35	-1.07	0.22
UPU23	0.63	0.77	0.90	-1.14	1.67
BUT14DIOL	0.09	0.11	0.15	-0.18	0.44

Table S26: Statistical errors (in kcal/mol) of the 55 subsets in the GMTKN55 database[3] for KS-ωB97X-D4.

	MSE	MAE	RMS	MIN	MAX
W4-11	-2.43	3.17	5.61	-36.96	4.38
G21EA	-0.94	2.22	2.75	-4.74	6.48
G21IP	1.74	3.40	4.32	-4.88	9.10
DIPCS10	0.89	3.92	4.70	-6.75	8.16
PA26	1.70	1.85	2.35	-0.86	5.58
SIE4x4	11.02	11.02	13.49	1.83	30.13
ALKBDE10	-3.22	4.88	6.50	-14.14	5.90
YBDE18	-1.53	1.84	2.24	-4.42	1.41
AL2X6	0.17	0.90	1.13	-1.93	1.49
HEAVYSB11	-3.92	3.92	4.08	-6.07	-2.43
NBPRC	-0.48	1.85	2.08	-2.48	3.72
ALK8	-0.27	2.90	3.56	-7.00	5.01
RC21	2.89	3.02	3.58	-0.80	7.94
G2RC	-1.73	4.01	4.83	-10.49	11.97
BH76RC	-0.34	1.79	2.07	-4.16	2.88
FH51	-0.69	2.09	2.67	-6.76	5.97
TAUT15	0.13	0.43	0.57	-0.93	1.14
DC13	-3.11	5.87	7.10	-11.12	12.04
MB16-43	-17.48	22.63	28.74	-84.46	44.21
DARC	-4.59	4.59	4.64	-6.38	-3.68
RSE43	-0.75	0.76	0.92	-2.25	0.17
BSR36	-1.09	1.09	1.19	-2.38	-0.16
CDIE20	-0.13	0.64	0.81	-0.89	1.76
ISO34	-0.49	1.02	1.28	-2.22	2.41
ISOL24	0.47	2.68	3.92	-13.15	6.91
C60ISO	13.59	13.59	14.79	4.81	21.51
PArel	0.31	0.62	0.87	-1.26	1.84
BH76	-1.26	1.79	2.15	-5.27	2.31
BHPERI	1.04	1.31	2.15	-0.53	8.31
BHDIV10	-0.47	1.21	1.52	-2.65	2.61
INV24	0.14	1.33	1.93	-5.08	5.76
BHROT27	0.13	0.33	0.43	-0.67	0.93
PX13	-3.49	3.49	3.73	-6.07	-1.09
WCPT18	-0.47	1.91	2.55	-3.77	7.48
RG18	0.02	0.05	0.06	-0.07	0.15
ADIM6	-0.14	0.14	0.16	-0.27	-0.05
S22	0.10	0.19	0.24	-0.26	0.57
S66	0.07	0.16	0.20	-0.27	0.71
HEAVY28	0.04	0.14	0.17	-0.36	0.31
WATER27	-0.50	1.23	1.69	-4.60	1.81
CARBHB12	0.30	0.32	0.41	-0.11	0.83
PNICO23	0.03	0.15	0.19	-0.21	0.47
HAL59	-0.18	0.29	0.43	-1.56	0.56
AHB21	-0.13	0.42	0.53	-1.19	0.69
CHB6	-1.59	1.80	2.08	-3.41	0.63
IL16	0.87	0.87	0.95	0.25	1.45
IDISP	-1.21	2.73	4.24	-9.73	2.88
ICONF	0.03	0.26	0.34	-0.33	0.75
ACONF	-0.03	0.03	0.04	-0.09	0.03
Amino20x4	-0.03	0.19	0.24	-0.62	0.47
PCONF21	0.11	0.21	0.27	-0.30	0.52
MCONF	0.10	0.23	0.26	-0.38	0.50
SCONF	-0.16	0.17	0.24	-0.67	0.04
UPU23	0.64	0.71	0.84	-0.57	1.54
BUT14DIOL	0.05	0.05	0.06	-0.06	0.16

Table S27: Statistical errors (in kcal/mol) of the 55 subsets in the GMTKN55 database[3] for TAO-ωB97X-D4.

	MSE	MAE	RMS	MIN	MAX
W4-11	-3.11	4.48	6.40	-29.03	9.88
G21EA	-0.48	2.85	3.62	-9.64	6.20
G21IP	3.98	6.30	7.86	-14.45	16.95
DIPCS10	7.17	7.17	8.67	0.48	15.44
PA26	6.38	6.38	6.83	2.67	12.89
SIE4x4	15.19	15.19	17.44	3.79	33.29
ALKBDE10	2.74	6.18	8.12	-6.50	18.19
YBDE18	0.28	1.80	2.17	-3.62	4.06
AL2X6	-0.74	0.74	1.30	-3.12	-0.08
HEAVYSB11	-0.83	4.46	5.29	-6.66	11.22
NBPRC	-1.20	2.82	3.02	-4.09	4.77
ALK8	9.37	9.37	14.77	0.18	31.94
RC21	6.17	6.22	7.06	-0.54	12.54
G2RC	-3.05	5.18	6.55	-18.62	7.20
BH76RC	-0.94	2.32	2.94	-6.37	3.56
FH51	-1.64	2.83	3.65	-10.07	5.20
TAUT15	-0.74	1.16	1.50	-3.26	0.90
DC13	-2.97	10.02	11.91	-21.05	19.09
MB16-43	0.46	16.55	20.95	-35.30	66.43
DARC	-6.42	6.42	6.73	-11.07	-4.03
RSE43	-1.32	1.32	1.52	-3.42	-0.28
BSR36	-2.31	2.31	2.45	-5.55	-1.06
CDIE20	0.90	1.00	1.47	-0.90	3.49
ISO34	-0.44	1.28	1.82	-4.62	3.92
ISOL24	0.46	3.01	4.00	-6.18	9.65
C60ISO	-8.62	8.62	10.63	-19.13	-1.21
PArel	0.46	0.88	1.34	-1.07	4.10
BH76	-1.80	2.57	2.99	-6.90	4.06
BHPERI	-0.31	1.86	2.50	-2.99	8.75
BHDIV10	-1.44	1.96	2.22	-3.89	1.34
INV24	-1.45	3.02	4.58	-16.45	5.31
BHROT27	0.32	0.49	0.68	-0.88	1.65
PX13	-3.87	3.87	4.12	-5.75	-1.32
WCPT18	-1.53	2.32	2.79	-5.63	2.69
RG18	0.01	0.09	0.11	-0.17	0.20
ADIM6	-0.68	0.68	0.76	-1.14	-0.21
S22	0.31	0.42	0.57	-0.49	1.38
S66	0.06	0.35	0.44	-0.94	1.15
HEAVY28	0.06	0.26	0.30	-0.55	0.50
WATER27	-2.08	3.32	5.17	-15.38	2.90
CARBHB12	1.58	1.58	1.97	0.37	4.49
PNICO23	0.20	0.34	0.42	-0.81	0.84
HAL59	-0.50	0.63	0.89	-2.64	0.78
AHB21	-1.75	1.75	1.90	-3.19	0.00
CHB6	-0.09	0.95	1.14	-1.23	2.20
IL16	-2.88	2.88	3.62	-7.74	-0.27
IDISP	0.01	1.76	2.43	-5.25	1.95
ICONF	-0.15	0.47	0.65	-1.72	1.03
ACONF	-0.25	0.25	0.27	-0.44	-0.09
Amino20x4	-0.08	0.26	0.33	-0.63	0.80
PCONF21	-0.24	0.48	0.62	-1.29	0.65
MCONF	0.17	0.36	0.44	-0.78	0.95
SCONF	-0.14	0.35	0.61	-1.89	0.36
UPU23	0.67	0.98	1.23	-1.43	2.72
BUT14DIOL	0.08	0.10	0.13	-0.15	0.29

Table S28: Optimized bond lengths (in Å) in EXTS,[5] obtained with TAO-DFT functionals.

Molecule	Experiment	TAO-B97-D4	TAO-B97X-D4	TAO-ωB97X-D4
H_2N^-	1.028	1.032	1.022	1.021
NO^- ($^3\Sigma^-$)	1.258	1.267	1.242	1.237
PO^- ($^3\Sigma^-$)	1.540	1.558	1.524	1.522
BeH^+ ($^1\Sigma^+$)	1.312	1.317	1.305	1.306
CH^+ ($^1\Sigma^+$)	1.131	1.146	1.129	1.132
CN^+ ($^1\Sigma^+$)	1.173	1.224	1.183	1.177
H_3^+	0.877	0.877	0.871	0.875
H_3O^+	0.976	0.980	0.971	0.974
HCO^+(CH)	1.097	1.097	1.089	1.092
HCO^+(CO)	1.105	1.111	1.095	1.095
MgH^+ ($^1\Sigma^+$)	1.652	1.664	1.645	1.641
NO^+ ($^1\Sigma^+$)	1.063	1.069	1.049	1.051
NS^+ ($^1\Sigma^+$)	1.440	1.441	1.416	1.413
OH^+ ($^3\Sigma^-$)	1.029	1.038	1.025	1.030
SiH^+ ($^1\Sigma^+$)	1.504	1.522	1.507	1.507
NH_4^+	1.021	1.024	1.017	1.018
AlCl ($^1\Sigma^+$)	2.130	2.162	2.143	2.138
AlF ($^1\Sigma^+$)	1.654	1.698	1.670	1.672
AlH ($^1\Sigma^+$)	1.648	1.669	1.648	1.642
AlN ($^3\Pi_i$)	1.786	1.811	1.898	1.789
BCl ($^1\Sigma^+$)	1.797	1.732	1.712	1.698
BeO ($^1\Sigma^+$)	1.331	1.349	1.322	1.322
BeS ($^1\Sigma^+$)	1.742	1.775	1.757	1.749
BF ($^1\Sigma^+$)	1.263	1.274	1.257	1.257
BF_3	1.307	1.323	1.305	1.306
BH ($^1\Sigma^+$)	1.232	1.241	1.225	1.222
BN ($^3\Pi$)	1.281	1.333	1.315	1.304
C_6H_6(CC)	1.390	1.396	1.385	1.382
C_6H_6(CH)	1.086	1.085	1.077	1.077
CCl_2O(CO)	1.177	1.183	1.168	1.170
CCl_2O(CCl)	1.737	1.752	1.732	1.727
CF_4	1.315	1.335	1.313	1.314
CH_2Cl_2(CCl)	1.766	1.775	1.760	1.754
CH_2Cl_2(CH)	1.080	1.087	1.078	1.078
CH_2F_2(CF)	1.351	1.369	1.348	1.348
CH_2F_2(CH)	1.084	1.095	1.085	1.086
CH_2O_2 (*trans*)(C=O)	1.201	1.204	1.190	1.191
CH_2O_2 (*trans*)(CH)	1.091	1.101	1.091	1.091

CH_2O_2 (*trans*)(CO)	1.340	1.352	1.332	1.331
CH_2O_2 (*trans*)(OH)	0.969	0.971	0.961	0.963
CH_3Cl(CH)	1.086	1.089	1.080	1.080
CH_3Cl(CCl)	1.778	1.784	1.772	1.764
CH_3F(CH)	1.086	1.094	1.084	1.084
CH_3F(CF)	1.383	1.394	1.376	1.376
CH_4	1.087	1.091	1.083	1.082
CHF_3(CF)	1.328	1.349	1.328	1.328
CHF_3(CH)	1.091	1.094	1.084	1.085
Cl_2 ($^1\Sigma_g^+$)	1.988	1.996	1.984	1.988
ClCN(CCl)	1.629	1.630	1.620	1.621
ClCN(CN)	1.160	1.166	1.146	1.144
ClF	1.628	1.654	1.621	1.624
CO ($^1\Sigma^+$)	1.128	1.136	1.119	1.119
CO_2	1.160	1.168	1.152	1.152
CS ($^1\Sigma^+$)	1.535	1.545	1.524	1.517
CS_2	1.553	1.558	1.545	1.542
F_2 ($^1\Sigma_g^+$)	1.412	1.413	1.371	1.375
F_2O	1.405	1.416	1.374	1.375
F_2S	1.587	1.623	1.591	1.591
F_2Si	1.590	1.632	1.604	1.605
F_3HSi(SiF)	1.562	1.596	1.572	1.573
F_3HSi(SiH)	1.447	1.460	1.447	1.447
H_2 ($^1\Sigma_g^+$)	0.741	0.740	0.736	0.738
H_2CCCH_2(CH)	1.076	1.086	1.078	1.078
H_2CCCH_2(CC)	1.308	1.307	1.295	1.294
H_2CS(CS)	1.611	1.613	1.598	1.593
H_2CS(CH)	1.086	1.092	1.082	1.082
H_2O	0.958	0.962	0.953	0.955
H_2S	1.336	1.345	1.333	1.332
H_2Si	1.514	1.528	1.512	1.508
HCCCN(CH)	1.062	1.066	1.058	1.060
HCCCN(C≡C)	1.206	1.212	1.195	1.193
HCCCN(CC)	1.376	1.365	1.368	1.372
HCCCN(C≡N)	1.161	1.169	1.149	1.146
HCCH(CH)	1.061	1.065	1.058	1.059
HCCH(CC)	1.203	1.206	1.191	1.189
HCl ($^1\Sigma^+$)	1.275	1.282	1.271	1.272
HCN(CH)	1.065	1.070	1.061	1.063
HCN(CN)	1.153	1.157	1.140	1.139
HCP(CH)	1.066	1.075	1.066	1.066

HCP(CP)	1.540	1.543	1.525	1.520
HF ($^1\Sigma^+$)	0.917	0.923	0.913	0.916
HNC(NH)	0.994	1.000	0.991	0.993
HNC(NC)	1.169	1.176	1.159	1.158
HOCl(OH)	0.964	0.969	0.958	0.960
HOCl(OCl)	1.689	1.707	1.675	1.672
LiCl ($^1\Sigma^+$)	2.021	2.030	2.020	2.021
LiF ($^1\Sigma^+$)	1.564	1.592	1.569	1.574
LiH ($^1\Sigma^+$)	1.596	1.595	1.585	1.583
MgO ($^1\Sigma^+$)	1.749	1.797	1.751	1.744
MgS ($^1\Sigma^+$)	2.143	2.216	2.184	2.167
N_2 ($^1\Sigma_g^+$)	1.098	1.103	1.086	1.086
N_2O(NN)	1.127	1.138	1.113	1.112
N_2O(NO)	1.185	1.187	1.172	1.173
NaCl ($^1\Sigma^+$)	2.361	2.392	2.376	2.381
NaF ($^1\Sigma^+$)	1.926	1.964	1.932	1.939
NaH ($^1\Sigma^+$)	1.887	1.898	1.888	1.886
NF ($^3\Sigma^-$)	1.317	1.325	1.299	1.297
NH ($^3\Sigma^-$)	1.036	1.044	1.031	1.033
NH_3	1.012	0.998	0.991	0.993
O_2 ($^3\Sigma_g^-$)	1.208	1.215	1.187	1.188
O_3	1.272	1.270	1.233	1.235
OCS(CO)	1.147	1.166	1.147	1.147
OCS(CS)	1.561	1.564	1.555	1.553
P_2 ($^1\Sigma_g^+$)	1.893	1.900	1.876	1.867
PF ($^3\Sigma^-$)	1.590	1.625	1.595	1.596
PH ($^3\Sigma^-$)	1.422	1.430	1.417	1.415
PH_3	1.413	1.421	1.409	1.407
PN ($^1\Sigma^+$)	1.491	1.495	1.471	1.467
S_2 ($^3\Sigma_g^-$)	1.889	1.912	1.885	1.876
S_2O(SS)	1.884	1.913	1.879	1.872
S_2O(SO)	1.456	1.474	1.448	1.447
SCS	1.553	1.558	1.545	1.542
SiO ($^1\Sigma^+$)	1.510	1.526	1.502	1.503
SiS ($^1\Sigma^+$)	1.929	1.948	1.927	1.922
SO ($^3\Sigma^-$)	1.481	1.500	1.473	1.470
SO_2	1.431	1.451	1.424	1.423
B_2H_6(BH $\times$2)	1.314	1.315	1.308	1.304
B_2H_6(BH $\times$4)	1.184	1.190	1.181	1.181
cyclopropane(CC)	1.501	1.511	1.496	1.491
cyclopropane(CH)	1.083	1.084	1.076	1.076

C_2^- ($^2\Sigma_g^+$)	1.268	1.281	1.259	1.253
NH^- ($^2\Pi_i$)	1.047	1.046	1.032	1.033
AlH^+ ($^2\Sigma^+$)	1.602	1.619	1.592	1.564
Cl_2^+ ($^2\Pi_{3/2g}$)	1.892	1.907	1.884	1.880
CO^+ ($^2\Sigma^+$)	1.115	1.121	1.100	1.100
HCl^+ ($^2\Pi_i$)	1.315	1.325	1.313	1.313
He_2^+ ($^2\Sigma_u^+$)	1.081	1.190	1.124	1.129
HF^+ ($^2\Pi_i$)	1.001	1.013	1.001	1.006
N_2^+ ($^2\Sigma_g^+$)	1.116	1.119	1.100	1.101
NH^+ ($^2\Pi_r$)	1.070	1.082	1.068	1.073
NH_3^+	1.014	1.025	1.017	1.019
O_2^+ ($^2\Pi_g$)	1.116	1.121	1.094	1.097
PF^+ ($^2\Pi_r$)	1.500	1.535	1.506	1.507
H_2O^+ ($^2B_1$)	0.999	1.006	0.995	0.998
AlS ($^2\Sigma^+$)	2.029	2.081	2.058	2.043
BeCl ($^2\Sigma^+$)	1.797	1.806	1.798	1.793
BeF ($^2\Sigma^+$)	1.361	1.378	1.363	1.363
BeH ($^2\Sigma^+$)	1.343	1.346	1.330	1.328
BO ($^2\Sigma^+$)	1.205	1.212	1.193	1.193
BS ($^2\Sigma^+$)	1.609	1.623	1.606	1.596
CCl ($^2\Pi_{1/2}$, $^2\Pi_{3/2}$)	1.645	1.657	1.631	1.621
CF ($^2\Pi_r$)	1.272	1.283	1.259	1.259
CH ($^2\Pi_r$)	1.120	1.131	1.114	1.113
CH_3	1.076	1.081	1.072	1.073
ClO ($^2\Pi_i$)	1.570	1.574	1.542	1.537
CN ($^2\Sigma^+$)	1.172	1.178	1.158	1.156
CP ($^2\Sigma^+$)	1.562	1.577	1.550	1.543
H_2N ($^2B_1$)	1.025	1.030	1.019	1.019
HO	0.970	0.976	0.965	0.968
HOO(OH)	0.971	0.980	0.966	0.969
HOO(OO)	1.331	1.332	1.304	1.300
MgCl ($^2\Sigma^+$)	2.199	2.228	2.208	2.207
MgF ($^2\Sigma^+$)	1.750	1.788	1.761	1.764
MgH ($^2\Sigma^+$)	1.730	1.749	1.712	1.709
NO ($^2\Pi_r$)	1.151	1.156	1.134	1.135
NO_2 ($^2A_1$)	1.195	1.202	1.179	1.179
NS ($^2\Pi_r$)	1.494	1.501	1.477	1.472
OP ($^2\Pi_r$)	1.474	1.494	1.465	1.464
SF ($^2\Pi_{3/2}$, $^2\Pi_{1/2}$)	1.601	1.625	1.596	1.596
SH ($^2\Pi_i$)	1.345	1.350	1.337	1.337
SiCl ($^2\Pi_r$)	2.058	2.077	2.061	2.058

SiF ($^2\Pi_r$)	1.601	1.643	1.613	1.613
SiH ($^2\Pi_r$)	1.520	1.536	1.519	1.515
SiN ($^2\Sigma^+$)	1.572	1.609	1.565	1.562
HCO(CH)	1.119	1.127	1.115	1.113
HCO(CO)	1.175	1.181	1.164	1.164

Table S29: Optimized bond lengths (in Å) in EXTS,[5] obtained with KS-DFT functionals.

Molecule	Experiment	KS-ωB97X-D4	KS-ωB97X-D3	KS-ωB97X-D
H_2N^-	1.028	1.028	1.025	1.026
NO^- ($^3\Sigma^-$)	1.258	1.254	1.253	1.253
PO^- ($^3\Sigma^-$)	1.540	1.533	1.534	1.533
BeH^+ ($^1\Sigma^+$)	1.312	1.314	1.314	1.312
CH^+ ($^1\Sigma^+$)	1.131	1.137	1.135	1.135
CN^+ ($^1\Sigma^+$)	1.173	1.171	1.170	1.170
H_3^+	0.877	0.882	0.878	0.877
H_3O^+	0.976	0.980	0.975	0.975
HCO^+(CH)	1.097	1.098	1.096	1.095
HCO^+(CO)	1.105	1.100	1.099	1.098
MgH^+ ($^1\Sigma^+$)	1.652	1.647	1.646	1.647
NO^+ ($^1\Sigma^+$)	1.063	1.055	1.054	1.053
NS^+ ($^1\Sigma^+$)	1.440	1.417	1.417	1.418
OH^+ ($^3\Sigma^-$)	1.029	1.036	1.029	1.028
SiH^+ ($^1\Sigma^+$)	1.504	1.510	1.510	1.510
NH_4^+	1.021	1.025	1.022	1.022
AlCl ($^1\Sigma^+$)	2.130	2.143	2.139	2.142
AlF ($^1\Sigma^+$)	1.654	1.675	1.673	1.673
AlH ($^1\Sigma^+$)	1.648	1.655	1.656	1.657
AlN ($^3\Pi_i$)	1.786	1.791	1.792	1.790
BCl ($^1\Sigma^+$)	1.797	1.709	1.717	1.720
BeO ($^1\Sigma^+$)	1.331	1.313	1.317	1.316
BeS ($^1\Sigma^+$)	1.742	1.726	1.732	1.735
BF ($^1\Sigma^+$)	1.263	1.259	1.263	1.260
BF_3	1.307	1.311	1.311	1.309
BH ($^1\Sigma^+$)	1.232	1.233	1.235	1.234
BN ($^3\Pi$)	1.281	1.311	1.315	1.315
C_6H_6(CC)	1.390	1.388	1.388	1.387
C_6H_6(CH)	1.086	1.083	1.083	1.082
CCl_2O(CO)	1.177	1.174	1.172	1.170
CCl_2O(CCl)	1.737	1.738	1.735	1.741
CF_4	1.315	1.320	1.318	1.317

CH_2Cl_2(CCl)	1.766	1.766	1.762	1.767
CH_2Cl_2(CH)	1.080	1.083	1.083	1.083
CH_2F_2(CF)	1.351	1.355	1.353	1.351
CH_2F_2(CH)	1.084	1.091	1.091	1.091
CH_2O_2 (*trans*)(C=O)	1.201	1.196	1.194	1.193
CH_2O_2 (*trans*)(CH)	1.091	1.097	1.097	1.096
CH_2O_2 (*trans*)(CO)	1.340	1.340	1.335	1.335
CH_2O_2 (*trans*)(OH)	0.969	0.969	0.966	0.965
CH_3Cl(CH)	1.086	1.086	1.085	1.085
CH_3Cl(CCl)	1.778	1.780	1.774	1.780
CH_3F(CH)	1.086	1.091	1.090	1.090
CH_3F(CF)	1.383	1.382	1.380	1.378
CH_4	1.087	1.089	1.088	1.088
CHF_3(CF)	1.328	1.334	1.332	1.331
CHF_3(CH)	1.091	1.091	1.090	1.090
Cl_2 ($^1\Sigma_g^+$)	1.988	1.989	1.978	1.987
ClCN(CCl)	1.629	1.630	1.626	1.626
ClCN(CN)	1.160	1.148	1.149	1.149
ClF	1.628	1.623	1.620	1.622
CO ($^1\Sigma^+$)	1.128	1.124	1.123	1.123
CO_2	1.160	1.158	1.156	1.155
CS ($^1\Sigma^+$)	1.535	1.522	1.523	1.525
CS_2	1.553	1.548	1.546	1.547
F_2 ($^1\Sigma_g^+$)	1.412	1.375	1.374	1.374
F_2O	1.405	1.379	1.376	1.378
F_2S	1.587	1.595	1.593	1.593
F_2Si	1.590	1.609	1.608	1.608
F_3HSi(SiF)	1.562	1.579	1.577	1.577
F_3HSi(SiH)	1.447	1.456	1.455	1.455
H_2 ($^1\Sigma_g^+$)	0.741	0.744	0.744	0.743
H_2CCCH_2(CH)	1.076	1.083	1.083	1.082
H_2CCCH_2(CC)	1.308	1.299	1.299	1.298
H_2CS(CS)	1.611	1.598	1.596	1.598
H_2CS(CH)	1.086	1.088	1.088	1.087
H_2O	0.958	0.961	0.957	0.957
H_2S	1.336	1.339	1.337	1.338
H_2Si	1.514	1.517	1.518	1.518
HCCCN(CH)	1.062	1.065	1.065	1.064
HCCCN(C≡C)	1.206	1.195	1.196	1.196
HCCCN(CC)	1.376	1.383	1.379	1.375
HCCCN(C≡N)	1.161	1.149	1.149	1.150

HCCH(CH)	1.061	1.064	1.064	1.063
HCCH(CC)	1.203	1.194	1.194	1.194
HCl ($^1\Sigma^+$)	1.275	1.277	1.275	1.277
HCN(CH)	1.065	1.068	1.067	1.067
HCN(CN)	1.153	1.144	1.143	1.143
HCP(CH)	1.066	1.072	1.072	1.071
HCP(CP)	1.540	1.523	1.523	1.525
HF ($^1\Sigma^+$)	0.917	0.921	0.917	0.917
HNC(NH)	0.994	0.998	0.996	0.996
HNC(NC)	1.169	1.162	1.163	1.163
HOCl(OH)	0.964	0.965	0.962	0.962
HOCl(OCl)	1.689	1.676	1.671	1.676
LiCl ($^1\Sigma^+$)	2.021	2.012	2.037	2.042
LiF ($^1\Sigma^+$)	1.564	1.570	1.585	1.586
LiH ($^1\Sigma^+$)	1.596	1.584	1.607	1.614
MgO ($^1\Sigma^+$)	1.749	1.726	1.724	1.726
MgS ($^1\Sigma^+$)	2.143	2.128	2.120	2.125
N_2 ($^1\Sigma_g^+$)	1.098	1.089	1.089	1.088
N_2O(NN)	1.127	1.115	1.115	1.115
N_2O(NO)	1.185	1.181	1.176	1.176
NaCl ($^1\Sigma^+$)	2.361	2.361	2.380	2.387
NaF ($^1\Sigma^+$)	1.926	1.930	1.939	1.941
NaH ($^1\Sigma^+$)	1.887	1.867	1.894	1.902
NF ($^3\Sigma^-$)	1.317	1.304	1.303	1.303
NH ($^3\Sigma^-$)	1.036	1.040	1.037	1.037
NH_3	1.012	0.999	0.996	0.997
O_2 ($^3\Sigma_g^-$)	1.208	1.196	1.193	1.194
O_3	1.272	1.238	1.234	1.236
OCS(CO)	1.147	1.151	1.150	1.149
OCS(CS)	1.561	1.562	1.558	1.559
P_2 ($^1\Sigma_g^+$)	1.893	1.864	1.864	1.869
PF ($^3\Sigma^-$)	1.590	1.600	1.599	1.599
PH ($^3\Sigma^-$)	1.422	1.424	1.422	1.423
PH_3	1.413	1.415	1.414	1.414
PN ($^1\Sigma^+$)	1.491	1.471	1.471	1.472
S_2 ($^3\Sigma_g^-$)	1.889	1.878	1.877	1.883
S_2O(SS)	1.884	1.866	1.865	1.872
S_2O(SO)	1.456	1.448	1.446	1.446
SCS	1.553	1.548	1.546	1.547
SiO ($^1\Sigma^+$)	1.510	1.506	1.503	1.504
SiS ($^1\Sigma^+$)	1.929	1.918	1.918	1.923

SO ($^3\Sigma^-$)	1.481	1.477	1.475	1.476
SO_2	1.431	1.427	1.425	1.426
B_2H_6(BH ×2)	1.314	1.312	1.316	1.315
B_2H_6(BH ×4)	1.184	1.188	1.189	1.189
cyclopropane(CC)	1.501	1.499	1.499	1.500
cyclopropane(CH)	1.083	1.082	1.081	1.081
C_2^- ($^2\Sigma_g^+$)	1.268	1.254	1.256	1.256
NH^- ($^2\Pi_i$)	1.047	1.039	1.038	1.038
AlH^+ ($^2\Sigma^+$)	1.602	1.606	1.603	1.603
Cl_2^+ ($^2\Pi_{3/2g}$)	1.892	1.882	1.877	1.887
CO^+ ($^2\Sigma^+$)	1.115	1.107	1.106	1.105
HCl^+ ($^2\Pi_i$)	1.315	1.319	1.316	1.318
He_2^+ ($^2\Sigma_u^+$)	1.081	1.132	1.135	1.136
HF^+ ($^2\Pi_i$)	1.001	1.009	1.004	1.003
N_2^+ ($^2\Sigma_g^+$)	1.116	1.102	1.102	1.101
NH^+ ($^2\Pi_r$)	1.070	1.078	1.072	1.072
NH_3^+	1.014	1.026	1.022	1.022
O_2^+ ($^2\Pi_g$)	1.116	1.101	1.099	1.099
PF^+ ($^2\Pi_r$)	1.500	1.511	1.510	1.510
H_2O^+ ($^2B_1$)	0.999	1.005	1.000	0.999
AlS ($^2\Sigma^+$)	2.029	2.028	2.024	2.028
BeCl ($^2\Sigma^+$)	1.797	1.796	1.808	1.809
BeF ($^2\Sigma^+$)	1.361	1.361	1.372	1.371
BeH ($^2\Sigma^+$)	1.343	1.341	1.351	1.350
BO ($^2\Sigma^+$)	1.205	1.198	1.200	1.199
BS ($^2\Sigma^+$)	1.609	1.599	1.606	1.607
CCl ($^2\Pi_{1/2}$, $^2\Pi_{3/2}$)	1.645	1.638	1.641	1.645
CF ($^2\Pi_r$)	1.272	1.267	1.268	1.266
CH ($^2\Pi_r$)	1.120	1.121	1.121	1.121
CH_3	1.076	1.079	1.079	1.078
ClO ($^2\Pi_i$)	1.570	1.560	1.555	1.557
CN ($^2\Sigma^+$)	1.172	1.159	1.158	1.158
CP ($^2\Sigma^+$)	1.562	1.542	1.543	1.545
H_2N ($^2B_1$)	1.025	1.027	1.025	1.025
HO	0.970	0.973	0.970	0.970
HOO(OH)	0.971	0.974	0.971	0.970
HOO(OO)	1.331	1.313	1.307	1.308
MgCl ($^2\Sigma^+$)	2.199	2.206	2.209	2.211
MgF ($^2\Sigma^+$)	1.750	1.765	1.766	1.765
MgH ($^2\Sigma^+$)	1.730	1.730	1.733	1.737
NO ($^2\Pi_r$)	1.151	1.141	1.140	1.140

NO_2 ($^2A_1$)	1.195	1.185	1.182	1.183
NS ($^2\Pi_r$)	1.494	1.480	1.478	1.480
OP ($^2\Pi_r$)	1.474	1.470	1.468	1.469
SF ($^2\Pi_{3/2}$, $^2\Pi_{1/2}$)	1.601	1.600	1.598	1.599
SH ($^2\Pi_i$)	1.345	1.343	1.341	1.342
SiCl ($^2\Pi_r$)	2.058	2.067	2.059	2.063
SiF ($^2\Pi_r$)	1.601	1.619	1.618	1.618
SiH ($^2\Pi_r$)	1.520	1.523	1.523	1.524
SiN ($^2\Sigma^+$)	1.572	1.559	1.557	1.558
HCO(CH)	1.119	1.122	1.121	1.121
HCO(CO)	1.175	1.170	1.169	1.168

Table S30: Singlet-triplet energy gap (in kcal/mol) of n-acene, obtained with spin-unrestricted TAO-B97-D4, TAO-B97X-D4, and TAO-ωB97X-D4. Here, the uncorrected experimental (EXP) data are taken from the works of Birks,[6] Schiedt *et al.*,[7] Sabbatini *et al.*[8] and Burgos *et al.*,[9] the DMRG data are taken from the work of Hachmann *et al.*,[10] and the ADMRPT2 data are taken from the work of Schriber *et al.*[11]

n-acene	TAO-B97-D4	TAO-B97X-D4	TAO-ωB97X-D4	DMRG/STO-3G	DMRG/cc-pVDZ	ADMRPT2	EXP
2	61.11	61.82	60.13	61.5	61.0	62.2	61.0
3	40.94	41.45	40.46	45.9	44.0	43.2	43.1
4	27.72	28.20	27.90	34.7	31.9	28.3	29.3
5	19.22	19.64	19.76	26.7	23.4	18.0	19.8
6	13.97	14.17	14.45	21.0	17.5	11.4	
7	10.79	10.68	10.96			7.7	
8	8.82	8.40	8.63	14.2			
9	7.50	6.87	7.03				
10	6.54	5.77	5.82	11.6			
11	5.78	4.95	4.96				
12	5.18	4.31	4.28	10.7			
13	4.68	3.81	3.75				
14	4.26	3.40	3.27				
15	3.92	3.07	2.94				
16	3.63	2.80	2.63				
17	3.38	2.57	2.48				
18	3.17	2.38	2.20				
19	2.98	2.21	2.01				
20	2.81	2.07	1.87				
21	2.66	1.95	1.80				
22	2.53	1.84	1.61				
23	2.41	1.74	1.61				
24	2.30	1.65	1.43				
25	2.20	1.57	1.45				
26	2.11	1.50	1.29				
27	2.02	1.43	1.25				
28	1.94	1.37	1.16				
29	1.87	1.32	1.12				
30	1.81	1.27	1.07				

Table S31: Singlet-triplet energy gap (in kcal/mol) of n-acene, obtained with spin-unrestricted KS-ωB97, KS-ωB97X, KS-ωB97X-D, KS-ωB97X-D3, and KS-ωB97X-D4. Here, the uncorrected experimental (EXP) data are taken from the works of Birks,[6] Schiedt *et al.*,[7] Sabbatini *et al.*[8] and Burgos *et al.*,[9] the DMRG data are taken from the work of Hachmann *et al.*,[10] and the ADMRPT2 data are taken from the work of Schriber *et al.*[11]

n-acene	KS-ωB97	KS-ωB97X	KS-ωB97X-D	KS-ωB97X-D3	KS-ωB97X-D4	DMRG/STO-3G	DMRG/cc-pVDZ	ADMRPT2	EXP
2	65.34	65.61	65.57	65.80	66.70	61.5	61.0	62.2	61.0
3	43.55	43.73	43.98	44.00	44.90	45.9	44.0	43.2	43.1
4	28.66	29.04	29.70	29.47	30.06	34.7	31.9	28.3	29.3
5	19.46	19.13	19.54	19.23	19.68	26.7	23.4	18.0	19.8
6	15.76	14.79	13.77	14.22	14.75	21.0	17.5	11.4	
7	14.84	13.39	11.46	12.37	12.99			7.7	
8	15.54	13.61	10.90	12.20	12.93	14.2			
9	17.33	14.88	11.35	13.05	13.93				
10	19.17	16.88	12.50	14.63	15.70	11.6			

Table S32: Vertical ionization potential (in eV) for the lowest singlet state of n-acene, obtained with spin-unrestricted TAO-B97-D4, TAO-B97X-D4, and TAO-ωB97X-D4. For comparison, the experimental (EXP) data are taken from the work of Malloci *et al.*,[12] and the CCSD(T)/CBS data are taken from the work of Deleuze *et al.*[13]

n-acene	TAO-B97-D4	TAO-B97X-D4	TAO-ωB97X-D4	CCSD(T)/CBS	EXP
2	8.03	7.85	7.91	8.24	8.14
3	7.27	7.07	7.14	7.47	7.44
4	6.75	6.54	6.62	6.95	6.97
5	6.37	6.16	6.25	6.57	6.59
6	6.11	5.89	5.98	6.43	
7	5.92	5.69	5.78		
8	5.78	5.54	5.62		
9	5.66	5.41	5.49		
10	5.56	5.31	5.39		
11	5.48	5.22	5.30		
12	5.40	5.15	5.23		
13	5.34	5.08	5.16		
14	5.28	5.03	5.10		
15	5.23	4.97	5.05		
16	5.19	4.93	5.00		
17	5.15	4.89	4.97		
18	5.11	4.85	4.93		
19	5.07	4.81	4.89		
20	5.04	4.78	4.86		
21	5.01	4.75	4.83		
22	4.99	4.73	4.80		
23	4.96	4.70	4.78		
24	4.94	4.68	4.76		
25	4.92	4.66	4.74		
26	4.90	4.64	4.71		
27	4.88	4.62	4.70		
28	4.86	4.60	4.68		
29	4.84	4.58	4.66		
30	4.83	4.57	4.64		

Table S33: Vertical electron affinity (in eV) for the lowest singlet state of n-acene, obtained with spin-unrestricted TAO-B97-D4, TAO-B97X-D4, and TAO-ωB97X-D4. For comparison, the experimental (EXP) data are taken from the work of Malloci *et al.*,[12] and the CCSD(T)/CBS data are taken from the work of Hajgatoá *et al.*[14]

n-acene	TAO-B97-D4	TAO-B97X-D4	TAO-ωB97X-D4	CCSD(T)/CBS	EXP
2	-0.19	-0.55	-0.41	-0.48	-0.20
3	0.67	0.33	0.44	0.28	0.53
4	1.27	0.94	1.03	0.82	1.07
5	1.69	1.37	1.44	1.21	1.39
6	2.00	1.68	1.75	1.47	
7	2.22	1.92	1.98		
8	2.38	2.10	2.16		
9	2.52	2.24	2.31		
10	2.63	2.36	2.43		
11	2.73	2.46	2.53		
12	2.81	2.54	2.62		
13	2.88	2.62	2.69		
14	2.95	2.68	2.76		
15	3.01	2.74	2.82		
16	3.06	2.80	2.87		
17	3.10	2.84	2.92		
18	3.15	2.89	2.96		
19	3.19	2.92	3.00		
20	3.22	2.96	3.04		
21	3.25	2.99	3.07		
22	3.28	3.02	3.10		
23	3.31	3.05	3.13		
24	3.34	3.08	3.16		
25	3.36	3.10	3.18		
26	3.38	3.13	3.21		
27	3.41	3.15	3.23		
28	3.43	3.17	3.25		
29	3.45	3.19	3.27		
30	3.46	3.21	3.29		

Table S34: Fundamental gap (in eV) for the lowest singlet state of n-acene, obtained with spin-unrestricted TAO-B97-D4, TAO-B97X-D4, and TAO-ωB97X-D4. For comparison, the experimental (EXP) data are taken from the work of Malloci *et al.*,[12] and the CCSD(T)/CBS data are taken from the works of Deleuze *et al.*[13] and Hajgatoá *et al.*[14]

n-acene	TAO-B97-D4	TAO-B97X-D4	TAO-ωB97X-D4	CCSD(T)/CBS	EXP
2	8.23	8.40	8.32	8.72	8.34
3	6.60	6.74	6.70	7.19	6.91
4	5.48	5.60	5.59	6.13	5.90
5	4.68	4.79	4.81	5.37	5.20
6	4.11	4.21	4.23	4.96	
7	3.70	3.77	3.80		
8	3.39	3.44	3.46		
9	3.14	3.17	3.19		
10	2.93	2.95	2.96		
11	2.75	2.77	2.77		
12	2.59	2.61	2.61		
13	2.46	2.46	2.47		
14	2.33	2.34	2.34		
15	2.23	2.23	2.23		
16	2.13	2.13	2.13		
17	2.04	2.04	2.05		
18	1.96	1.96	1.96		
19	1.89	1.89	1.89		
20	1.82	1.82	1.82		
21	1.76	1.76	1.76		
22	1.70	1.70	1.70		
23	1.65	1.65	1.65		
24	1.60	1.60	1.60		
25	1.55	1.55	1.56		
26	1.51	1.51	1.51		
27	1.47	1.47	1.47		
28	1.43	1.43	1.43		
29	1.40	1.39	1.39		
30	1.36	1.36	1.36		

Table S35: Symmetrized von Neumann entropy for the lowest singlet state of n-acene, obtained with spin-restricted TAO-B97-D4, TAO-B97X-D4, and TAO-ωB97X-D4.

n-acene	TAO-B97-D4	TAO-B97X-D4	TAO-ωB97X-D4
2	0.02	0.20	0.70
3	0.12	0.41	1.10
4	0.36	0.70	1.54
5	0.69	1.04	2.01
6	1.02	1.39	2.49
7	1.31	1.73	2.98
8	1.55	2.05	3.47
9	1.77	2.36	3.94
10	1.99	2.66	4.42
11	2.21	2.97	4.89
12	2.44	3.27	5.36
13	2.67	3.58	5.84
14	2.90	3.89	6.31
15	3.14	4.20	6.79
16	3.37	4.51	7.26
17	3.60	4.81	7.73
18	3.83	5.12	8.21
19	4.06	5.43	8.68
20	4.29	5.74	9.16
21	4.53	6.05	9.63
22	4.76	6.36	10.10
23	4.99	6.67	10.58
24	5.22	6.97	11.05
25	5.45	7.28	11.53
26	5.69	7.59	12.00
27	5.92	7.90	12.47
28	6.15	8.21	12.95
29	6.38	8.52	13.42
30	6.61	8.82	13.90

Table S36: Electronic isomerization energies (in kJ/mol) in the iso-C_{40} database,[2] obtained with spin-unrestricted TAO-BLYP and its dispersion-corrected versions.

Isomer No.	reference	TAO-BLYP	TAO-BLYP-D3(0)	TAO-BLYP-D3(BJ)	TAO-BLYP-D4
2	43.1	42.16	40.16	40.19	37.62
3	65.4	42.73	42.93	43.28	42.24
4	94.3	67.91	67.52	68.80	64.21
5	117.4	104.88	104.98	107.21	103.89
6	142.7	100.27	101.58	102.71	102.10
7	159.9	140.62	139.79	141.20	134.71
8	164.2	137.88	137.12	138.97	131.88
9	167.2	142.71	141.14	142.61	134.62
10	173.4	136.11	135.97	137.99	132.42
11	194.2	169.17	169.00	171.75	164.84
12	198.5	161.25	160.54	162.32	155.66
13	206.3	175.21	173.45	175.24	168.81
14	210.8	176.91	177.12	178.59	172.70
15	216.2	203.50	202.18	203.99	195.10
16	219.5	192.97	191.41	191.71	185.33
17	237.3	216.76	214.92	216.57	208.39
18	241.8	212.33	210.96	211.97	205.55
19	286.2	265.13	262.90	265.61	256.33
20	294.3	274.97	272.82	273.84	264.02
21	332.5	307.93	306.67	309.04	299.81
22	342.6	297.42	295.96	297.42	289.19
23	377.0	330.56	327.95	328.45	316.23
24	388.5	329.80	327.13	328.92	320.12
25	400.4	385.27	382.47	383.64	372.82
26	412.3	378.70	376.26	377.20	367.50
27	448.0	441.72	440.19	443.29	431.12
28	472.6	452.91	448.31	448.12	436.78
29	763.3	703.57	695.46	691.24	676.23

Table S37: Electronic isomerization energies (in kJ/mol) in the iso-C_{40} database,[2] obtained with spin-unrestricted TAO-PBE and its dispersion-corrected versions.

Isomer No.	reference	TAO-PBE	TAO-PBE-D3(0)	TAO-PBE-D3(BJ)	TAO-PBE-D4
2	43.1	39.97	39.45	39.16	37.77
3	65.4	40.94	41.09	41.26	40.77
4	94.3	62.97	63.11	63.56	61.24
5	117.4	104.36	104.66	105.58	103.94
6	142.7	100.08	100.64	101.30	101.10
7	159.9	134.55	134.67	135.10	131.81
8	164.2	131.05	131.26	131.86	128.23
9	167.2	134.58	134.56	134.87	130.74
10	173.4	131.34	131.66	132.44	129.65
11	194.2	164.61	165.04	166.10	162.68
12	198.5	155.27	155.47	156.07	152.72
13	206.3	169.76	169.72	170.11	166.85
14	210.8	171.85	172.32	172.92	170.03
15	216.2	195.90	196.07	196.53	191.96
16	219.5	187.41	187.30	187.16	183.80
17	237.3	210.01	210.01	210.32	206.08
18	241.8	207.56	207.55	207.73	204.43
19	286.2	259.34	259.48	260.06	255.36
20	294.3	266.65	266.71	266.65	261.55
21	332.5	302.10	302.53	303.15	298.60
22	342.6	291.39	291.55	291.85	287.63
23	377.0	318.53	318.67	318.24	311.92
24	388.5	323.07	323.07	323.24	318.75
25	400.4	378.96	379.07	378.88	373.28
26	412.3	372.76	372.84	372.67	367.72
27	448.0	437.13	437.79	438.57	432.48
28	472.6	446.26	446.00	444.86	438.80
29	763.3	694.03	694.00	689.79	681.70

Table S38: Electronic isomerization energies (in kJ/mol) in the iso-C_{40} database,[2] obtained with spin-unrestricted TAO-B3LYP and its dispersion-corrected versions.

Isomer No.	reference	TAO-B3LYP	TAO-B3LYP-D3(0)	TAO-B3LYP-D3(BJ)	TAO-B3LYP-D4
2	43.1	48.97	47.63	47.40	45.50
3	65.4	50.08	50.13	50.55	49.77
4	94.3	80.56	80.27	81.36	77.81
5	117.4	114.83	114.84	116.79	114.12
6	142.7	110.97	111.64	112.98	112.50
7	159.9	160.55	159.90	161.12	156.18
8	164.2	157.08	156.55	158.08	152.61
9	167.2	163.05	162.04	163.09	156.99
10	173.4	154.17	154.02	155.79	151.45
11	194.2	189.05	188.90	191.27	185.91
12	198.5	182.57	182.01	183.55	178.46
13	206.3	197.90	196.67	198.05	193.19
14	210.8	198.26	198.28	199.74	195.28
15	216.2	227.59	226.72	228.14	221.35
16	219.5	215.63	214.54	214.72	209.94
17	237.3	242.30	241.09	242.28	236.06
18	241.8	236.26	235.25	236.08	231.26
19	286.2	295.98	294.48	296.55	289.52
20	294.3	303.94	302.55	303.19	295.83
21	332.5	341.18	340.25	342.26	335.37
22	342.6	327.94	326.91	328.10	321.91
23	377.0	365.36	363.66	363.86	354.81
24	388.5	365.31	363.43	364.79	358.26
25	400.4	422.27	420.46	421.17	413.14
26	412.3	417.62	415.96	416.59	409.45
27	448.0	478.85	477.85	480.38	471.23
28	472.6	494.04	491.20	490.40	482.13
29	763.3	762.13	757.88	752.53	742.20

Table S39: Electronic isomerization energies (in kJ/mol) in the iso-C_{40} database,[2] obtained with spin-unrestricted TAO-PBE0 and its dispersion-corrected versions.

Isomer No.	reference	TAO-PBE0	TAO-PBE0-D3(0)	TAO-PBE0-D3(BJ)	TAO-PBE0-D4
2	43.1	48.52	48.02	47.77	46.70
3	65.4	49.98	50.07	50.32	49.90
4	94.3	78.82	78.90	79.40	77.43
5	117.4	116.22	116.45	117.38	115.96
6	142.7	112.62	113.00	113.81	113.59
7	159.9	159.18	159.22	159.77	157.02
8	164.2	154.95	155.07	155.76	152.70
9	167.2	160.06	160.02	160.40	156.97
10	173.4	153.53	153.76	154.60	152.20
11	194.2	188.88	189.23	190.33	187.42
12	198.5	181.49	181.60	182.31	179.49
13	206.3	197.53	197.41	197.94	195.28
14	210.8	198.01	198.37	199.11	196.66
15	216.2	225.65	225.76	226.34	222.53
16	219.5	215.02	214.90	214.90	212.15
17	237.3	241.35	241.30	241.74	238.24
18	241.8	236.59	236.52	236.85	234.14
19	286.2	296.72	296.70	297.52	293.67
20	294.3	302.21	302.21	302.36	298.19
21	332.5	342.35	342.61	343.49	339.75
22	342.6	328.35	328.45	328.91	325.44
23	377.0	361.60	361.65	361.57	356.43
24	388.5	366.01	365.89	366.35	362.75
25	400.4	423.52	423.55	423.67	419.16
26	412.3	419.63	419.63	419.75	415.77
27	448.0	481.50	481.99	483.05	478.05
28	472.6	495.48	495.23	494.46	489.73
29	763.3	763.84	763.90	760.50	754.53

Table S40: Electronic isomerization energies (in kJ/mol) in the iso-C_{40} database,[2] obtained with spin-unrestricted TAO-BHHLYP and its dispersion-corrected versions.

Isomer No.	reference	TAO-BHHLYP	TAO-BHHLYP-D3(0)	TAO-BHHLYP-D3(BJ)	TAO-BHHLYP-D4
2	43.1	54.81	53.93	53.69	52.21
3	65.4	58.66	58.69	59.04	58.48
4	94.3	96.21	96.09	96.92	94.19
5	117.4	125.92	125.96	127.53	125.45
6	142.7	122.77	123.15	124.35	124.03
7	159.9	184.70	184.41	185.29	181.51
8	164.2	180.87	180.64	181.79	177.57
9	167.2	188.32	187.86	188.53	183.83
10	173.4	175.82	175.80	177.19	173.85
11	194.2	212.78	212.81	214.64	210.53
12	198.5	201.80	201.58	202.71	198.83
13	206.3	223.07	222.32	223.37	219.66
14	210.8	224.02	224.15	225.28	221.93
15	216.2	256.87	256.51	257.49	252.29
16	219.5	241.64	241.14	241.09	237.45
17	237.3	273.07	272.43	273.26	268.49
18	241.8	264.12	263.62	264.15	260.49
19	286.2	332.26	331.33	332.97	327.62
20	294.3	338.28	337.63	337.95	332.35
21	332.5	380.36	379.88	381.44	376.30
22	342.6	364.45	363.96	364.79	360.09
23	377.0	406.66	405.89	405.79	398.99
24	388.5	405.98	404.85	405.85	400.95
25	400.4	465.98	465.04	465.43	459.40
26	412.3	462.81	461.95	462.28	456.95
27	448.0	520.69	520.29	522.20	515.32
28	472.6	534.67	533.16	532.22	526.04
29	763.3	817.21	814.87	810.33	802.97

Table S41: Electronic isomerization energies (in kJ/mol) in the iso-C_{40} database,[2] obtained with spin-unrestricted TAO-LDA, TAO-B97-D4, TAO-B97X-D4, TAO-ωB97X-D4, and KS-ωB97-D4.

Isomer No.	reference	TAO-LDA	TAO-B97-D4	TAO-B97X-D4	TAO-ωB97X-D4	KS-ωB97-D4
2	43.1	37.49	36.67	48.69	52.43	54.75
3	65.4	39.74	40.82	53.28	58.92	78.34
4	94.3	59.91	60.16	83.62	94.94	133.12
5	117.4	104.94	104.26	119.51	123.86	136.64
6	142.7	100.87	102.28	117.94	123.82	164.32
7	159.9	130.61	130.07	165.84	179.82	216.78
8	164.2	126.85	126.62	161.65	175.95	227.42
9	167.2	128.88	128.49	166.52	180.38	216.44
10	173.4	128.87	128.62	160.07	172.14	217.51
11	194.2	162.61	161.54	195.83	208.01	249.30
12	198.5	151.22	151.33	188.91	202.19	227.79
13	206.3	166.14	165.64	204.26	217.44	254.54
14	210.8	168.82	168.92	206.00	218.19	270.54
15	216.2	190.87	189.68	232.94	246.57	284.28
16	219.5	183.77	181.98	220.74	228.97	252.70
17	237.3	205.79	203.84	248.78	262.40	318.79
18	241.8	204.57	202.95	243.42	253.91	295.74
19	286.2	256.13	253.42	305.37	322.20	371.74
20	294.3	261.10	258.60	310.07	322.67	369.79
21	332.5	298.95	296.69	352.74	369.22	414.64
22	342.6	288.18	285.85	337.61	351.00	417.82
23	377.0	310.10	307.50	371.10	384.98	476.73
24	388.5	319.05	317.04	376.27	393.18	448.84
25	400.4	374.76	370.37	432.24	445.14	495.30
26	412.3	368.54	365.56	430.24	447.84	515.30
27	448.0	434.22	430.86	491.23	500.92	525.25
28	472.6	442.45	434.72	503.35	514.69	541.44
29	763.3	688.06	673.89	771.55	782.17	900.49

Table S42: Symmetrized von Neumann entropy for the lowest singlet state of each C_{40} fullerene isomer (No. 1 to No. 29) in the iso-C_{40} database,[2] obtained with spin-restricted TAO-B97-D4, TAO-B97X-D4, and TAO-ωB97X-D4.

Isomer No.	TAO-B97-D4	TAO-B97X-D4	TAO-ωB97X-D4
1	2.03	2.81	4.66
2	1.88	2.75	4.68
3	2.25	3.05	4.91
4	2.15	3.05	5.00
5	2.19	2.94	4.81
6	2.84	3.42	5.17
7	2.03	3.08	5.11
8	2.22	3.15	5.13
9	2.05	3.08	5.11
10	2.38	3.24	5.17
11	2.15	3.09	5.08
12	2.46	3.39	5.31
13	2.02	3.03	5.06
14	2.42	3.36	5.30
15	1.92	3.04	5.11
16	2.15	3.07	5.02
17	2.15	3.21	5.23
18	2.29	3.26	5.20
19	1.87	3.00	5.13
20	2.16	3.26	5.28
21	2.06	3.19	5.30
22	2.55	3.50	5.47
23	2.47	3.63	5.62
24	2.28	3.36	5.42
25	2.01	3.21	5.32
26	2.33	3.46	5.57
27	1.99	3.08	5.19
28	2.25	3.37	5.46
29	3.60	4.52	6.44

Table S43: Symmetrized von Neumann entropy for the lowest singlet state of each C_{40} fullerene isomer (No. 1 to No. 29) in the iso-C_{40} database,[2] obtained with spin-restricted TAO-LDA, TAO-BLYP, TAO-PBE, TAO-B3LYP, TAO-PBE0, and TAO-BHHLYP.

Isomer No.	TAO-LDA	TAO-BLYP	TAO-PBE	TAO-B3LYP	TAO-PBE0	TAO-BHHLYP
1	2.09	2.06	2.06	2.51	2.63	3.32
2	1.92	1.90	1.90	2.41	2.55	3.28
3	2.30	2.28	2.28	2.74	2.86	3.56
4	2.18	2.18	2.16	2.71	2.83	3.60
5	2.24	2.21	2.21	2.63	2.75	3.44
6	2.88	2.86	2.86	3.16	3.26	3.88
7	2.06	2.06	2.04	2.70	2.84	3.67
8	2.26	2.25	2.24	2.80	2.92	3.71
9	2.08	2.08	2.06	2.71	2.84	3.67
10	2.41	2.40	2.39	2.91	3.03	3.77
11	2.19	2.18	2.17	2.74	2.87	3.66
12	2.50	2.50	2.48	3.05	3.17	3.94
13	2.04	2.05	2.03	2.66	2.80	3.61
14	2.46	2.46	2.44	3.01	3.14	3.91
15	1.95	1.96	1.94	2.64	2.78	3.64
16	2.18	2.17	2.16	2.72	2.85	3.62
17	2.19	2.19	2.17	2.83	2.96	3.80
18	2.33	2.32	2.31	2.90	3.03	3.81
19	1.89	1.90	1.88	2.59	2.74	3.62
20	2.19	2.20	2.17	2.87	3.01	3.85
21	2.10	2.11	2.08	2.79	2.93	3.81
22	2.58	2.58	2.56	3.15	3.27	4.05
23	2.50	2.53	2.48	3.24	3.37	4.22
24	2.30	2.31	2.29	2.97	3.11	3.95
25	2.03	2.04	2.02	2.79	2.95	3.83
26	2.35	2.36	2.34	3.06	3.20	4.08
27	2.01	2.01	2.00	2.67	2.82	3.69
28	2.28	2.28	2.26	2.97	3.12	3.98
29	3.61	3.61	3.61	4.17	4.30	5.07

Table S44: The lowest charge-transfer excitation energy (in eV) of $C_2H_4\cdots C_2F_4$ dimer along the intermolecular distance R (in Å), obtained with both spin-restricted (R) and spin-unrestricted (U) calculations employing the KS/TDA (for TAO-B97-D4 with $\theta = 0$) and pTAO/TDA (for TAO-B97-D4 with the optimal system-independent θ (in mhartree), see Table 3) methods. For comparison, the SAC-CI data are taken from the work of Tawada *et al.*[15]

R (Å)	TAO-B97-D4 (R,$\theta = 9.59717$)	TAO-B97-D4 (U,$\theta = 9.59717$)	TAO-B97-D4 (R,$\theta = 0$)	TAO-B97-D4 (U,$\theta = 0$)	SAC-CI
5	4.95	4.95	5.15	5.15	11.49
6	5.00	5.00	5.20	5.20	12.00
7	5.02	5.02	5.22	5.22	12.36
8	5.03	5.03	5.23	5.23	12.63
9	5.04	5.04	5.24	5.24	12.83
10	5.05	5.05	5.24	5.24	12.99

Table S45: The lowest charge-transfer excitation energy (in eV) of $C_2H_4\cdots C_2F_4$ dimer along the intermolecular distance R (in Å), obtained with both spin-restricted (R) and spin-unrestricted (U) calculations employing the KS/TDA (for TAO-B97X-D4 with $\theta = 0$) and pTAO/TDA (for TAO-B97X-D4 with the optimal system-independent θ (in mhartree), see Table 3) methods. For comparison, the SAC-CI data are taken from the work of Tawada *et al.*[15]

R (Å)	TAO-B97X-D4 (R,$\theta = 24.0964$)	TAO-B97X-D4 (U,$\theta = 24.0964$)	TAO-B97X-D4 (R,$\theta = 0$)	TAO-B97X-D4 (U,$\theta = 0$)	SAC-CI
5	8.26	8.26	7.72	7.72	11.49
6	8.46	8.46	7.93	7.93	12.00
7	8.60	8.60	8.06	8.06	12.36
8	8.70	8.70	8.16	8.16	12.63
9	8.77	8.77	8.23	8.23	12.83
10	8.83	8.83	8.29	8.29	12.99

Table S46: The lowest charge-transfer excitation energy (in eV) of $C_2H_4 \cdots C_2F_4$ dimer along the intermolecular distance R (in Å), obtained with both spin-restricted (R) and spin-unrestricted (U) calculations employing the KS/TDA (for TAO-ωB97X-D4 with $\theta = 0$ and for KS-ωB97X-D4) and pTAO/TDA (for TAO-ωB97X-D4 with the optimal system-independent θ (in mhartree), see Table 3) methods. For comparison, the SAC-CI data are taken from the work of Tawada *et al.*[15]

R (Å)	TAO-ωB97X-D4 (R,$\theta = 50.2796$)	TAO-ωB97X-D4 (U,$\theta = 50.2796$)	TAO-ωB97X-D4 (R,$\theta = 0$)	TAO-ωB97X-D4 (U,$\theta = 0$)	KS-ωB97X-D4 (R)	KS-ωB97X-D4 (U)	SAC-CI
5	10.52	10.52	9.83	9.83	9.75	9.75	11.49
6	11.04	11.04	10.35	10.35	10.26	10.26	12.00
7	11.40	11.40	10.71	10.71	10.62	10.62	12.36
8	11.67	11.67	10.98	10.98	10.89	10.89	12.63
9	11.87	11.87	11.18	11.18	11.10	11.10	12.83
10	12.04	12.04	11.35	11.35	11.26	11.26	12.99

Table S47: The lowest charge-transfer excitation energy (in eV) of $Ar \cdots S_3$ dimer along the intermolecular distance R (in Å), obtained with both spin-restricted (R) and spin-unrestricted (U) calculations employing the KS/TDA (for TAO-B97-D4 with $\theta = 0$) and pTAO/TDA (for TAO-B97-D4 with the optimal system-independent θ (in mhartree), see Table 3) methods.

R (Å)	TAO-B97-D4 (R,$\theta = 9.59717$)	TAO-B97-D4 (U,$\theta = 9.59717$)	TAO-B97-D4 (R,$\theta = 0$)	TAO-B97-D4 (U,$\theta = 0$)
5	4.55	4.55	4.81	4.81
6	4.58	4.58	4.85	4.85
7	4.60	4.60	4.87	4.87
8	4.61	4.61	4.88	4.88
9	4.62	4.62	4.89	4.89
10	4.63	4.63	4.90	4.90

Table S48: The lowest charge-transfer excitation energy (in eV) of $Ar \cdots S_3$ dimer along the intermolecular distance R (in Å), obtained with both spin-restricted (R) and spin-unrestricted (U) calculations employing the KS/TDA (for TAO-B97X-D4 with $\theta = 0$) and pTAO/TDA (for TAO-B97X-D4 with the optimal system-independent θ (in mhartree), see Table 3) methods.

R (Å)	TAO-B97X-D4 (R,$\theta = 24.0964$)	TAO-B97X-D4 (U,$\theta = 24.0964$)	TAO-B97X-D4 (R,$\theta = 0$)	TAO-B97X-D4 (U,$\theta = 0$)
5	6.81	6.81	6.95	6.95
6	6.99	6.99	7.14	7.14
7	7.12	7.12	7.27	7.27
8	7.21	7.21	7.37	7.37
9	7.29	7.29	7.44	7.44
10	7.34	7.34	7.50	7.50

Table S49: The lowest charge-transfer excitation energy (in eV) of $Ar \cdots S_3$ dimer along the intermolecular distance R (in Å), obtained with both spin-restricted (R) and spin-unrestricted (U) calculations employing the KS/TDA (for TAO-ωB97X-D4 with $\theta = 0$ and for KS-ωB97X-D4) and pTAO/TDA (for TAO-ωB97X-D4 with the optimal system-independent θ (in mhartree), see Table 3) methods.

R (Å)	TAO-ωB97X-D4 (R,$\theta = 50.2796$)	TAO-ωB97X-D4 (U,$\theta = 50.2796$)	TAO-ωB97X-D4 (R,$\theta = 0$)	TAO-ωB97X-D4 (U,$\theta = 0$)	KS-ωB97X-D4 (R)	KS-ωB97X-D4 (U)
5	9.22	9.22	8.75	9.35	8.69	9.23
6	9.70	9.70	9.19	9.84	9.13	9.72
7	10.06	10.06	9.54	10.20	9.48	10.07
8	10.32	10.32	9.81	10.46	9.75	10.34
9	10.52	10.52	10.02	10.67	9.95	10.55
10	10.69	10.69	10.18	10.84	10.12	10.71

Table S50: The lowest charge-transfer excitation energy (in eV) of $S_3 \cdots Ar$ dimer along the intermolecular distance R (in Å), obtained with both spin-restricted (R) and spin-unrestricted (U) calculations employing the KS/TDA (for TAO-B97-D4 with $\theta = 0$) and pTAO/TDA (for TAO-B97-D4 with the optimal system-independent θ (in mhartree), see Table 3) methods.

R (Å)	TAO-B97-D4 (R,$\theta = 9.59717$)	TAO-B97-D4 (U,$\theta = 9.59717$)	TAO-B97-D4 (R,$\theta = 0$)	TAO-B97-D4 (U,$\theta = 0$)
5	4.74	4.74	5.02	5.02
6	4.70	4.70	4.98	4.98
7	4.69	4.69	4.96	4.96
8	4.68	4.68	4.95	4.95
9	4.67	4.67	4.95	4.95
10	4.67	4.67	4.94	4.94

Table S51: The lowest charge-transfer excitation energy (in eV) of $S_3 \cdots Ar$ dimer along the intermolecular distance R (in Å), obtained with both spin-restricted (R) and spin-unrestricted (U) calculations employing the KS/TDA (for TAO-B97X-D4 with $\theta = 0$) and pTAO/TDA (for TAO-B97X-D4 with the optimal system-independent θ (in mhartree), see Table 3) methods.

R (Å)	TAO-B97X-D4 (R,$\theta = 24.0964$)	TAO-B97X-D4 (U,$\theta = 24.0964$)	TAO-B97X-D4 (R,$\theta = 0$)	TAO-B97X-D4 (U,$\theta = 0$)
5	7.01	7.01	7.18	7.18
6	7.12	7.12	7.29	7.29
7	7.21	7.21	7.38	7.38
8	7.28	7.28	7.45	7.45
9	7.34	7.34	7.50	7.50
10	7.39	7.39	7.55	7.55

Table S52: The lowest charge-transfer excitation energy (in eV) of $S_3 \cdots Ar$ dimer along the intermolecular distance R (in Å), obtained with both spin-restricted (R) and spin-unrestricted (U) calculations employing the KS/TDA (for TAO-ωB97X-D4 with $\theta = 0$ and for KS-ωB97X-D4) and pTAO/TDA (for TAO-ωB97X-D4 with the optimal system-independent θ (in mhartree), see Table 3) methods.

R (Å)	TAO-ωB97X-D4 (R,$\theta = 50.2796$)	TAO-ωB97X-D4 (U,$\theta = 50.2796$)	TAO-ωB97X-D4 (R,$\theta = 0$)	TAO-ωB97X-D4 (U,$\theta = 0$)	KS-ωB97X-D4 (R)	KS-ωB97X-D4 (U)
5	9.41	9.41	8.93	9.63	8.92	9.46
6	9.83	9.83	9.35	10.00	9.29	9.88
7	10.13	10.13	9.66	10.31	9.60	10.19
8	10.38	10.38	9.90	10.55	9.84	10.43
9	10.57	10.57	10.09	10.74	10.02	10.62
10	10.73	10.73	10.24	10.89	10.17	10.77

References

(1) Levine, D. S.; Hait, D.; Tubman, N. M.; Lehtola, S.; Whaley, K. B.; Head-Gordon, M. CASSCF with extremely large active spaces using the adaptive sampling configuration interaction method. *J. Chem. Theory Comput.* **2020**, 16, 2340–2354.

(2) Karton, A. Fullerenes pose a strain on hybrid density functional theory. *J. Phys. Chem. A* **2022**, 126, 4709–4720.

(3) Goerigk, L.; Hansen, A.; Bauer, C.; Ehrlich, S.; Najibi, A.; Grimme, S. A look at the density functional theory zoo with the advanced GMTKN55 database for general main group thermochemistry, kinetics and noncovalent interactions. *Phys. Chem. Chem. Phys.* **2017**, 19, 32184–32215.

(4) Lin, Y.-S.; Li, G.-D.; Mao, S.-P.; Chai, J.-D. Long-range corrected hybrid density functionals with improved dispersion corrections. *J. Chem. Theory Comput.* **2013**, 9, 263–272.

(5) DiStasio, R. A. Jr.; Steele, R. P.; Rhee, Y. M.; Shao, Y.; Head-Gordon, M. An improved algorithm for analytical gradient evaluation in resolution-of-the-identity second-order Møller-Plesset perturbation theory: Application to alanine tetrapeptide conformational analysis. *J. Comput. Chem.* **2007**, 28, 839–856.

(6) Birks, J. *Photophysics of Aromatic Molecules*; John Wiley & Sons, Inc, 1970.

(7) Schiedt, J.; Weinkauf, R. Photodetachment photoelectron spectroscopy of mass selected anions: anthracene and the anthracene-H2O cluster. *Chem. Phys. Lett.* **1997**, 266, 201–205.

(8) Sabbatini, N.; Indelli, M. T.; Gandolfi, M. T.; Balzani, V. Quenching of singlet and triplet excited states of aromatic molecules by europium ions. *J. Phys. Chem.* **1982**, 86, 3585–3591.

(9) Burgos, J.; Pope, M.; Swenberg, C. E.; Alfano, R. R. Heterofission in pentacene-doped tetracene single crystals. *Phys. Status Solidi B* **1977**, 83, 249–256.

(10) Hachmann, J.; Dorando, J. J.; Avilés, M.; Chan, G. K.-L. The radical character of the acenes: A density matrix renormalization group study. *J. Chem. Phys.* **2007**, 127, 134309.

(11) Schriber, J. B.; Hannon, K. P.; Li, C.; Evangelista, F. A. A combined selected configuration interaction and many-body treatment of static and dynamical correlation in oligoacenes. *J. Chem. Theory Comput.* **2018**, 14, 6295–6305.

(12) Malloci, G.; Mulas, G.; Cappellini, G.; Joblin, C. Time-dependent density functional study of the electronic spectra of oligoacenes in the charge states −1, 0, +1, and +2. *Chem. Phys.* **2007**, 340, 43–58.

(13) Deleuze, M. S.; Claes, L.; Kryachko, E. S.; François, J. P. Benchmark theoretical study of the ionization threshold of benzene and oligoacenes. *J. Chem. Phys.* **2003**, 119, 3106–3119.

(14) Hajgató, B.; Deleuze, M. S.; Tozer, D. J.; De Proft, F. A benchmark theoretical study of the electron affinities of benzene and linear acenes. *J. Chem. Phys.* **2008**, 129, 084308.

(15) Tawada, Y.; Tsuneda, T.; Yanagisawa, S.; Yanai, T.; Hirao, K. A long-range-corrected time-dependent density functional theory. *J. Chem. Phys.* **2004**, 120, 8425–8433.
